# Supplementary figures and images for: What we can and cannot see from the surveillance for drug-resistant Pseudomonas aeruginosa—Findings from the evaluation of a surveillance system for multidrug-resistant P. aeruginosa infections in Japan
Source: PLoS One. 2025 Aug 4;20(8):e0329635. doi: 10.1371/journal.pone.0329635 (PMC12321080; doi:10.1371/journal.pone.0329635)

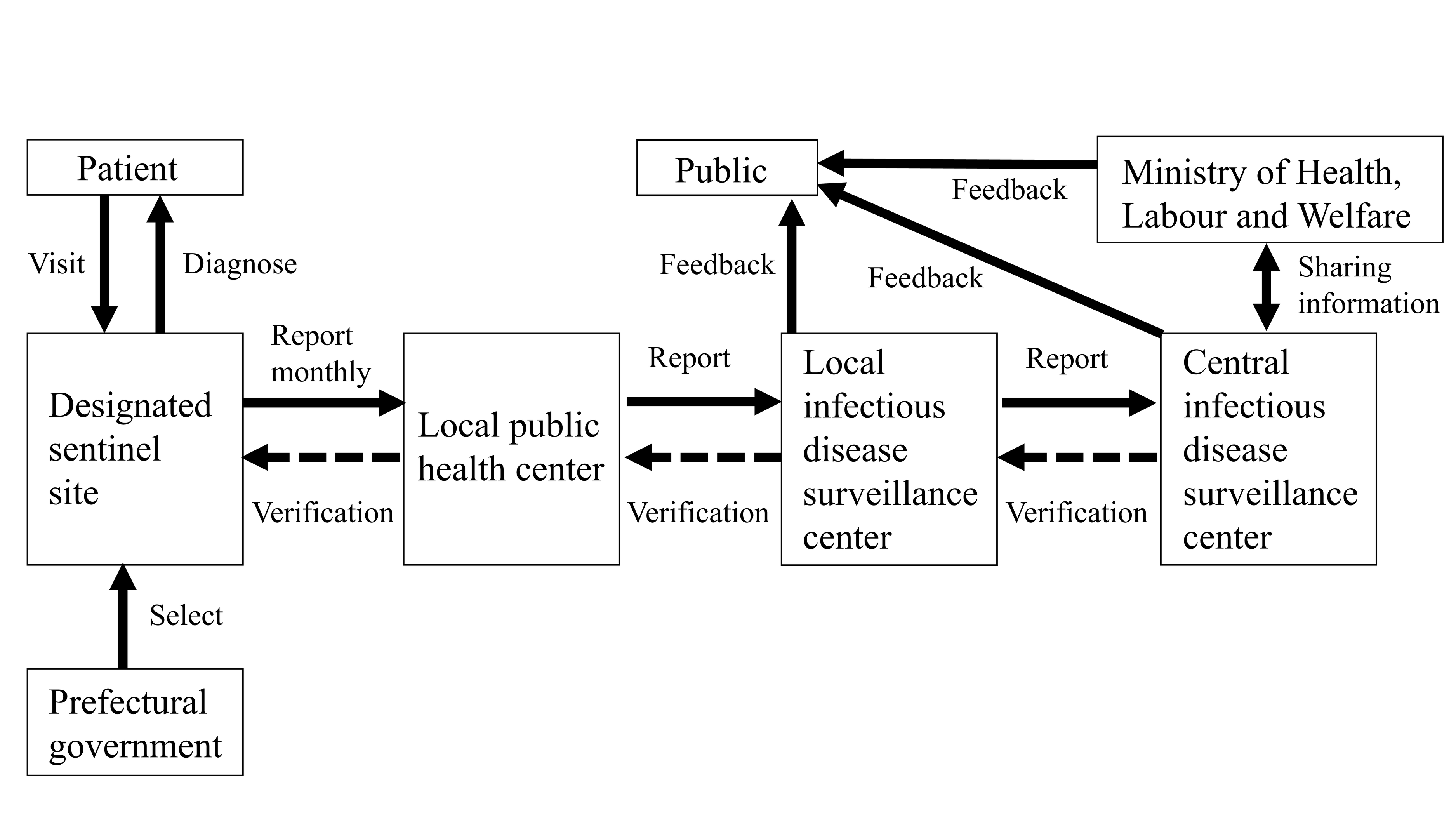

Supplement: S1 Fig — The sentinel sites report the MDRP infections with the information of age, sex, and detected specimen to the local public health center monthly. The central infectious disease surveillance center integrates the data and shares them with the Ministry of Health, Labour and Welfare. MDRP, multidrug-resistant Pseudomonas aeruginosa. (TIF) [file pone.0329635.s001.tif]

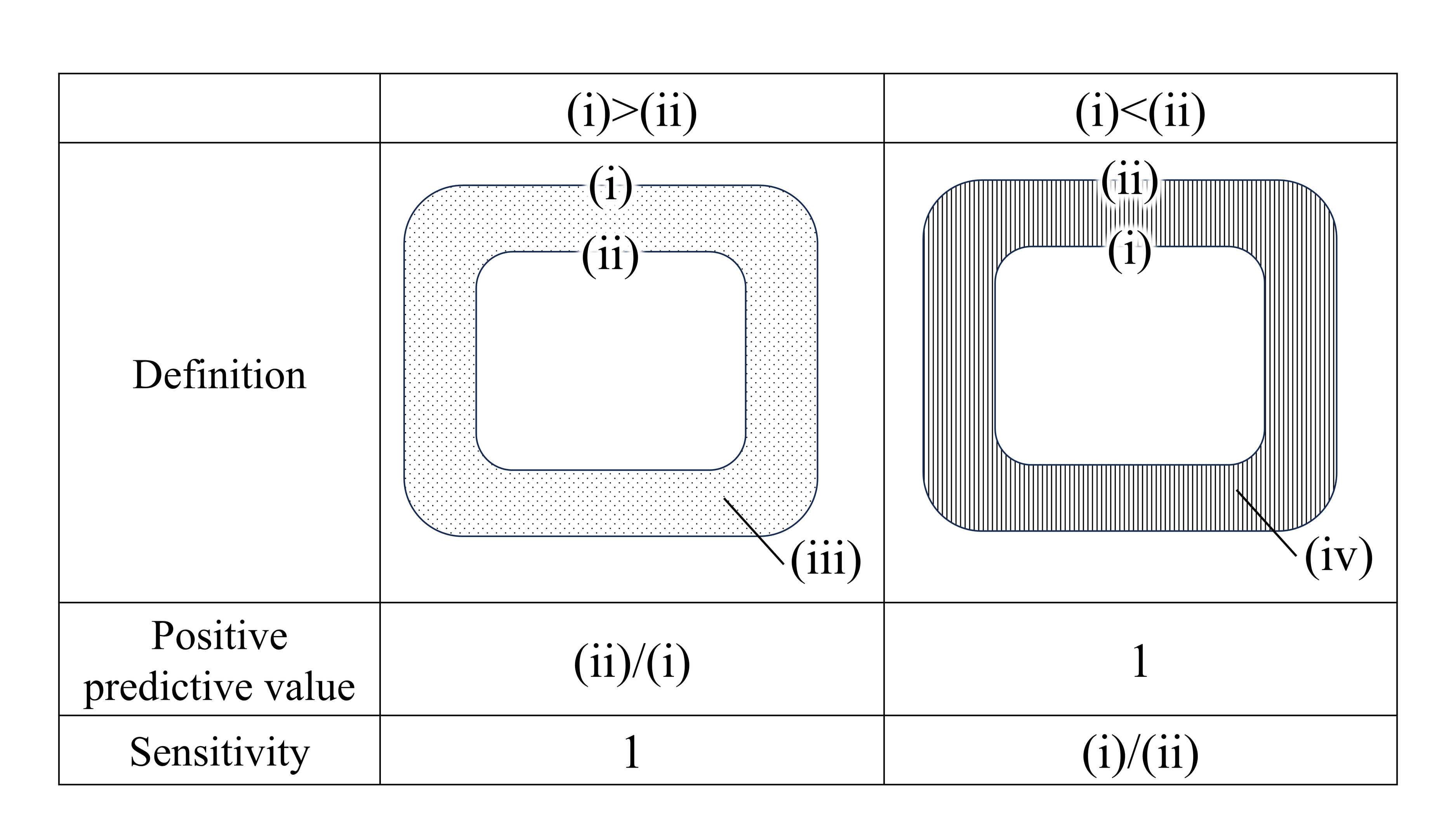

Supplement: S2 Fig — (i) The number of cases reported as MDRP infections. (ii) The accurate number of MDRP infections. (iii) The number of cases misreported as MDRP infections. (iv) The number of underreported accurate MDRP infections. MDRP, multidrug-resistant Pseudomonas aeruginosa. (TIF) [file pone.0329635.s002.tif]

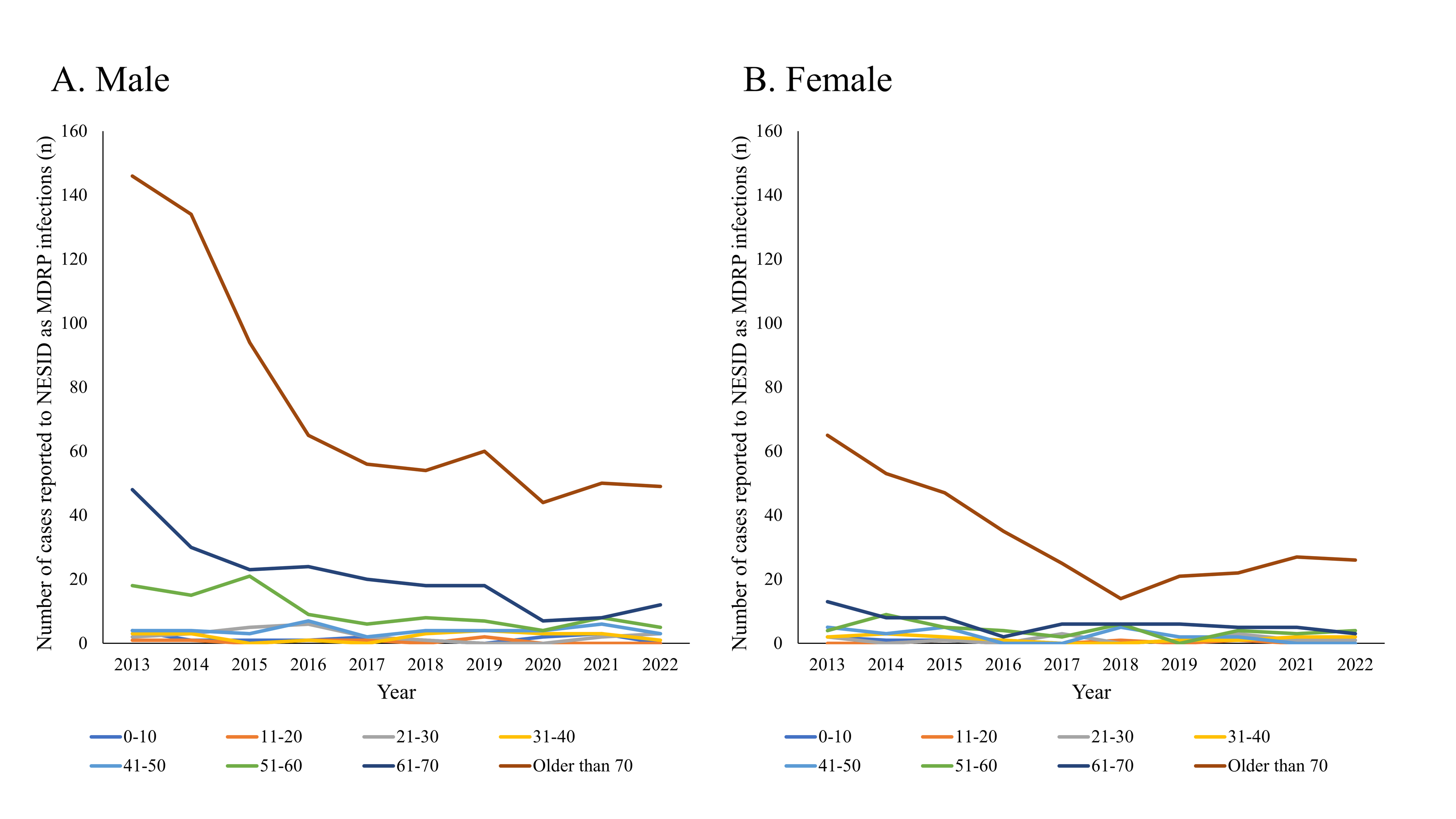

Supplement: S3 Fig — The number of reported cases of MDRP infection showed a decreasing trend in both males (A) and females (B), especially among the elderly. MDRP, multidrug-resistant Pseudomonas aeruginosa; NESID, National Epidemiological Surveillance of Infectious Diseases. (TIF) [file pone.0329635.s003.tif]
