# Supplementary material for: What we can and cannot see from the surveillance for drug-resistant Pseudomonas aeruginosa—Findings from the evaluation of a surveillance system for multidrug-resistant P. aeruginosa infections in Japan
Source: PLoS One. 2025 Aug 4;20(8):e0329635. doi: 10.1371/journal.pone.0329635 (PMC12321080; doi:10.1371/journal.pone.0329635)
Supplement: S1 Appendix — (DOCX) [file pone.0329635.s009.docx]

**S1 Appendix. Questionnaire on the Testing and Reporting System for Multidrug-resistant *Pseudomonas aeruginosa* (MDRP)**

Date of record

**Background**

1. Hospital Prefecture

2. Name Occupation

Phone Number 　　　　　　　 E -mail

3. Number of beds in your hospital

4. Presence of Infection Control Specialist: Yes/No

If your answer is "Yes", please describe their occupations.

Infection control doctor/Certified nurse in infection control/Board certified infection control pharmacy specialist/Infection control microbiological technologist/Others ( ) /Unknown

5. Presence of microbiology laboratory: Yes / No

If your answer is "No", please describe the name of the outsourced microbiology testing company.
( )

6. Number of bacteriology technologists working at hospital

7. Personnel responsible for registering patient information to NESID

Administrative staff/Laboratory technician/Nurse/Doctor/Others ( )/Unknown

8. Using antimicrobial susceptibility testing criteria

CLSI ( Ver.)/EUCAST ( Ver.)/Others ( )/Unknown

9. Using testing equipment for antimicrobial susceptibility

DxM Microscan WalkAway(Beckman Coulter)/VITEK (bioMérieux)/Others ( )

10. Presence of in-hospital testing for antimicrobial resistance genes: Yes/No/Unknown

If your answer is "Yes", please describe which antimicrobial resistance genes you can examine.

( )

11. If you have any opinions on the detection or reporting of MDRP, please write them down.

*NESID* the National Epidemiological Surveillance of Infectious Diseases

*CLSI* the Clinical and Laboratory Standards Institute

*EUCAST* the European Committee on Antimicrobial Susceptibility Testing

**The detection and reporting status of MDRP**

|  |  | 2018 | 2019 | 2020 | 2021 | 2022 |
| --- | --- | --- | --- | --- | --- | --- |
| 1) | The number of detections of *P. aeruginosa* | cases | cases | cases | cases | cases |
| 2) | Of the answers to 1), the number of *P. aeruginosa* that met reporting criteria^*^ for MDRP | cases | cases | cases | cases | cases |
| 3) | Of the answers to 2), the number of patients detected with MDRP | cases | cases | cases | cases | cases |
| 4) | Of the answers to 3), the number of cases of MDRP infections excluding colonization^†^ | cases | cases | cases | cases | cases |
| 5) | The number of reported cases of MDRP infections to NESID actually | cases | cases | cases | cases | cases |
| 6) | Of the answers to 5), the number of cases that did not meet reporting criteria and a breakdown of those cases | cases | cases | cases | cases | cases |
|  | a. At least one of the three drugs did not meet the criteria | cases | cases | cases | cases | cases |
|  | Specific drug not meeting the criteria | cases | cases | cases | cases | cases |
|  | a-1 Carbapenems | cases | cases | cases | cases | cases |
|  | a-2 Amikacin | cases | cases | cases | cases | cases |
|  | a-3 Fluoroquinolones | cases | cases | cases | cases | cases |
|  | b. The patient was a colonized case. | cases | cases | cases | cases | cases |
|  | c. The organisms was not *P. aeruginosa*. | cases | cases | cases | cases | cases |
|  | d. Others | cases | cases | cases | cases | cases |

^*^ Please refer to the reporting criteria for MDRP infections published by the Ministry of Health, Labour and Welfare in Japan (https://www.mhlw.go.jp/bunya/kenkou/kekkaku-kansenshou11/01-05-42-01.html).

^†^ ”Cases of MDRP infection excluding colonization” are defined as either (1) when the attending physician diagnoses an infection, or (2) when effective antimicrobial treatment is prescribed in the electronic medical records after susceptibility test results have been reported.
