# Supplementary material for: What we can and cannot see from the surveillance for drug-resistant Pseudomonas aeruginosa—Findings from the evaluation of a surveillance system for multidrug-resistant P. aeruginosa infections in Japan
Source: PLoS One. 2025 Aug 4;20(8):e0329635. doi: 10.1371/journal.pone.0329635 (PMC12321080; doi:10.1371/journal.pone.0329635)
Supplement: S2 Appendix — (DOCX) [file pone.0329635.s010.docx]

**S2 Appendix. Interview guides of surveillance evaluation for multi-drug resistant *Pseudomonas aeruginosa* (MDRP)**

Interview Date:

Institution Name: (Designated sentinel site / Non-designated sentinel site)

Occupations of Interviewee:

**Contents of interview**

**Questions for all interviewees**

I. What do you think about the necessity of reporting MDRP infections? (Acceptability)

II. What do you think the designated sentinel sites report cases of MDRP infections monthly?

(Acceptability)

III. Do you think it easy to report and confirm a case by using the system? (Simplicity)

IV. Do you think the case definition is easy to understand? (Simplicity)

V. Do you think designated sentinel sites are sufficient for reporting MDRP infections?

(Representativeness)

VI. Do you think the system effectively prevent incorrect or missing reports?

(Positive predictive value)

VII. Do you utilize the surveillance system for MDRP to combat antimicrobial resistance?

(Usefulness)

VIII. Which do you think is better for the surveillance system for MDRP infections, notifiable diseases surveillance or sentinel surveillance?

IX. Which do you think is better as a reportable disease: MDRP infections or carbapenem-resistant *P. aeruginosa* infections?

**Specific questions for interviewees working at hospitals**

I. Who is responsible for reporting to the National Epidemiological Surveillance of Infectious Diseases?

II. How are patients with notifiable infectious diseases detected and reported?

**Specific questions for interviewees working at the local infectious disease surveillance center**

I. Do you think designated sentinel sites in your prefecture appropriately based on criteria?

(Representativeness)

II. Do you think information is promptly provided to the public after a physician diagnoses a patient? (Timeliness)

III. Do you think the case definition can be changed easily? (Flexibility)

IV. Do you think the designated sentinel sites can add or change the reporting contents freely?

(Flexibility)

V. Do you think the prefecture can change the designated sentinel sites easily? (Flexibility)

VI. Do you think the surveillance system can continue to function after unexpected accidents?

(Stability)
