# Supplementary material for: What we can and cannot see from the surveillance for drug-resistant Pseudomonas aeruginosa—Findings from the evaluation of a surveillance system for multidrug-resistant P. aeruginosa infections in Japan
Source: PLoS One. 2025 Aug 4;20(8):e0329635. doi: 10.1371/journal.pone.0329635 (PMC12321080; doi:10.1371/journal.pone.0329635)
Supplement: Attachment — Submitted filename: PONE-D-25-07682 rev AAO.pdf [file pone.0329635.s011.pdf]

PLOS ONE

What we can and cannot see from the surveillance for drug-resistant Pseudomonas aeruginosa - Findings from the evaluation of surveillance system for multidrug-resistant P. aeruginosa infections in Japan -

--Manuscript Draft--

Manuscript Number:	PONE-D-25-07682
Article Type:	Research Article
Full Title:	What we can and cannot see from the surveillance for drug-resistant Pseudomonas aeruginosa - Findings from the evaluation of surveillance system for multidrug-resistant P. aeruginosa infections in Japan -
Short Title:	Surveillance evaluation for multidrug-resistant Pseudomonas aeruginosa infections
Corresponding Author:	Takuya Yamagishi, Dr<br>National Institute of Infectious Diseases: Kokuritsu Kansensho Kenkyujo<br>Tokyo, JAPAN
Keywords:	Antimicrobial resistance, Carbapenems, Infection control, Pseudomonas aeruginosa, Surveillance system
Abstract:	Introduction<br>Antimicrobial resistance in Pseudomonas aeruginosa is a global health concern, and some countries and regions have been monitoring drug resistance in P. aeruginosa with various surveillance systems. Japan monitors multidrug-resistant P. aeruginosa (MDRP) infections through a national sentinel surveillance system, which has shown a recent decline in MDRP reports. We evaluated the surveillance system to verify the validity of this trend and explore future directions.<br>Method<br>We conducted a cross-sectional study mainly based on the guidelines published in 2001. As a quantitative method, we analyzed characteristics of reports on MDRP infections from designated sentinel sites (DSSs) between 2013–2022. A questionnaire was sent to identifiable DSSs (target DSSs) requesting data on accurate numbers of MDRP infections between 2018–2022. We compared the number of cases reported on national surveillance and the questionnaire. Additionally, we conducted face-to-face interviews with surveillance system stakeholders to assess its usefulness and challenges as a qualitative method.<br>Results<br>From 2013 to 2022, 1,666 cases of MDRP infections were reported by 463 target DSSs. We obtained valid responses to the questionnaire survey from 229 target DSSs (49.5%). From 2018 to 2022, these sites reported 275 cases as MDRP infections, of which 182 were accurate cases of MDRP infection, with both numbers declining over time. False reporting and underreporting of MDRP infections were common, resulting in a positive predictive value of 0.44 and a sensitivity of 0.65 for the reports of MDRP infections on the surveillance system. The interviews highlighted the difficulties in timely detection, accurate reporting, and international data comparison.<br>Discussion<br>Our evaluation indicated that the current sentinel surveillance system for MDRP infections partially captured the true decreasing trend in Japan. However, as the epidemiology of drug-resistant P. aeruginosa is changing, national policy and surveillance strategies may need to address changing public health needs.
Order of Authors:	Shogo Otake, MD
	Takuya Yamagishi, Dr
	Takayuki Shiomoto, PhD
	Manami Nakashita, PhD
	Hitomi Kurosu, PhD
	Chiaki Ikenoue, PhD
	Hirofumi Kato, PhD

Powered by Editorial Manager® and ProduXion Manager® from Aries Systems Corporation

	Munehisa Fukusumi, PhD
	Tomoe Shimada, PhD
	Takuri Takahashi, PhD
	Motoi Suzuki, PhD
	Teruo Kirikae, PhD
	Yoshichika Arakawa, PhD
	Kandai Kozu, PhD
	Tomimasa Sunagawa, PhD
	Motoyuki Sugai, PhD
**Additional Information:**	
**Question**	**Response**
**Financial Disclosure**<br><br>Enter a financial disclosure statement that describes the sources of funding for the work included in this submission. Review the submission guidelines for detailed requirements. View published research articles from *PLOS ONE* for specific examples.<br><br>This statement is required for submission and **will appear in the published article** if the submission is accepted. Please make sure it is accurate.<br><br>**Funded studies**<br>Enter a statement with the following details:<br>• Initials of the authors who received each award<br>• Grant numbers awarded to each author<br>• The full name of each funder<br>• URL of each funder website<br>• Did the sponsors or funders play any role in the study design, data collection and analysis, decision to publish, or preparation of the manuscript?<br><br>Did you receive funding for this work?	Yes
Please add funding details.<br>as follow-up to "**Financial Disclosure**<br><br>Enter a financial disclosure statement that describes the sources of funding for the work included in this submission. Review	Grant from Ministry of Health, Labour, and Welfare, Japan

the submission guidelines for detailed requirements. View published research articles from *PLOS ONE* for specific examples.<br><br>This statement is required for submission and **will appear in the published article** if the submission is accepted. Please make sure it is accurate.<br><br>**Funded studies**<br>Enter a statement with the following details:<br>• Initials of the authors who received each award<br>• Grant numbers awarded to each author<br>• The full name of each funder<br>• URL of each funder website<br>• Did the sponsors or funders play any role in the study design, data collection and analysis, decision to publish, or preparation of the manuscript?<br><br>Did you receive funding for this work?"	
Please select the country of your main research funder (please select carefully as in some cases this is used in fee calculation).<br>as follow-up to "**Financial Disclosure**<br><br>Enter a financial disclosure statement that describes the sources of funding for the work included in this submission. Review the submission guidelines for detailed requirements. View published research articles from *PLOS ONE* for specific examples.<br><br>This statement is required for submission and **will appear in the published article** if the submission is accepted. Please make sure it is accurate.	JAPAN - JP

**Funded studies**<br>Enter a statement with the following details:<br>• Initials of the authors who received each award<br>• Grant numbers awarded to each author<br>• The full name of each funder<br>• URL of each funder website<br>• Did the sponsors or funders play any role in the study design, data collection and analysis, decision to publish, or preparation of the manuscript?<br><br>Did you receive funding for this work?"	
**Competing Interests**<br><br>Use the instructions below to enter a competing interest statement for this submission. On behalf of all authors, disclose any competing interests that could be perceived to bias this work—acknowledging all financial support and any other relevant financial or non-financial competing interests.<br><br>This statement is required for submission and **will appear in the published article** if the submission is accepted. Please make sure it is accurate and that any funding sources listed in your Funding Information later in the submission form are also declared in your Financial Disclosure statement.<br><br>View published research articles from *PLOS ONE* for specific examples.	The authors have declared that no competing interests exist.

**NO authors have competing interests**<br><br>Enter: *The authors have declared that no competing interests exist.*<br><br>**Authors with competing interests**<br><br>Enter competing interest details beginning with this statement:<br><br>*I have read the journal's policy and the authors of this manuscript have the following competing interests: [insert competing interests here]*<br><br>* typeset	
**Ethics Statement**<br><br>Enter an ethics statement for this submission. This statement is required if the study involved:<br><br>• Human participants<br>• Human specimens or tissue<br>• Vertebrate animals or cephalopods<br>• Vertebrate embryos or tissues<br>• Field research<br><br>Write "N/A" if the submission does not require an ethics statement.<br><br>General guidance is provided below. Consult the submission guidelines for detailed instructions. **Make sure that all information entered here is included in the Methods section of the manuscript.**	The ethics committee of National Institute of Infectious Diseases, Japan approved the study. Because the information handled in this study does not lead to the identification of individuals, the National Institute of Infectious Diseases did not require informed consent or ethical review (receipt no. 1478).

Powered by Editorial Manager® and ProduXion Manager® from Aries Systems Corporation

**Format for specific study types**<br><br>**Human Subject Research (involving human participants and/or tissue)**<br>• Give the name of the institutional review board or ethics committee that approved the study<br>• Include the approval number and/or a statement indicating approval of this research<br>• Indicate the form of consent obtained (written/oral) or the reason that consent was not obtained (e.g. the data were analyzed anonymously)<br><br>**Animal Research (involving vertebrate animals, embryos or tissues)**<br>• Provide the name of the Institutional Animal Care and Use Committee (IACUC) or other relevant ethics board that reviewed the study protocol, and indicate whether they approved this research or granted a formal waiver of ethical approval<br>• Include an approval number if one was obtained<br>• If the study involved *non-human primates*, add *additional details* about animal welfare and steps taken to ameliorate suffering<br>• If anesthesia, euthanasia, or any kind of animal sacrifice is part of the study, include briefly which substances and/or methods were applied<br><br>**Field Research**<br><br>Include the following details if this study involves the collection of plant, animal, or other materials from a natural setting:<br>• Field permit number<br>• Name of the institution or relevant body that granted permission	
**Data Availability**<br><br>Authors are required to make all data underlying the findings described fully available, without restriction, and from the time of publication. PLOS allows rare exceptions to address legal and ethical concerns. See the PLOS Data Policy and FAQ for detailed information.	No - some restrictions will apply

Powered by Editorial Manager® and ProduXion Manager® from Aries Systems Corporation

A Data Availability Statement describing where the data can be found is required at submission. Your answers to this question constitute the Data Availability Statement and **will be published in the article**, if accepted.<br><br>**Important:** Stating 'data available on request from the author' is not sufficient. If your data are only available upon request, select 'No' for the first question and explain your exceptional situation in the text box.<br><br>Do the authors confirm that all data underlying the findings described in their manuscript are fully available without restriction?	
**Describe where the data may be found in full sentences. If you are copying our sample text, replace any instances of XXX with the appropriate details.**<br><br>• If the data are **held or will be held in a public repository**, include URLs, accession numbers or DOIs. If this information will only be available after acceptance, indicate this by ticking the box below. For example: *All XXX files are available from the XXX database (accession number(s) XXX, XXX.)*.<br>• If the data are all contained **within the manuscript and/or Supporting Information files**, enter the following: *All relevant data are within the manuscript and its Supporting Information files.*<br>• If neither of these applies but you are able to provide **details of access elsewhere**, with or without limitations, please do so. For example:<br><br>*Data cannot be shared publicly because of [XXX]. Data are available from the XXX Institutional Data Access / Ethics Committee (contact via XXX) for researchers who meet the criteria for access to confidential data.*<br><br>*The data underlying the results presented in the study are available from (include the name of the third party*	Data we collected can be shared publicly, but data on the National Epidemiological Surveillance of Infectious Diseases cannot be shared publicly because the data are strictly protected by the law and need for the governmental clearance for use.

and contact information or URL).<br>• This text is appropriate if the data are owned by a third party and authors do not have permission to share the data.<br><br>* typeset	
Additional data availability information:	Tick here if the URLs/accession numbers/DOIs will be available only after acceptance of the manuscript for publication so that we can ensure their inclusion before publication.

Powered by Editorial Manager® and ProduXion Manager® from Aries Systems Corporation

Manuscript

Click here to access/download;Manuscript;Title_Main_text_PLOS

RESEARCH ARTICLE

What we can and cannot see from the surveillance for drug-resistant *Pseudomonas aeruginosa* - Findings from the evaluation of surveillance system for multidrug-resistant *P. aeruginosa* infections in Japan -

Short Title: Surveillance evaluation for MDRP infections

Shogo Otake[1,2], Takuya Yamagishi[3,4], Takayuki Shiomoto[2], Manami Nakashita[3,4], Hitomi Kurosu[3,4], Chiaki Ikenoue[4], Hirofumi Kato[4], Munehisa Fukusumi[4], Tomoe Shimada[4], Takuri Takahashi[5], Motoi Suzuki[5], Teruo Kirikae[6], Yoshichika Arakawa[7], Kandai Nozu[1], Tomimasa Sunagawa[4], Motoyuki Sugai[3]

[1]Kobe University Graduate School of Medicine, Hyogo, Japan

[2]Field Epidemiology Training Program, National Institute of Infectious Diseases, Tokyo, Japan

[3]Antimicrobial Resistance Research Center, National Institute of Infectious Diseases, Tokyo, Japan

[4]Center for Field Epidemiology Intelligence, Research, and Professional Development, National Institute of Infectious Diseases, Tokyo, Japan

[5]Center for Surveillance, Immunization, and Epidemiologic Research, National Institute of Infectious Diseases, Tokyo, Japan

[6]AMR Research Laboratory, Juntendo Advanced Research Institute for Health Science, Juntendo University, Tokyo, Japan

[7]Department of Microbiology, Fujita Health University School of Medicine, Aichi,

Japan

Corresponding Author: Takuya Yamagishi
Center for Field Epidemic Intelligence, Research and Professional Development, National Institute of Infectious Diseases, J1601 Iidabashi Plano Stage Bldg., 2-7-2 Fujimi, Chiyoda-ku, Tokyo 102-0071, Japan.
E-mail: tack-8@niid.go.jp

Abstract

Introduction

Antimicrobial resistance in *Pseudomonas aeruginosa* is a global health concern, and some countries and regions have been monitoring drug resistance in *P. aeruginosa* with various surveillance systems. Japan monitors multidrug-resistant *P. aeruginosa* (MDRP) infections through a national sentinel surveillance system, which has shown a recent decline in MDRP reports. We evaluated the surveillance system to verify the validity of this trend and explore future directions.

Method

We conducted a cross-sectional study mainly based on the guidelines published in 2001. As a quantitative method, we analyzed characteristics of reports on MDRP infections from designated sentinel sites (DSSs) between 2013–2022. A questionnaire was sent to identifiable DSSs (target DSSs) requesting data on accurate numbers of MDRP infections between 2018–2022. Additionally, we conducted face-to-face interviews with surveillance system stakeholders to assess its usefulness and challenges as a qualitative method.

Results

From 2013 to 2022, 1,666 cases of MDRP infections were reported by 463 target DSSs. We obtained valid responses to the questionnaire survey from 229 target DSSs (49.5%). From 2018 to 2022, these sites reported 275 cases as MDRP infections, of which 182 were accurate cases of MDRP infection, with both numbers declining over time. False reporting and underreporting of MDRP infections were common, resulting in a positive predictive value of 0.44 and a sensitivity of 0.65 for the reports of MDRP infections to

the surveillance system. The interviews highlighted the difficulties in timely detection, accurate reporting, and international data comparison.

Discussion

Our evaluation indicated that the current sentinel surveillance system for MDRP infections partially captured the true decreasing trend in Japan. However, as the epidemiology of drug-resistant *P. aeruginosa* is changing, national policy and surveillance strategies may [illegible]d to address changing public health needs.

Keywords: Antimicrobial resistance, Carbapenems, Infection control, *Pseudomonas aeruginosa*, Surveillance system

Introduction

Pseudomonas aeruginosa is a Gram-negative bacterium responsible for opportunistic infections in immunocompromised hosts [1] that is frequently associated with healthcare-acquired infections [2]. Additionally, it is also a major public health concern, as evidenced by the outbreak linked to artificial tears in the United States (U.S.) in 2023 [3].

Antimicrobial resistance (AMR) of *P. aeruginosa* is recognized as a global health concern. *P. aeruginosa* has multiple mechanisms for acquiring antibiotic resistance [4] that can lead to a serious prognosis [5, 6]. In particular, carbapenem-resistant *P. aeruginosa* (CRPA) and multidrug-resistant *P. aeruginosa* (MDRP) have higher mortality rates compared to susceptible strains of *P. aeruginosa* [5-8]. Thus, the World Health Organization (WHO) listed CRPA as a pathogen of critical priority in 2017 [9]. Although CRPA has been moved from critical to high priority in the 2024 update, innovative research and development of antibiotics remain needed due to the significant burden in high-income countries and several regions [10]. The U.S. Centers for Disease Control and Prevention (CDC) has estimated 32,600 cases and 2,700 deaths due to MDRP infections annually and classified them as a serious public health threat, calling for the need for prompt and sustained action in 2019 [11].

Some countries and regions conduct surveillance for drug-resistant *P. aeruginosa*. The European Antimicrobial Resistance Surveillance Network (EARS-Net) collects AMR data from European Union and European Economic Area countries through the European Surveillance System and annually reports the proportions of MDRP within the WHO European Region and CRPA in each country [12]. In the U. S., the CDC tracks carbapenemase enzymes in CRPA by using data generated by the Antibiotic

Resistance Laboratory Network (AR Lab Network) and CDC laboratories [13, 14].

Japan has two national surveillance system for drug-resistant *P. aeruginosa* [15]. First, the National Epidemiological Surveillance of Infectious Diseases (NESID) was launched in April 1999 based on the "Act on the Prevention of Infectious Diseases and Medical Care for Patients with Infectious Diseases". In this system, the sentinel sites report the MDRP infections with the information of age, sex, and detected specimen to the local public health center monthly to assess the nationwide trend in the number of MDRP infections. The central infectious disease surveillance center (National Institute of Infectious Diseases) integrates the data and shares it with the Ministry of Health, Labour and Welfare (**S1 Fig**) [16, 17]. Second, the Japan Nosocomial Infections Surveillance (JANIS) was implemented in 2000 as a voluntary surveillance system designed to specifically monitor infection and colonization in healthcare settings. This system is a national statistical survey based on the "Statistics Act" and has collected all routine microbiological test results for more than eight million clinical samples annually from over 2,000 facilities as of 2022, the data format of which is uniform even though each hospital uses a different antimicrobial resistance testing system [15, 18, 19]. In both NESID and JANIS, MDRP is defined as *P. aeruginosa* resistant to three anti-pseudomonas antibiotics: carbapenems, amikacin, and fluoroquinolones [20]. To survey long-term trends in antimicrobial susceptibilities among relevant pathogenic bacteria, the susceptibility criteria used by NESID and JANIS for sentinel surveillances differ somewhat from the clinical breakpoints established by the Clinical and Laboratory Standards Institute (CLSI) and the European Committee on Antimicrobial Susceptibility Testing (EUCAST) (**S1 Table**) [21, 22].

The number of MDRP infections reported from sentinel sites to NESID peaked in

2003 at 759 cases (1.62 per sentinel site). Since then, the number has decreased, with 103 cases reported in 2022 (0.22 per sentinel site) [23]. It is thus necessary to evaluate whether this trend accurately represents the actual trend in MDRP infections nationwide. In 2023, the AMR national action plan (NAP) in Japan set a goal of achieving carbapenem resistance of *P. aeruginosa* of 3% or less by 2027 rather than focusing on the occurrence of MDRP [24]. Consequently, we also examined the current surveillance systems necessary at the national level for controlling drug-resistant *P. aeruginosa*.

Method

Study design and aims

We conducted a cross-sectional study to evaluate the NESID for MDRP infections through quantitative and qualitative methods in 2023. Our study aimed to evaluate whether NESID accurately captures the nationwide trend in MDRP infections and to examine the surveillance systems necessary for controlling drug-resistant *P. aeruginosa*. We assessed the nine attributes of representativeness, positive predictive value (PPV), sensitivity, data quality, acceptability, timeliness, simplicity, flexibility, and stability, and discussed the usefulness of this surveillance (**Table 1**). This evaluation was mainly based on the Guidelines for Evaluating Public Health Surveillance Systems published by the U.S. CDC in 2001 [25].

Table 1. Quantitative and qualitative evaluation for attributes of the surveillance system for MDRP infections in Japan

Attributes	Perspectives	Rationales	Results
Quantitative evaluation			
Representativeness	Are TDSSs selected without bias based on region?	The percentage of TDSSs among all medical institutions and the percentage of TDSSs with over 300 or more beds among all TDSSs were similar across prefectures (**Fig 1**).	Yes
PPV and Sensitivity	Do the trend in the number of reports to NESID as MDRP infections represent the trend in the number of accurate MDRP infections at TDSSs?	From 2018 to 2022, both the number of reported cases as MDRP infections and the accurate number of MDRP infections decreased over time (**Fig 2**).	Yes
PPV	Is the percentage of accurate MDRP infections among cases reported to NESID as MDRP infections at TDSSs high enough to be utilized?	PPV at subjected sentinel sites was 0.44, with 0.19 at sites with fewer than 300 beds and 0.48 at sites with 300 or more beds (**Table 3**).	No
Sensitivity	Is the percentage of reported cases to NESID among accurate MDRP infections at TDSSs high enough to be utilized?	Sensitivity at TDSSs was 0.65, with 1.00 at sites with fewer than 300 beds and 0.64 at sites with 300 or more beds (**Table 3**).	Yes and No
Data quality	Is the data recorded in the surveillance system complete and valid?	For 1,670 cases reported to NESID as MDRP infections from 2013 to 2022, all required items (age, sex, and detected specimen) were registered.	Yes
Qualitative evaluation			
Representativeness	(i) Does each prefecture select DSSs appropriately based on criteria?	In the subjected prefecture, nine DSSs were distributed evenly across the region. All DSSs offer both internal medicine and surgery, and eight had 300 and more beds.	Yes
	(ii) Are DSSs sufficient for reporting MDRP infections?	Reporting solely from DSSs would be inadequate for monitoring the outbreaks of MDRP infections because outbreaks occurring at long-term care facilities and small medical facilities would be overlooked.	No
Acceptability	Do the DSSs accept reporting of cases monthly?	It was easy to report MDRP infections once a month. However, physicians questioned whether monthly reports of MDRP infections, which require an outbreak response, were sufficient.	No
Timeliness	Is information promptly provided to the	As MDRP infections are reported once a month, prompt reports after	Not

	public after a physician diagnoses a patient?	diagnosis and feedback to the public are not required.	applicable
Simplicity	(i) Is it easy to report and confirm a case by using the system?	It was easy for physicians to report cases to NESID and information flow is simple, but staff at local infectious disease information centers found it difficult to confirm the accuracy of these reports.	Yes and No
	(ii) Is the case definition easy to understand?	The case definition is complicated due to the need to confirm resistance to three drugs using standards different from the breakpoints of CLSI and EUCAST, as well as the need to exclude colonized cases.	No
PPV	Does the system effectively prevent incorrect or missing reports?	There was a possibility of reporting errors caused by complicated case definition.	No
Flexibility	(i) Can the case definition be changed easily?	The case definition is strictly governed by Japanese law, requiring the approval of numerous experts and authorities based on clear evidence to be changed.	No
	(ii) Can DSSs add or change the reporting contents freely?	The reporting contents are also strictly governed by Japanese law.	No
	(iii) Can the prefecture change the DSSs easily?	The governor has the authority to change DSSs, but these sites have remained unchanged for at least 10 years in the subjected prefecture.	Yes and No
Stability	Can the surveillance system continue to function after unexpected accidents?	It is rare for the surveillance system's computers to experience unscheduled outages and downtime. In addition, this system continues to function unless directly affected by large-scale disasters such as the Japan Earthquake and Tsunami in 2011 and the Noto Earthquake in 2024.	Yes
Usefulness	Do government and medical institutions utilize the system to combat AMR?	The system makes it difficult for us to detect and respond to a cluster of MDRP infections at facilities other than DSSs. Additionally, the National Action Plan in Japan is aimed at reducing CRPA infections, not MDRP infections, and comparison of the situation with other developed countries would be difficult due to different definitions of MDRP.	No

AMR antimicrobial resistance, *CLSI* Clinical and Laboratory Standards Institute, *DSS* designated sentinel site, *CRPA* carbapenem-resistant *Pseudomonas aeruginosa*, *EUCAST* European Committee on Antimicrobial Susceptibility Testing, *MDRP* multidrug-resistant *Pseudomonas aeruginosa*, *NESID* National Epidemiological Surveillance of Infectious Diseases, *PPV* positive predictive value, *TDSS* target designated sentinel site

This evaluation was based on the Guidelines for Evaluating Public Health Surveillance Systems published by the Centers for Disease Control and Prevention in 2001 [23]

NESID for MDRP infections in Japan

In Japan, each prefectural government selects at least one designated sentinel site (DSS) per secondary medical area, which is a medical administrative area under the Medical Care Law. The DSS generally is capable of hospitalizing at least 300 patients and has internal medicine and surgery departments [16, 17]. The DSSs can be changed according to the local governments' needs. As details of the DSSs are not publicly available, we defined them as target DSSs (TDSSs) that remained open as of December 2022 and met either of the following two conditions:

(a) DSSs that reported MDRP infections to NESID from January 2013 to December 2022, or

(b) DSSs that reported methicillin-resistant *Staphylococcus aureus* (MRSA) infections to NESID from January to December 2021.

Condition (b) was required because MRSA infections are reported from most of the hospitals in Japan [19].

Data collection

Quantitative evaluation

We analyzed the characteristics of reports as MDRP infections to NESID from DSSs between January 2013 and December 2022. We accessed the data for research purposes on January 11, 2023. In February 2023, we sent a questionnaire ([illegible]**1 Appendix**) to TDSSs, requesting the accurate number of MDRP infections occurring at each site between January 2018 and December 2022. If responses from the TDSS included the answer "unknown" for any year, we excluded all responses from the TDSS as invalid responses. We evaluated these results separately for hospitals with 300 or

more beds and hospitals with fewer than 300 beds.

Qualitative evaluation

On the basis of the information flow (**S1 Fig**), we conducted face-to-face interviews with stakeholders of the surveillance system between 2 December 2022 and 24 August 2023 about its relevant attributes such as representativeness, acceptability, simplicity, and usefulness. In addition to three representatives from national academic authorities, our interviewees included two public health officers working at one local infectious disease surveillance center in a specific prefecture. We also selected six physicians, one infection control nurse, two pharmacists, and two bacteriology technologists working at five hospitals within the same prefecture (two TDSSs and three non-TDSSs), which were recommended by each local government. A well-trained interviewer conducted each interview by using a set of questionnaire items (**S2 Appendix**) that was reviewed by experienced physicians and clinical microbiology technologists.

Analysis

We defined (i) as the number of reports of MDRP infections to NESID from TDSSs between January 2018 and December 2022 and (ii) as the number of MDRP infections reported as accurate MDRP infections on the questionnaire survey. We defined (iii) as the number of cases misreported as MDRP infections despite not meeting the criteria and (iv) as the number of underreported accurate MDRP infections computed as follows (**S2 Fig**):

(iii) = (i) – (ii) ((i) > (ii))

(iv) = (ii) – (i) ((ii) > (i))

We counted (i) through (iv) for each hospital, aggregated them annually, and evaluated the trend. In addition, we calculated the PPV and sensitivity of reports of MDRP infections by using pooled data in NESID from 2018 to 2022.

Ethics considerations

Because the information handled in this study does not lead to the identification of individuals, the National Institute of Infectious Diseases did not require informed consent or ethical review (receipt no. 1478).

Results

In total, 1,670 cases were reported to NESID as MDRP infections from 347 DSSs between 2013 and 2022. One DSS reporting two cases closed in 2015, whereas the status of two others reporting one case each remained unknown. Although 119 DSSs did not report any cases as MDRP infections during the same period, they reported at least one case of MRSA infection in 2022. Thus, TDSSs in this study were defined as 463 facilities reporting 1,666 cases.

All 463 of the TDSSs offer both internal medicine and surgery. Among them, 347 (74.9%) have 300 or more beds and 116 (25.1%) have fewer than 300 beds, accounting for 24% and 2%, respectively, of all medical institutions with the same capacities in Japan. From 2013 to 2022, at least one case was reported as a MDRP infection by 262 (75.5%) of the 347 TDSSs with 300 or more beds and 82 (70.7%) of 116 TDSSs with

fewer than 300 beds. The results of the quantitative and qualitative evaluation for each attribute are summarized in **Table 1**.

Quantitative evaluation

TDSSs accounted for 5–10% of all medical institutions in many prefectures, and TDSSs with 300 or more beds accounted for more than 80% of all TDSSs in most prefectures (**Fig 1**). In the questionnaire survey, we obtained valid responses from 229 TDSSs (49.5%) (**Table 2**). From 2018 to 2022, these TDSSs reported 275 cases as MDRP infections, of which 182 were accurate MDRP infections, with both numbers decreasing over time (**Fig 2**). There were 162 misreported as MDRP infections despite not meeting the criteria and 69 underreported as MDRP infections, with **Fig 3** showing the distribution of TDSSs that misreported and underreported MDRP infections. Two TDSSs with fewer than 300 beds misreported 10 and 11 cases, respectively, and one TDSS with 300 or more beds underreported seven accurate MDRP infections. We excluded these cases as outliers when calculating PPV and sensitivity. Thus, the PPV and sensitivity for the reports of MDRP infections to NESID between 2018 and 2022 were 0.44 and 0.65, respectively (**Table 3**).

Fig 1 Distribution of 463 TDSSs. A. The percentage of TDSSs among all hospitals in each prefecture. B. The percentage of hospitals with 300 beds or more among the TDSSs in each prefecture. *TDSS* target designated sentinel site

Table 2. Distribution of subjected designated sentinel sites that responded to the questionnaire

	Number of TDSSs	TDSSs responding to the questionnaire n, (%)		TDSSs responding appropriately to the questionnaire n, (%)	
All TDSSs	463	232	(50.1)	229	(49.4)
TDSSs with 300 or more beds	347*	189	(54.5)	186	(53.6)
TDSSs with fewer than 300 beds	116†	43	(37.1)	43	(37.1)

TDSS subjected designated sentinel site

*Accounts for 24% of all institutions in Japan with 300 or more beds

†Accounts for 2% of all institutions in Japan with fewer than 300 beds

Fig 2. Trend in the number of cases reported as MDRP infections and the number of accurate MDRP infections at TDSSs, 2018–2022. From 2018 to 2022, these TDSSs reported 275 cases as MDRP infections, of which 182 were accurate MDRP infections, with both numbers decreasing over time. *NESID* National Epidemiological Surveillance of Infectious Diseases, *MDRP* Multidrug-resistant *Pseudomonas aeruginosa*, *TDSS* target designated sentinel site

Fig 3. Distribution of TDSSs that misreported cases as MDRP infections and underreported accurate MDRP infections, 2018-2022. A. Seventy-one TDSSs misreported a total of 162 cases as MDRP infections despite not meeting the criteria. B. Thirty-one TDSSs underreported a total of 69 accurate MDRP infections. *MDRP* Multidrug-resistant *Pseudomonas aeruginosa*, *TDSS* target designated sentinel site

Table 3. Positive predictive value and sensitivity for reports of MDRP infections between 2018 and 2022

	Positive predictive value	Sensitivity
Notification from all TDSSs	0.44	0.65
Notification from TDSSs with 300 or more beds	0.48	0.64
Notification from TDSSs with fewer than 300 beds	0.19	1.00

MDRP multidrug-resistant *Pseudomonas aeruginosa*, *TDSS* subjected designated sentinel site

Qualitative evaluation

One public health officer working at a local infectious disease surveillance center said that DSSs were selected properly based on Japanese law. However, this same public health officer, some physicians, and one infection control nurse pointed out that it was insufficient for only DSSs to report MDRP infections because outbreaks occurring at long-term care facilities or small hospitals may be overlooked. Additionally, they questioned the appropriateness of reporting MDRP infections monthly because such outbreaks require prompt public health action. Some physicians found it easy to report the cases to NESID, but these physicians and clinical microbiology technologists mentioned that reporting errors may be caused by complicated criteria used to confirm resistance to three antibiotics using standards different from the breakpoints of CLSI and EUCAST, and colonized cases were excluded. These physicians and national academic authorities indicated that the reports of CRPA infections would enable comparison of the situation with other countries and could be utilized as an indicator for the appropriate usage of broad-spectrum antibiotics based on the Japanese NAP. For these reasons, most of the interviewees concluded that the current surveillance system for MDRP infections is difficult for the government and medical institutions to utilize for the control of drug-resistant *P. aeruginosa*.

Discussion

We evaluated the Japanese surveillance system, NESID, for surveillance of MDRP infections quantitatively and qualitatively mainly based on the CDC guidelines [25]. Although current surveillance partially captured the trend of MDRP infections in

Japan, the surveillance should be designed based on actions and goals for the control of drug-resistant *P. aeruginosa* infections in the country.

The number of accurate MDRP infections occurring at TDSSs decreased over time, as did the number of cases reported as MDRP infections. Moreover, NESID showed good representativeness because TDSSs uniformly existed in each prefecture according to the population [16, 17], and there was no geographical bias detected in our findings (**Fig 1**). Thus, we considered that the decreasing trend in surveillance data in TDSSs reflected the actual trend of MDRP infections across Japan. Additionally, data from JANIS, another national-level AMR surveillance system in Japan, also showed a decreasing trend of MDRP detection in inpatient settings from 0.05% in 2017 to 0.03% in 2022, which may also reflect the actual domestic situation due to its high coverage of hospitals (close to one-fourth of all hospitals participated in 2022) in Japan [15, 19]. Consequently, the original objective of NESID to assess the trend of MDRP infections has been achieved, and we confirmed from the surveillance data that the number of MDRP infections in Japan has been decreasing.

We learned through the interviews that some of the interviewees were of the opinion that the current surveillance system does not meet public health needs. Thus, the current situation should be reviewed to improve the surveillance system to meet new objectives in the control of drug-resistant *P. aeruginosa* infections nationwide. Drug-resistant *P. aeruginosa* was classified into CRPA, MDRP, extensively drug-resistant *P. aeruginosa* (XDRP), and pan drug-resistant *P. aeruginosa* (PDRP) according to the standardized international terminology published in 2012 [26]. Furthermore, difficult-to-treat resistance *P. aeruginosa* (DTRP) was defined in 2018, with a focus on the lack of effective first-line drugs [27-29]. As drug-resistant *P. aeruginosa* has been classified

in several ways, each country focuses on different types [30]. In the AMR NAPs published in 132 countries [31], 51 countries (38.6%) mentioned *P. aeruginosa*, and 10 countries (7.6%) included it in their numerical benchmarks; eight countries targeted CRPA, while two countries targeted MDRP. No NAPs were targeting XDRP, PDRP, or DTRP at the end of 2023 (**S3 Table**).

The important points to be discussed in designing a surveillance system for drug-resistant *P. aeruginosa* are the definition of drug resistance including antimicrobial susceptibility testing and whether to conduct notifiable diseases surveillance or sentinel surveillance (**Table 4**). Regarding the definition of drug resistance, we focused on MDRP and CRPA, which were targeted by NAPs published in several countries.

Table 4. Characteristics of surveillance systems and target of national action plans for drug-resistant *Pseudomonas aeruginosa*

**Target drug-resistant *P. aeruginosa***		**Purpose**	**Advantages**	**Disadvantages**	**Countries including target benchmark for resistance of *P. aeruginosa* in each NAP [30]**
MDRP	Sentinel surveillance	・To evaluate the trend in the number of reported MDRP infections from sentinel sites	・Higher concern from the public than for CRPA	・Requires confirmation of resistance to three anti-pseudomonas antibiotics, which may lead to reporting errors<br>・Difficulty in detecting outbreaks at non-sentinel sites<br>・Difficulty in comparing the situation of AMR with other countries due to different definitions of MDRP	Philippines, Republic of Korea
	Notifiable disease surveillance	・To detect outbreaks to strengthen infection control measures<br>・To evaluate the trend in the number of notified MDRP infections from all facilities	・Higher concern from the public than for CRPA<br>・Ability to detect outbreaks at all facilities including non-sentinel sites	・Requires confirmation of resistance to three anti-pseudomonas antibiotics, which may lead to reporting errors<br>・Difficulty in comparing the situation of AMR with other countries due to different definitions of MDRP	None
CRPA	Sentinel surveillance	・To evaluate the trend in the number of reported CRPA infections from sentinel sites	・Confirmation of resistance to only carbapenems allows for a relatively straightforward determination<br>・Ability to easily compare the situation of AMR with other countries<br>・Ability to focus on and evaluate carbapenemase<br>・More countries set benchmarks in their NAP than for MDRP	・Difficulty in detecting outbreaks at non-sentinel sites<br>・Burden on medical institutions due to increase in the number of reports more than for MDRP	The United States, Greece, Poland, The United Kingdom of Great Britain and Northern Ireland, Thailand, Japan

Notifiable disease surveillance	・To detect outbreaks to strengthen infection control measures<br>・To evaluate the trend in the number of notified CRPA infections from all facilities	・Confirmation of resistance to only carbapenems allows for a relatively straightforward determination<br>・Ability to detect outbreaks in all facilities including non-sentinel sites<br>・Ability to easily compare the situation of AMR with other countries<br>・Ability to focus on and evaluate carbapenemase<br>・More countries set benchmarks in their NAP than for MDRP	・Severe burden on medical institutions due to excessive increase in number of notification	None

AMR antimicrobial resistance, *CRPA* carbapenem-resistant *P. aeruginosa*, *MDRP* multidrug-resistant *Pseudomonas aeruginosa*, *NAP* national action plan

Surveillance for drug-resistant *P. aeruginosa* to eliminate MDRP

MDRP is defined as *P. aeruginosa* non-susceptible to at least one agent in three and over antimicrobial categories such as carbapenems [26]. MDRP has quite limited treatment options and often leads to high inpatient mortality and prolonged hospital stays [32]. Thus, early detection is essential for an effective response to MDRP outbreaks. However, not all MDRP outbreaks can be identified through the sentinel surveillance unless the outbreak is epidemiologically or genetically unusual [33]. To eliminate MDRP outbreaks promptly, notifiable disease surveillance is preferable to sentinel surveillance because notifiable disease surveillance can detect MDRP infections from all hospitals. One challenge of the notifiable disease surveillance of MDRP is the difficulty in confirming drug resistance. Our results revealed an increase in the number of cases reported as MDRP infections despite not meeting the reporting criteria from 2018 to 2022. This issue would persist even if the surveillance system were changed to notifiable disease surveillance. Thus, quality assurance of antimicrobial susceptibility testing is the key to the successful implementation of the notification system. Another challenge is that we need to note whether the definition of MDRP is consistent when we compare the proportions of MDRP with other countries and regions.

Surveillance for drug-resistant *P. aeruginosa* to control carbapenem resistance

Carbapenems are essential broad-spectrum antibiotics that lead many developed

countries to monitor CRPA infections. Such monitoring enables these countries to compare the carbapenem resistance rates of *P. aeruginosa* with each other [34]. For instance, EARS-Net determined the proportions of CRPA in each country within the WHO European Region and showed large differences [12]. A previous article indicated that appropriate use of carbapenems was inversely correlated with the prevalence of CRPA [35]; thus, the proportions of CRPA could serve as an indicator of the rational use of carbapenems. In addition, as carbapenemase-producing isolates of *P. aeruginosa* can cause severe outbreaks [3, 36], laboratory testing for carbapenemase is crucial. In the U. S., the AR Lab Network reported detecting carbapenemase genes in 2.5% of 85,983 CRPA isolates analyzed between 2017 and 2023, with 61.0% of them identified as VIM [14]. In contrast, only 4.2% of 382 meropenem-resistant CRPA isolates collected from 78 hospitals in Japan possessed acquired carbapenemases, with the IMP type being the most frequent, and no VIM-producing CRPA isolates were detected. Multilocus sequence typing of the 382 CRPA isolates revealed that ST274 was predominant. Notably, ST274 CRPA isolates rarely carried carbapenemase [37]. We consider it important to integrate the reporting system with laboratory-based surveillance to comprehensively evaluate the trend of CRPA infections, including the results of genomic sequencing. However, if CRPA infections are classified as notifiable diseases, excessive numbers of cases would be reported, potentially leading to economic costs and burdens for medical and laboratory institutions because *P. aeruginosa* has resistance to carbapenems through various mechanisms other than carbapenemase production [4]. Thus, sentinel surveillance integrated with laboratory-based surveillance would be indispensable to controlling carbapenem resistance by assessment of the trend of the number of CRPA infections and evaluation of whether CRPA isolates produce any of the

carbapenemases.

Our study has some limitations. [illegible]st, as we interviewed staff members working at hospitals and one local infectious disease surveillance center located in a specific prefecture, it is possible that the situation might differ somewhat from those in other regions. However, we endeavored to generalize the findings by conducting interviews with national academic authorities from various prefectures. Second, the interview results could be influenced both by social desirability bias that interviewees' facilities have fewer underreported or misreported cases and by recall bias. Thus, we performed not only qualitative but also quantitative analysis for a comprehensive evaluation. Third, the reports of MDRP infections in NESID and our questionnaire survey were not confirmed by laboratory testing.

Conclusion

Our evaluation of the surveillance system for MDRP infections in Japan indicated that the reports of MDRP infections from DSSs represented the nationwide trend in MDRP infections, which has been decreasing over time. However, our study also showed that this system did not seem to meet current public health needs. Of concern were the difficulties in timely detection of and response to a cluster of MDRP infections due to reporting at monthly intervals, in accurate reporting due to complex diagnostic criteria, and in international comparison due to different definitions. As the epidemiology of drug-resistant *P. aeruginosa* is changing, the national policy may differ according to the public health needs, and the surveillance system also needs to be modified. Potential candidate systems may be a notifiable disease surveillance for MDRP infections or a sentinel surveillance integrated with laboratory-based

surveillance for CRPA infections. Based on our findings, we can contribute to develop the refined surveillance system to better manage drug resistance in *P. aeruginosa*.

Abbreviations

AMR	Antimicrobial resistance
AR Lab Network	Antibiotic Resistance Laboratory Network
CDC	Centers for Disease Control and Prevention
CLSI	Clinical and Laboratory Standards Institute
CRPA	Carbapenem-resistant *Pseudomonas aeruginosa*
DSS	Designated sentinel site
DTRP	Difficult-to-treat resistant *Pseudomonas aeruginosa*
EARS-Net	European Antimicrobial Resistance Surveillance Network
EUCAST	European Committee on Antimicrobial Susceptibility Testing
JANIS	Japan Nosocomial Infections Surveillance
MDRP	Multidrug-resistant *Pseudomonas aeruginosa*
MRSA	Methicillin-resistant *Staphylococcus aureus*
NESID	National Epidemiological Surveillance of Infectious Diseases
NAP	National action plan
PDRP	Pan drug-resistant *Pseudomonas aeruginosa*
PPV	Positive predictive value
TDSS	Target designated sentinel site
U.S.	United States
WHO	World Health Organization
XDRP	Extensively drug-resistant *Pseudomonas aeruginosa*

Declarations

Ethical approval and consent to participate

Because the information handled in this study does not lead to the identification of individuals, the National Institute of Infectious Diseases did not require informed consent or ethical review (receipt no. 1478).

Consent for publication

Not applicable.

Availability of data and materials

The datasets used and/or analyzed during the current study are available from the corresponding author on reasonable request.

Competing interests

The authors declare no competing interests.

Funding

This research was supported by the Ministry of Health, Labour and Welfare, Japan [Grant number 23HA1004].

Authors' contributions

SO and TY contributed to conception and design of the study. SO and TY collected the study data. SO, TY, T Shiomoto, MN, H Kurosu, CI, H Kato, MF, T Shimada, TT, M Suzuki, T Sunagawa, and M Sugai interpreted the data. SO and TY wrote the first draft of the manuscript, and T Shiomoto, TK, YA, and KN revised it. All authors reviewed the manuscript, made comments, and approved the final version.

Acknowledgements

We are grateful to public health officers, physicians, pharmacists, and clinical microbiology technologists who participated in the face-to-face interviews. We also thank the staff working at the designated sentinel sites, local public health centers, local infectious disease surveillance centers, and local public health institutes for cooperating with reporting to the national surveillance system and for responding to our questionnaire survey.

References

1. Kang CI, Kim SH, Kim H Bin, Park SW, Choe YJ, Oh MD, et al. Pseudomonas aeruginosa bacteremia: risk factors for mortality and influence of delayed receipt of effective antimicrobial therapy on clinical outcome. Clin Infect Dis. 2003;37:745–751.
2. Nathwani D, Raman G, Sulham K, Gavaghan M, Menon V. Clinical and economic consequences of hospital-acquired resistant and multidrug-resistant *Pseudomonas aeruginosa* infections: a systematic review and meta-analysis. Antimicrob Resist Infect Control. 2014;3:32. doi: 10.1186/2047-2994-3-32.

3. Grossman MK, Rankin DA, Maloney M, Stanton RA, Gable P, Stevens VA, et al. Extensively drug-resistant *Pseudomonas aeruginosa* outbreak associated with artificial tears. Clin Infect Dis. 2024;79:6-14.

4. Botelho J, Grosso F, Peixe L. Antibiotic resistance in *Pseudomonas aeruginosa* – mechanisms, epidemiology and evolution. Drug Resist Updat. 2019;44:100640. doi: 10.1016/j.drup.2019.07.002

5. Aloush V, Navon-Venezia S, Seigman-Igra Y, Cabili S, Carmeli Y. Multidrug-resistant *Pseudomonas aeruginosa*: risk factors and clinical impact. Antimicrob Agents Chemother. 2006;50:43–48.

6. Raman G, Avendano EE, Chan J, Merchant S, Puzniak L. Risk factors for hospitalized patients with resistant or multidrug-resistant *Pseudomonas aeruginosa* infections: a systematic review and meta-analysis. Antimicrob Resist Infect Control. 2018;7:1–14.

7. Cahill ME, Jaworski M, Harcy V, Young E, Ham DC, Gable P, et al. Cluster of carbapenemase-producing carbapenem-resistant *Pseudomonas aeruginosa* among patients in an adult intensive care unit – Idaho, 2021–2022. MMWR Morb Mortal Wkly Rep. 2023;72:844–846.

8. Zhang Y, Chen XL, Huang AW, Liu SL, Liu WJ, Zhang N, et al. Mortality attributable to carbapenem-resistant *Pseudomonas aeruginosa* bacteremia: a meta-analysis of cohort studies. Emerg Microbes Infect. 2016;5:e27. doi: 10.1038/emi.2016.22.

9. Tacconelli E, Carrara E, Savoldi A, Harbarth S, Mendelson M, Monnet DL, et al. Discovery, research, and development of new antibiotics: the WHO priority list of antibiotic-resistant bacteria and tuberculosis. Lancet Infect Dis. 2018;18:318–327.

10. World Health Organization. WHO Bacterial Priority Pathogens list, 2024. 2024. Available from: https://iris.who.int/bitstream/handle/10665/376776/9789240093461-eng.pdf?sequence=1 [Accessed 4 Jan 2025].

11. Centers for Disease Control and Prevention. Antibiotic Resistance Threats in the United States, 2019. 2019. Available from: https://ndc.services.cdc.gov/wp-content/uploads/Antibiotic-Resistance-Threats-in-the-United-States-2019.pdf [Accessed 4 Jan 2025].

12. WHO Regional Office for Europe/European Centre for Disease Prevention and Control. Antimicrobial resistance surveillance in Europe 2022 – 2020 data. 2022. Available from: https://www.ecdc.europa.eu/sites/default/files/documents/Joint-WHO-ECDC-AMR-report-2022.pdf [Accessed 4 Jan 2025].

13. Centers for Disease Control and Prevention. Antimicrobial Resistance Laboratory Network. 2024. Available from: https://www.cdc.gov/antimicrobial-resistance-laboratory-networks/php/about/domestic.html [Accessed 4 Jan 2025].

14. Centers for Disease Control and Prevention. Carbapenem-Resistant *Pseudomonas aeruginosa*. 2024. Available from: https://arpsp.cdc.gov/profile/arln/crpa?hidden= [Accessed 4 Jan 2025].

15. Suzuki S. A view on 20 years of antimicrobial resistance in Japan by two national surveillance systems: the National Epidemiological Surveillance of Infectious Diseases and Japan Nosocomial Infections Surveillance. Antibiotics. 2021;10:1189. doi: 10.3390/antibiotics10101189.

16. National Institution of Infectious Disease, Infectious Disease Surveillance Center. Infectious Disease Surveillance System in Japan. 2018. Available from: https://www.niid.go.jp/niid/images/epi/nesid/nesid_en.pdf [Accessed 4 Jan 2025].

17. Ministry of Health, Labour and Welfare. Implementation Manual for the National Epidemiological Surveillance of Infectious Diseases Program. 2019. Available from: https://www.mhlw.go.jp/content/10900000/000488981.pdf [Accessed 4 Jan 2025].

18. Kajihara T, Yahara K, Hirabayashi A, Shibayama K, Sugai M. Japan Nosocomial Infections Surveillance (JANIS): Current status, international collaboration, and future directions for a comprehensive antimicrobial resistance surveillance system. Jpn J Infect Dis. 2021;74:87–96.

19. Japan Nosocomial Infections Surveillance. Nosocomial infection control surveillance, laboratory department, inpatient specimens, 2022 (in Japanese). 2023. Available from: https://janis.mhlw.go.jp/report/open_report/2022/3/1/ken_Open_Report_202200.pdf [Accessed 19 May 2024].

20. Ministry of Health, Labour and Welfare. Multi-drug resistant *P. aeruginosa* (in Japanese). 2024. Available from: https://www.mhlw.go.jp/bunya/kenkou/kekkaku-kansenshou11/01-05-42-01.html [Accessed 19 May 2024].

21. Clinical and Laboratory Standards Institute. M100-ED34:2024 Performance Standards for Antimicrobial Susceptibility Testing, 34th Edition. 2024. Available from: http://em100.edaptivedocs.net/GetDoc.aspx?doc=CLSI%20M100%20ED34:2024&sbssok=CLSI%20M100%20ED34:2024%20TABLE%202B-1&format=HTML&hl=Pseudomonas [Accessed 25 Mar 2024].

22. European Committee on Antimicrobial Susceptibility Testing. Breakpoint tables for interpretation of MICs and zone diameters Version 14.0. 2024. Available from: https://www.eucast.org/fileadmin/src/media/PDFs/EUCAST_files/Breakpoint_tables/

v_14.0_Breakpoint_Tables.pdf [Accessed 25 Mar 2024].

23. National Institution of Infectious Disease. Notification of multi-drug resistant *P. aeruginosa* infections based on Japanese Law, 2022 (in Japanese). 2024. Available from: https://www.niid.go.jp/niid/ja/mdrp-m/mdrp-idwrs/12639-mdrp-240423.html [Accessed 4 Jan 2025].

24. The Government of Japan. National Action Plan on Antimicrobial Resistance (AMR) 2023-2027. 2023. Available from: https://www.mhlw.go.jp/content/10900000/001096228.pdf [Accessed 4 Jan 2025].

25. German RR, Lee LM, Horan JM, Milstein RL, Pertowski CA, Waller MN, et al. Updated guidelines for evaluating public health surveillance systems: recommendations from the Guidelines Working Group. MMWR Recomm Rep. 2001;50:1–35; quiz CE1-7.

26. Magiorakos AP, Srinivasan A, Carey RB, Carmeli Y, Falagas ME, Giske CG, et al. Multidrug-resistant, extensively drug-resistant and pandrug-resistant bacteria: an international expert proposal for interim standard definitions for acquired resistance. Clin Microbiol Infect. 2012;18:268–281.

27. Kadri SS, Adjemian J, Lai YL, Spaulding AB, Ricotta E, Prevots DR, et al. Difficult-to-treat resistance in Gram-negative bacteremia at 173 US hospitals: retrospective cohort analysis of prevalence, predictors, and outcome of resistance to all first-line agents. Clin Infect Dis. 2018;67:1803–1814.

28. Huh K, Chung DR, Ha YE, Ko JH, Kim SH, Kim MJ, et al. Impact of difficult-to-treat resistance in Gram-negative bacteremia on mortality: retrospective analysis of nationwide surveillance data. Clin Infect Dis. 2020;71:e487–496.

29. Tamma PD, Aitken SL, Bonomo RA, Mathers AJ, van Duin D, Clancy CJ.

Infectious Diseases Society of America 2023 Guidance on the Treatment of Antimicrobial Resistant Gram-Negative Infections. Clin Infect Dis. 2023:ciad428. doi: 10.1093/cid/ciad428.

30. Cosentino F, Viale P, Giannella M. MDR/XDR/PDR or DTR? Which definition best fits the resistance profile of *Pseudomonas aeruginosa*? Curr Opin Infect Dis. 2023;36:564–71.

31. World Health Organization. Library of AMR national action plans. 2023. Available from: https://www.who.int/teams/surveillance-prevention-control-AMR/national-action-plan-monitoring-evaluation/library-of-national-action-plans [Accessed 31 Dec 2023].

32. Barrasa-Villar JI, Aibar-Remón C, Prieto-Andrés P, Mareca-Doñate R, Moliner-Lahoz J. Impact on morbidity, mortality, and length of stay of hospital-acquired infections by resistant microorganisms. Clin Infect Dis. 2017;65:644–652.

33. Kanayama A, Kawahara R, Yamagishi T, Goto K, Kobaru Y, Takano M, et al. Successful control of an outbreak of GES-5 extended-spectrum β-lactamase-producing *Pseudomonas aeruginosa* in a long-term care facility in Japan. J Hosp Infect. 2016;93:35–41.

34. Reyes J, Komarow L, Chen L, Ge L, Hanson BM, Cober E, et al. Global epidemiology and clinical outcomes of carbapenem-resistant *Pseudomonas aeruginosa* and associated carbapenemases (POP): a prospective cohort study. Lancet Microbe. 2023;4:e159–170.

35. Zhang D, Cui K, Lu W, Bai H, Zhai Y, Hu S, et al. Evaluation of carbapenem use in a tertiary hospital: antimicrobial stewardship urgently needed. Antimicrob Resist Infect Control. 2019;8:5. doi: 10.1186/s13756-018-0449-3.

36. Catho G, Martischang R, Boroli F, Chraïti MN, Martin Y, Koyluk Tomsuk Z, et al. Outbreak of *Pseudomonas aeruginosa* producing VIM carbapenemase in an intensive care unit and its termination by implementation of waterless patient care. Crit Care. 2021;25:301. doi: 10.1186/s13054-021-03726-y.

37. Yano H, Hayashi W, Kawakami S, Aoki S, Anzai E, Zuo H, et al. Nationwide genome surveillance of carbapenem-resistant *Pseudomonas aeruginosa* in Japan. Antimicrob Agents Chemother. 2024;68:e0166923. doi: 10.1128/aac.01669-23.

Supporting Information

S1 Fig. Information flow of surveillance system for MDRP in Japan. The sentinel sites report the MDRP infections with the information of age, sex, and detected specimen to the local public health center monthly. The central infectious disease surveillance center integrates the data and shares it with the Ministry of Health, Labour and Welfare. *MDRP* multidrug-resistant *Pseudomonas aeruginosa*

S2 Fig. Theoretical schema of cases misreported as MDRP infections and underreported as accurate infections. (i) The number of cases reported as MDRP infections. (ii) The accurate number of MDRP infections. (iii) The number of cases misreported as MDRP infections. (iv) The number of underreported accurate MDRP infections. *MDRP* multidrug-resistant *Pseudomonas aeruginosa*

S1 Table. Criteria related to drug resistance in *Pseudomonas aeruginosa*

S2 Table. Difference of characteristics in drug-resistant *Pseudomonas aeruginosa* in each national action plan for antimicrobial resistance in various countries

S1 Appendix. Questionnaire on the Testing and Reporting System for Multidrug-resistant *Pseudomonas aeruginosa* (MDRP)

S2 Appendix. S2 Appendix. Interview form of surveillance evaluation for multi-drug resistant *Pseudomonas aeruginosa* (MDRP)

Click here to access/download;Figure;Figures.pdf

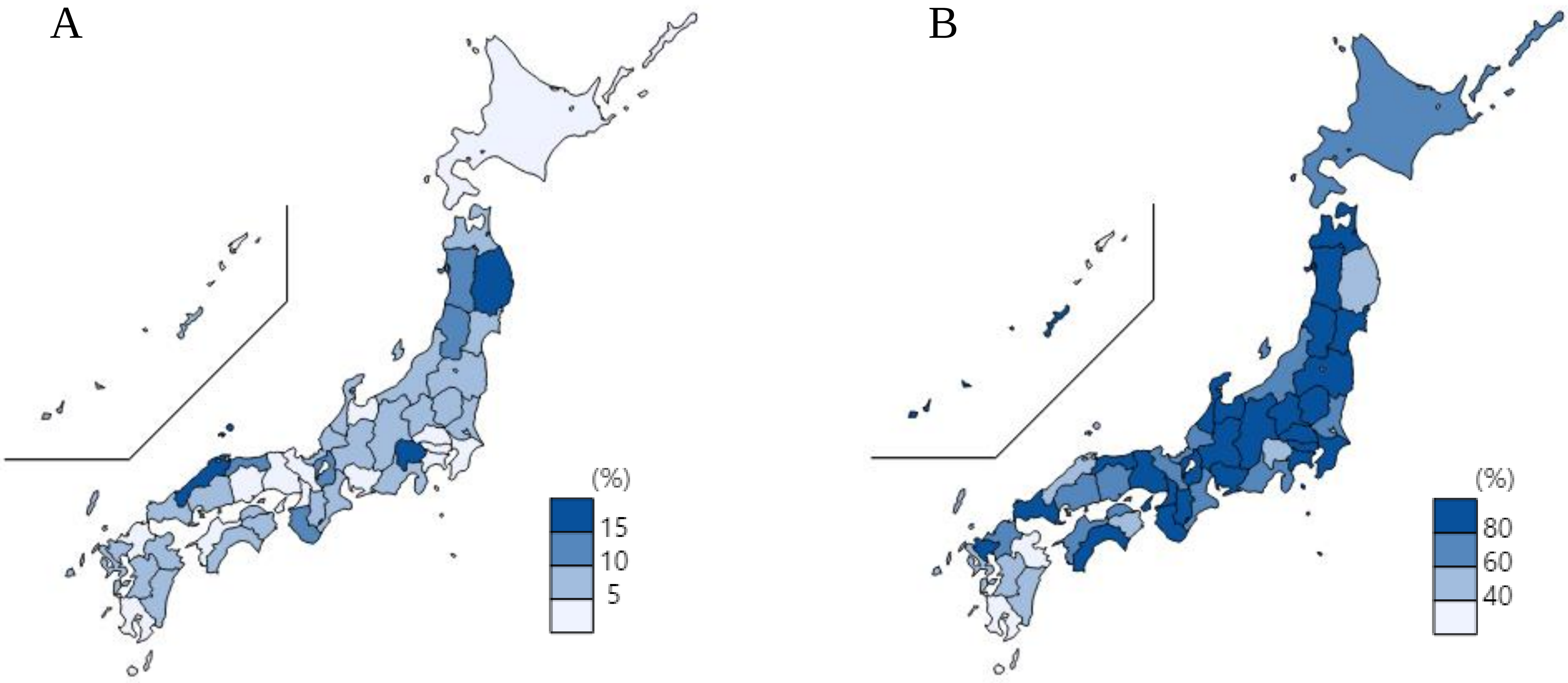

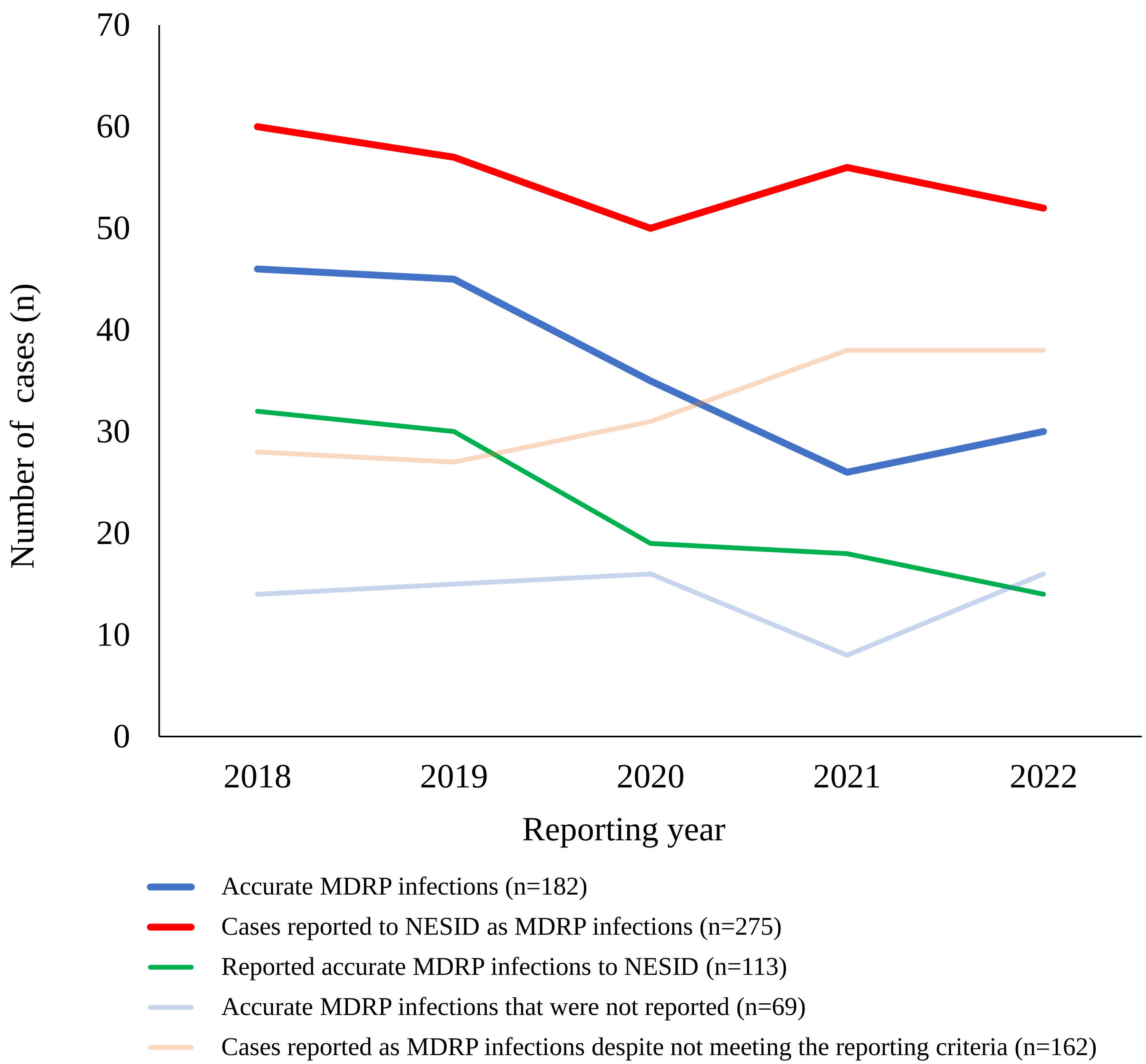
70
60
50
40
30
20
10
0
Number of cases (n)
2018
2019
2020
2021
2022
Reporting year
Accurate MDRP infections (n=182)
Cases reported to NESID as MDRP infections (n=275)
Reported accurate MDRP infections to NESID (n=113)
Accurate MDRP infections that were not reported (n=69)
Cases reported as MDRP infections despite not meeting the reporting criteria (n=162)

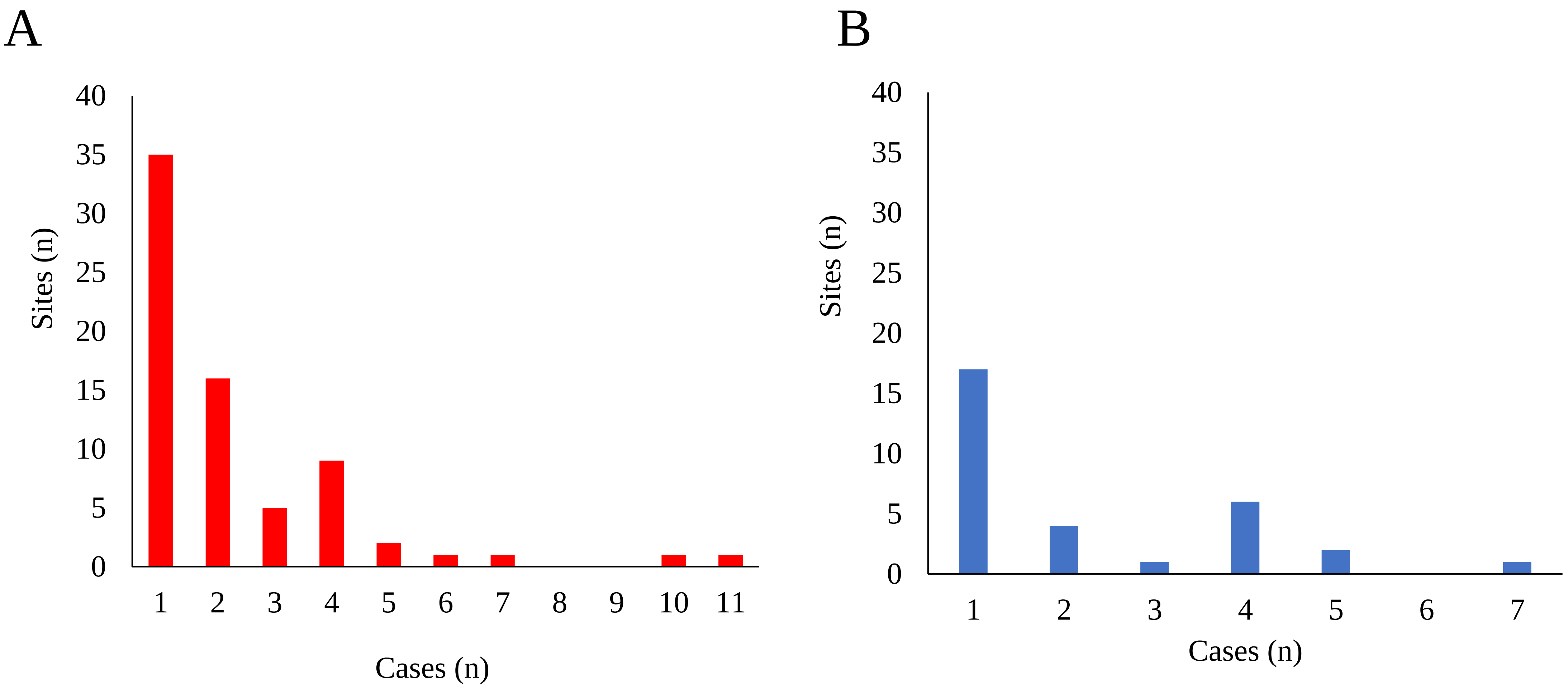
A
B
Sites (n)
Cases (n)
0
5
10
15
20
25
30
35
40
1
2
3
4
5
6
7
8
9
10
11

Click here to access/download;Figure;Supplementary Figures.pdf

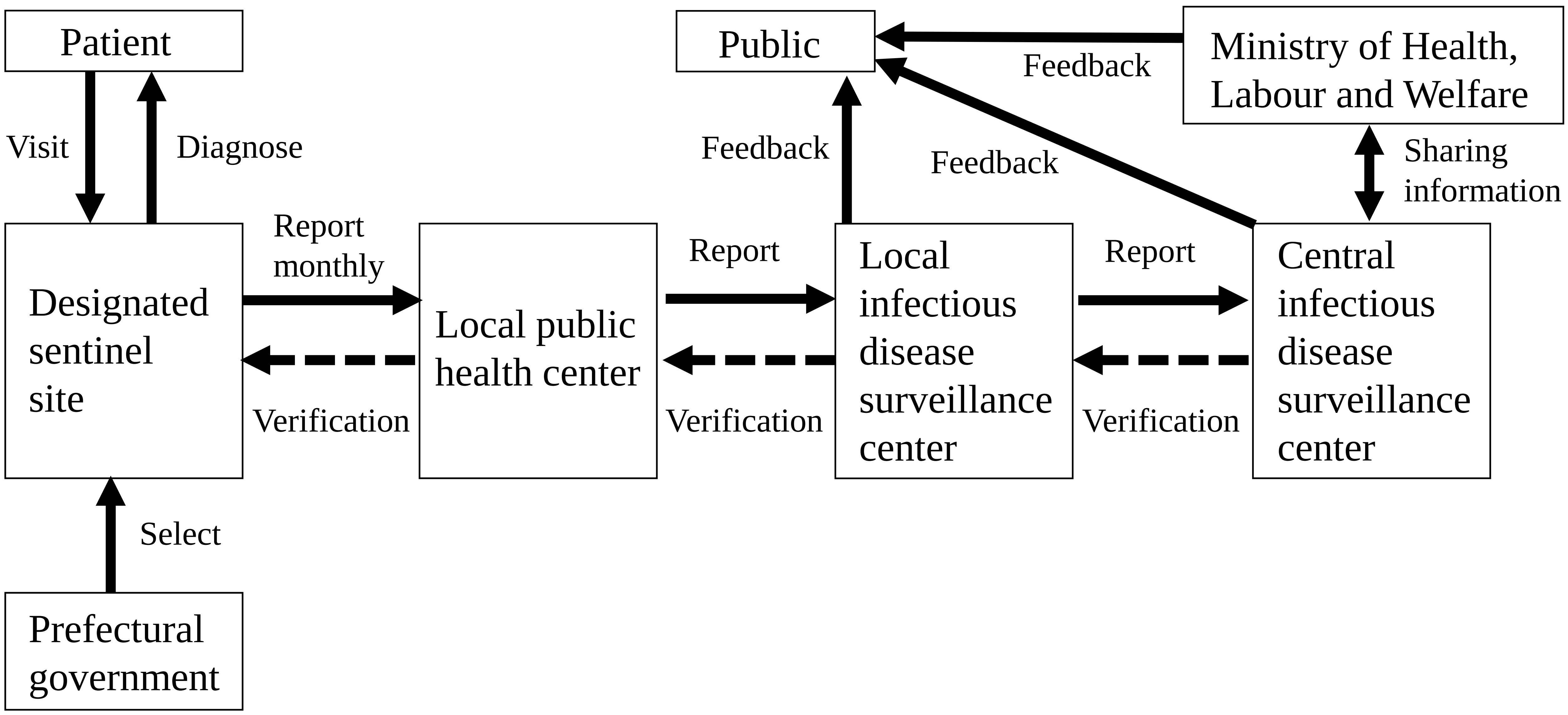

	(i)>(ii)	(i)<(ii)
Definition	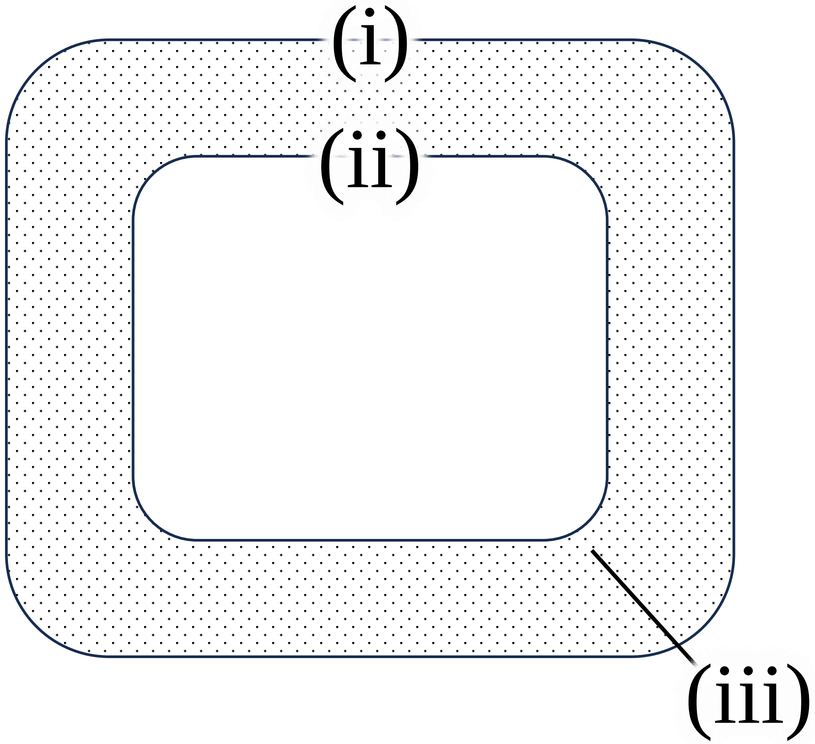	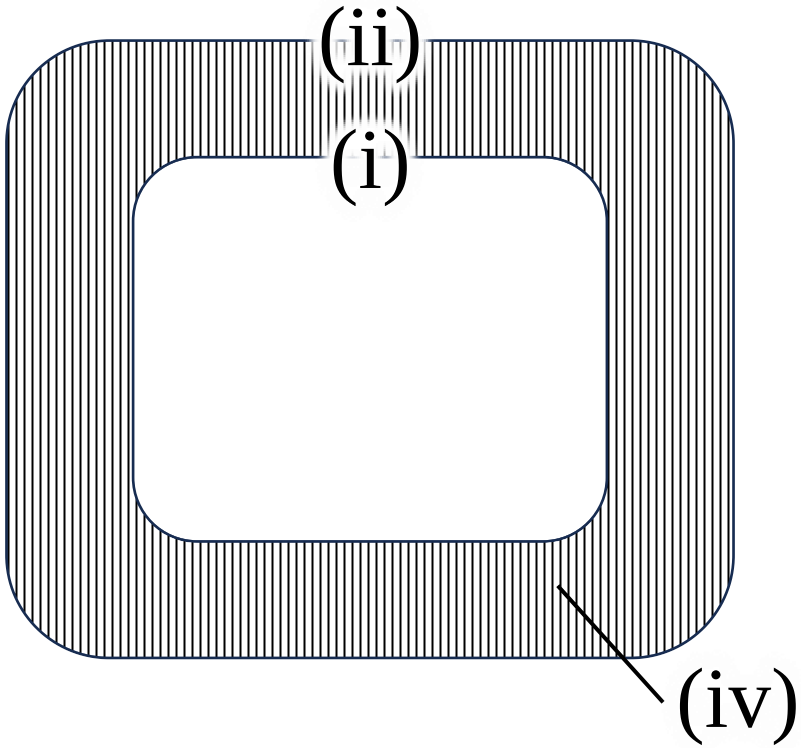
Positive predictive value	(ii)/(i)	1
Sensitivity	1	(i)/(ii)

Supplementary table 1

Click here to access/download
Supporting Information
S1 Table.xlsx

Supplementary table 2

Click here to access/download
Supporting Information
S2 Table.xlsx

Appendix 1

Click here to access/download
Supporting Information
S1 Appendix.docx

Appendix 2

Click here to access/download
Supporting Information
S2 Appendix.docx

strobe statement

Click here to access/download
Supporting Information
renamed_86635.doc
