# Supplementary material for: What we can and cannot see from the surveillance for drug-resistant Pseudomonas aeruginosa—Findings from the evaluation of a surveillance system for multidrug-resistant P. aeruginosa infections in Japan
Source: PLoS One. 2025 Aug 4;20(8):e0329635. doi: 10.1371/journal.pone.0329635 (PMC12321080; doi:10.1371/journal.pone.0329635)
Supplement: Attachment — Submitted filename: Response to editor and reviewers_20250510.docx [file pone.0329635.s013.docx]

**Response to the editorial comments**

1. Please ensure that your manuscript meets PLOS ONE's style requirements, including those for file naming. The PLOS ONE style templates can be found at <https://journals.plos.org/plosone/s/file?id=wjVg/PLOSOne_formatting_sample_main_body.pdf> and <https://journals.plos.org/plosone/s/file?id=ba62/PLOSOne_formatting_sample_title_authors_affiliations.pdf>

Response: Thank you for your comment. We have carefully checked the manuscript and revised it according to the journal requirements.

2. We note that the grant information you provided in the ‘Funding Information’ and ‘Financial Disclosure’ sections do not match. When you resubmit, please ensure that you provide the correct grant numbers for the awards you received for your study in the ‘Funding Information’ section.

Response: Thank you for noting this. Funding information on the site was “Ministry of Health, Labour and Welfare Award Number: 23HA1004 | Recipient: Takuya Yamagishi, Dr.” and the description in the body of the manuscript was “This research was supported by the Ministry of Health, Labour and Welfare, Japan [Grant number 23HA1004]”. It was the same. But if there is any discrepancy, could you tell us how to correct the information.

3. Thank you for stating the following financial disclosure: Grant from Ministry of Health, Labour, and Welfare, Japan
Please state what role the funders took in the study. If the funders had no role, please state: The funders had no role in study design, data collection and analysis, decision to publish, or preparation of the manuscript."
If this statement is not correct you must amend it as needed.
Please include this amended Role of Funder statement in your cover letter; we will change the online submission form on your behalf.

Response: Thank you for your comments. We have added the suggested statements in the “Funding” section on Lines 474-475.

4. Your ethics statement should only appear in the Methods section of your manuscript. If your ethics statement is written in any section besides the Methods, please delete it from any other section.

Response: Thank you for your suggestion, which we have followed.

5. We note that you have indicated that there are restrictions to data sharing for this study. For studies involving human research participant data or other sensitive data, we encourage authors to share de-identified or anonymized data. However, when data cannot be publicly shared for ethical reasons, we allow authors to make their data sets available upon request. For information on unacceptable data access restrictions, please see <http://journals.plos.org/plosone/s/data-availability#loc-unacceptable-data-access-restrictions>.
Before we proceed with your manuscript, please address the following prompts:

a) If there are ethical or legal restrictions on sharing a de-identified data set, please explain them in detail (e.g., data contain potentially identifying or sensitive patient information, data are owned by a third-party organization, etc.) and who has imposed them (e.g., a Research Ethics Committee or Institutional Review Board, etc.). Please also provide contact information for a data access committee, ethics committee, or other institutional body to which data requests may be sent.
b) If there are no restrictions, please upload the minimal anonymized data set necessary to replicate your study findings to a stable, public repository and provide us with the relevant URLs, DOIs, or accession numbers. Please see <http://www.bmj.com/content/340/bmj.c181.long> for guidelines on how to de-identify and prepare clinical data for publication. For a list of recommended repositories, please see <https://journals.plos.org/plosone/s/recommended-repositories>. You also have the option of uploading the data as Supporting Information files, but we would recommend depositing data directly to a data repository if possible.
Please update your Data Availability statement in the submission form accordingly.

Response: Thank you for your comments. We have provided a combined response to Comments 5 and 6 as outlined below.

6. In the online submission form, you indicated that data we collected can be shared publicly, but data on the National Epidemiological Surveillance of Infectious Diseases cannot be shared publicly because the data are strictly protected by the law and need for the governmental clearance for use.
All PLOS journals now require all data underlying the findings described in their manuscript to be freely available to other researchers, either 1. In a public repository, 2. Within the manuscript itself, or 3. Uploaded as supplementary information.
This policy applies to all data except where public deposition would breach compliance with the protocol approved by your research ethics board. If your data cannot be made publicly available for ethical or legal reasons (e.g., public availability would compromise patient privacy), please explain your reasons on resubmission and your exemption request will be escalated for approval.

Response: We are sorry for providing incorrect information. All the NESID data used in this study is open to the public with aggregation on the following sites:

- 2022: <https://id-info.jihs.go.jp/surveillance/idwr/annual/2022/index.html>
- Prior to 2022: <https://id-info.jihs.go.jp/surveillance/idwr/annual/2022/archive/index.html>

Please note that these data are subject to change due to delayed reports or withdrawals by local governments.

We stated this information on the online submission form and in the “Availability of data and materials” section in Lines 462-465.

7. [We note that Figure 1 in your submission contain [map/satellite] images which may be copyrighted. All PLOS content is published under the Creative Commons Attribution License (CC BY 4.0), which means that the manuscript, images, and Supporting Information files will be freely available online, and any third party is permitted to access, download, copy, distribute, and use these materials in any way, even commercially, with proper attribution. For these reasons, we cannot publish previously copyrighted maps or satellite images created using proprietary data, such as Google software (Google Maps, Street View, and Earth). For more information, see our copyright guidelines: http://journals.plos.org/plosone/s/licenses-and-copyright.](http://track.editorialmanager.com/CL0/We%2520note%2520that%2520Figure%25201%2520in%2520your%2520submission%2520contain%2520%255bmap%2Fsatellite%255d%2520images%2520which%2520may%2520be%2520copyrighted.%2520All%2520PLOS%2520content%2520is%2520published%2520under%2520the%2520Creative%2520Commons%2520Attribution%2520License%2520(CC%2520BY%25204.0),%2520which%2520means%2520that%2520the%2520manuscript,%2520images,%2520and%2520Supporting%2520Information%2520files%2520will%2520be%2520freely%2520available%2520online,%2520and%2520any%2520third%2520party%2520is%2520permitted%2520to%2520access,%2520download,%2520copy,%2520distribute,%2520and%2520use%2520these%2520materials%2520in%2520any%2520way,%2520even%2520commercially,%2520with%2520proper%2520attribution.%2520For%2520these%2520reasons,%2520we%2520cannot%2520publish%2520previously%2520copyrighted%2520maps%2520or%2520satellite%2520images%2520created%2520using%2520proprietary%2520data,%2520such%2520as%2520Google%2520software%2520(Google%2520Maps,%2520Street%2520View,%2520and%2520Earth).%2520For%2520more%2520information,%2520see%2520our%2520copyright%2520guidelines:%2520http:%2Fjournals.plos.org%2Fplosone%2Fs%2Flicenses-and-copyright.%250b%250bWe%2520require%2520you%2520to%2520either%2520(1)%2520present%2520written%2520permission%2520from%2520the%2520copyright%2520holder%2520to%2520publish%2520these%2520figures%2520specifically%2520under%2520the%2520CC%2520BY%25204.0%2520license,%2520or%2520(2)%2520remove%2520the%2520figures%2520from%2520your%2520submission:%250b%250ba.%2520You%2520may%2520seek%2520permission%2520from%2520the%2520original%2520copyright%2520holder%2520of%2520Figure%25201%2520to%2520publish%2520the%2520content%2520specifically%2520under%2520the%2520CC%2520BY%25204.0%2520license.%2520%250b%250bWe%2520recommend%2520that%2520you%2520contact%2520the%2520original%2520copyright%2520holder%2520with%2520the%2520Content%2520Permission%2520Form%2520(http:%2Fjournals.plos.org%2Fplosone%2Fs%2Ffile%3Fid=7c09%2Fcontent-permission-form.pdf)%2520and%2520the%2520following%2520text:%250b%E2%80%9CI%2520request%2520permission%2520for%2520the%2520open-access%2520journal%2520PLOS%2520ONE%2520to%2520publish%2520XXX%2520under%2520the%2520Creative%2520Commons%2520Attribution%2520License%2520(CCAL)%2520CC%2520BY%25204.0%2520(http:%2F%2Fcreativecommons.org%2Flicenses%2Fby%2F4.0%2F).%2520Please%2520be%2520aware%2520that%2520this%2520license%2520allows%2520unrestricted%2520use%2520and%2520distribution,%2520even%2520commercially,%2520by%2520third%2520parties.%2520Please%2520reply%2520and%2520provide%2520explicit%2520written%2520permission%2520to%2520publish%2520XXX%2520under%2520a%2520CC%2520BY%2520license%2520and%2520complete%2520the%2520attached%2520form.%E2%80%9D%250b%250bPlease%2520upload%2520the%2520completed%2520Content%2520Permission%2520Form%2520or%2520other%2520proof%2520of%2520granted%2520permissions%2520as%2520an%2520%2522Other%2522%2520file%2520with%2520your%2520submission.%250b%250bIn%2520the%2520figure%2520caption%2520of%2520the%2520copyrighted%2520figure,%2520please%2520include%2520the%2520following%2520text:%2520%E2%80%9CReprinted%2520from%2520%255bref%255d%2520under%2520a%2520CC%2520BY%2520license,%2520with%2520permission%2520from%2520%255bname%2520of%2520publisher%255d,%2520original%2520copyright%2520%255boriginal%2520copyright%2520year%255d.%E2%80%9D%250b%250bb.%2520If%2520you%2520are%2520unable%2520to%2520obtain%2520permiss/1/010f0195d4f70bf9-6543d57e-4a22-449b-8de4-f487457c29e1-000000/KQjRjZVLS913SYbFlnS7sIRF7FE8EpSESWorl1I1cbY=204)
[We require you to either (1) present written permission from the copyright holder to publish these figures specifically under the CC BY 4.0 license, or (2) remove the figures from your submission:](http://track.editorialmanager.com/CL0/We%2520note%2520that%2520Figure%25201%2520in%2520your%2520submission%2520contain%2520%255bmap%2Fsatellite%255d%2520images%2520which%2520may%2520be%2520copyrighted.%2520All%2520PLOS%2520content%2520is%2520published%2520under%2520the%2520Creative%2520Commons%2520Attribution%2520License%2520(CC%2520BY%25204.0),%2520which%2520means%2520that%2520the%2520manuscript,%2520images,%2520and%2520Supporting%2520Information%2520files%2520will%2520be%2520freely%2520available%2520online,%2520and%2520any%2520third%2520party%2520is%2520permitted%2520to%2520access,%2520download,%2520copy,%2520distribute,%2520and%2520use%2520these%2520materials%2520in%2520any%2520way,%2520even%2520commercially,%2520with%2520proper%2520attribution.%2520For%2520these%2520reasons,%2520we%2520cannot%2520publish%2520previously%2520copyrighted%2520maps%2520or%2520satellite%2520images%2520created%2520using%2520proprietary%2520data,%2520such%2520as%2520Google%2520software%2520(Google%2520Maps,%2520Street%2520View,%2520and%2520Earth).%2520For%2520more%2520information,%2520see%2520our%2520copyright%2520guidelines:%2520http:%2Fjournals.plos.org%2Fplosone%2Fs%2Flicenses-and-copyright.%250b%250bWe%2520require%2520you%2520to%2520either%2520(1)%2520present%2520written%2520permission%2520from%2520the%2520copyright%2520holder%2520to%2520publish%2520these%2520figures%2520specifically%2520under%2520the%2520CC%2520BY%25204.0%2520license,%2520or%2520(2)%2520remove%2520the%2520figures%2520from%2520your%2520submission:%250b%250ba.%2520You%2520may%2520seek%2520permission%2520from%2520the%2520original%2520copyright%2520holder%2520of%2520Figure%25201%2520to%2520publish%2520the%2520content%2520specifically%2520under%2520the%2520CC%2520BY%25204.0%2520license.%2520%250b%250bWe%2520recommend%2520that%2520you%2520contact%2520the%2520original%2520copyright%2520holder%2520with%2520the%2520Content%2520Permission%2520Form%2520(http:%2Fjournals.plos.org%2Fplosone%2Fs%2Ffile%3Fid=7c09%2Fcontent-permission-form.pdf)%2520and%2520the%2520following%2520text:%250b%E2%80%9CI%2520request%2520permission%2520for%2520the%2520open-access%2520journal%2520PLOS%2520ONE%2520to%2520publish%2520XXX%2520under%2520the%2520Creative%2520Commons%2520Attribution%2520License%2520(CCAL)%2520CC%2520BY%25204.0%2520(http:%2F%2Fcreativecommons.org%2Flicenses%2Fby%2F4.0%2F).%2520Please%2520be%2520aware%2520that%2520this%2520license%2520allows%2520unrestricted%2520use%2520and%2520distribution,%2520even%2520commercially,%2520by%2520third%2520parties.%2520Please%2520reply%2520and%2520provide%2520explicit%2520written%2520permission%2520to%2520publish%2520XXX%2520under%2520a%2520CC%2520BY%2520license%2520and%2520complete%2520the%2520attached%2520form.%E2%80%9D%250b%250bPlease%2520upload%2520the%2520completed%2520Content%2520Permission%2520Form%2520or%2520other%2520proof%2520of%2520granted%2520permissions%2520as%2520an%2520%2522Other%2522%2520file%2520with%2520your%2520submission.%250b%250bIn%2520the%2520figure%2520caption%2520of%2520the%2520copyrighted%2520figure,%2520please%2520include%2520the%2520following%2520text:%2520%E2%80%9CReprinted%2520from%2520%255bref%255d%2520under%2520a%2520CC%2520BY%2520license,%2520with%2520permission%2520from%2520%255bname%2520of%2520publisher%255d,%2520original%2520copyright%2520%255boriginal%2520copyright%2520year%255d.%E2%80%9D%250b%250bb.%2520If%2520you%2520are%2520unable%2520to%2520obtain%2520permiss/1/010f0195d4f70bf9-6543d57e-4a22-449b-8de4-f487457c29e1-000000/KQjRjZVLS913SYbFlnS7sIRF7FE8EpSESWorl1I1cbY=204)
[a. You may seek permission from the original copyright holder of Figure 1 to publish the content specifically under the CC BY 4.0 license.](http://track.editorialmanager.com/CL0/We%2520note%2520that%2520Figure%25201%2520in%2520your%2520submission%2520contain%2520%255bmap%2Fsatellite%255d%2520images%2520which%2520may%2520be%2520copyrighted.%2520All%2520PLOS%2520content%2520is%2520published%2520under%2520the%2520Creative%2520Commons%2520Attribution%2520License%2520(CC%2520BY%25204.0),%2520which%2520means%2520that%2520the%2520manuscript,%2520images,%2520and%2520Supporting%2520Information%2520files%2520will%2520be%2520freely%2520available%2520online,%2520and%2520any%2520third%2520party%2520is%2520permitted%2520to%2520access,%2520download,%2520copy,%2520distribute,%2520and%2520use%2520these%2520materials%2520in%2520any%2520way,%2520even%2520commercially,%2520with%2520proper%2520attribution.%2520For%2520these%2520reasons,%2520we%2520cannot%2520publish%2520previously%2520copyrighted%2520maps%2520or%2520satellite%2520images%2520created%2520using%2520proprietary%2520data,%2520such%2520as%2520Google%2520software%2520(Google%2520Maps,%2520Street%2520View,%2520and%2520Earth).%2520For%2520more%2520information,%2520see%2520our%2520copyright%2520guidelines:%2520http:%2Fjournals.plos.org%2Fplosone%2Fs%2Flicenses-and-copyright.%250b%250bWe%2520require%2520you%2520to%2520either%2520(1)%2520present%2520written%2520permission%2520from%2520the%2520copyright%2520holder%2520to%2520publish%2520these%2520figures%2520specifically%2520under%2520the%2520CC%2520BY%25204.0%2520license,%2520or%2520(2)%2520remove%2520the%2520figures%2520from%2520your%2520submission:%250b%250ba.%2520You%2520may%2520seek%2520permission%2520from%2520the%2520original%2520copyright%2520holder%2520of%2520Figure%25201%2520to%2520publish%2520the%2520content%2520specifically%2520under%2520the%2520CC%2520BY%25204.0%2520license.%2520%250b%250bWe%2520recommend%2520that%2520you%2520contact%2520the%2520original%2520copyright%2520holder%2520with%2520the%2520Content%2520Permission%2520Form%2520(http:%2Fjournals.plos.org%2Fplosone%2Fs%2Ffile%3Fid=7c09%2Fcontent-permission-form.pdf)%2520and%2520the%2520following%2520text:%250b%E2%80%9CI%2520request%2520permission%2520for%2520the%2520open-access%2520journal%2520PLOS%2520ONE%2520to%2520publish%2520XXX%2520under%2520the%2520Creative%2520Commons%2520Attribution%2520License%2520(CCAL)%2520CC%2520BY%25204.0%2520(http:%2F%2Fcreativecommons.org%2Flicenses%2Fby%2F4.0%2F).%2520Please%2520be%2520aware%2520that%2520this%2520license%2520allows%2520unrestricted%2520use%2520and%2520distribution,%2520even%2520commercially,%2520by%2520third%2520parties.%2520Please%2520reply%2520and%2520provide%2520explicit%2520written%2520permission%2520to%2520publish%2520XXX%2520under%2520a%2520CC%2520BY%2520license%2520and%2520complete%2520the%2520attached%2520form.%E2%80%9D%250b%250bPlease%2520upload%2520the%2520completed%2520Content%2520Permission%2520Form%2520or%2520other%2520proof%2520of%2520granted%2520permissions%2520as%2520an%2520%2522Other%2522%2520file%2520with%2520your%2520submission.%250b%250bIn%2520the%2520figure%2520caption%2520of%2520the%2520copyrighted%2520figure,%2520please%2520include%2520the%2520following%2520text:%2520%E2%80%9CReprinted%2520from%2520%255bref%255d%2520under%2520a%2520CC%2520BY%2520license,%2520with%2520permission%2520from%2520%255bname%2520of%2520publisher%255d,%2520original%2520copyright%2520%255boriginal%2520copyright%2520year%255d.%E2%80%9D%250b%250bb.%2520If%2520you%2520are%2520unable%2520to%2520obtain%2520permiss/1/010f0195d4f70bf9-6543d57e-4a22-449b-8de4-f487457c29e1-000000/KQjRjZVLS913SYbFlnS7sIRF7FE8EpSESWorl1I1cbY=204)
[We recommend that you contact the original copyright holder with the Content Permission Form (http://journals.plos.org/plosone/s/file?id=7c09/content-permission-form.pdf) and the following text:](http://track.editorialmanager.com/CL0/We%2520note%2520that%2520Figure%25201%2520in%2520your%2520submission%2520contain%2520%255bmap%2Fsatellite%255d%2520images%2520which%2520may%2520be%2520copyrighted.%2520All%2520PLOS%2520content%2520is%2520published%2520under%2520the%2520Creative%2520Commons%2520Attribution%2520License%2520(CC%2520BY%25204.0),%2520which%2520means%2520that%2520the%2520manuscript,%2520images,%2520and%2520Supporting%2520Information%2520files%2520will%2520be%2520freely%2520available%2520online,%2520and%2520any%2520third%2520party%2520is%2520permitted%2520to%2520access,%2520download,%2520copy,%2520distribute,%2520and%2520use%2520these%2520materials%2520in%2520any%2520way,%2520even%2520commercially,%2520with%2520proper%2520attribution.%2520For%2520these%2520reasons,%2520we%2520cannot%2520publish%2520previously%2520copyrighted%2520maps%2520or%2520satellite%2520images%2520created%2520using%2520proprietary%2520data,%2520such%2520as%2520Google%2520software%2520(Google%2520Maps,%2520Street%2520View,%2520and%2520Earth).%2520For%2520more%2520information,%2520see%2520our%2520copyright%2520guidelines:%2520http:%2Fjournals.plos.org%2Fplosone%2Fs%2Flicenses-and-copyright.%250b%250bWe%2520require%2520you%2520to%2520either%2520(1)%2520present%2520written%2520permission%2520from%2520the%2520copyright%2520holder%2520to%2520publish%2520these%2520figures%2520specifically%2520under%2520the%2520CC%2520BY%25204.0%2520license,%2520or%2520(2)%2520remove%2520the%2520figures%2520from%2520your%2520submission:%250b%250ba.%2520You%2520may%2520seek%2520permission%2520from%2520the%2520original%2520copyright%2520holder%2520of%2520Figure%25201%2520to%2520publish%2520the%2520content%2520specifically%2520under%2520the%2520CC%2520BY%25204.0%2520license.%2520%250b%250bWe%2520recommend%2520that%2520you%2520contact%2520the%2520original%2520copyright%2520holder%2520with%2520the%2520Content%2520Permission%2520Form%2520(http:%2Fjournals.plos.org%2Fplosone%2Fs%2Ffile%3Fid=7c09%2Fcontent-permission-form.pdf)%2520and%2520the%2520following%2520text:%250b%E2%80%9CI%2520request%2520permission%2520for%2520the%2520open-access%2520journal%2520PLOS%2520ONE%2520to%2520publish%2520XXX%2520under%2520the%2520Creative%2520Commons%2520Attribution%2520License%2520(CCAL)%2520CC%2520BY%25204.0%2520(http:%2F%2Fcreativecommons.org%2Flicenses%2Fby%2F4.0%2F).%2520Please%2520be%2520aware%2520that%2520this%2520license%2520allows%2520unrestricted%2520use%2520and%2520distribution,%2520even%2520commercially,%2520by%2520third%2520parties.%2520Please%2520reply%2520and%2520provide%2520explicit%2520written%2520permission%2520to%2520publish%2520XXX%2520under%2520a%2520CC%2520BY%2520license%2520and%2520complete%2520the%2520attached%2520form.%E2%80%9D%250b%250bPlease%2520upload%2520the%2520completed%2520Content%2520Permission%2520Form%2520or%2520other%2520proof%2520of%2520granted%2520permissions%2520as%2520an%2520%2522Other%2522%2520file%2520with%2520your%2520submission.%250b%250bIn%2520the%2520figure%2520caption%2520of%2520the%2520copyrighted%2520figure,%2520please%2520include%2520the%2520following%2520text:%2520%E2%80%9CReprinted%2520from%2520%255bref%255d%2520under%2520a%2520CC%2520BY%2520license,%2520with%2520permission%2520from%2520%255bname%2520of%2520publisher%255d,%2520original%2520copyright%2520%255boriginal%2520copyright%2520year%255d.%E2%80%9D%250b%250bb.%2520If%2520you%2520are%2520unable%2520to%2520obtain%2520permiss/1/010f0195d4f70bf9-6543d57e-4a22-449b-8de4-f487457c29e1-000000/KQjRjZVLS913SYbFlnS7sIRF7FE8EpSESWorl1I1cbY=204)
[“I request permission for the open-access journal PLOS ONE to publish XXX under the Creative Commons Attribution License (CCAL) CC BY 4.0 (http://creativecommons.org/licenses/by/4.0/). Please be aware that this license allows unrestricted use and distribution, even commercially, by third parties. Please reply and provide explicit written permission to publish XXX under a CC BY license and complete the attached form.”](http://track.editorialmanager.com/CL0/We%2520note%2520that%2520Figure%25201%2520in%2520your%2520submission%2520contain%2520%255bmap%2Fsatellite%255d%2520images%2520which%2520may%2520be%2520copyrighted.%2520All%2520PLOS%2520content%2520is%2520published%2520under%2520the%2520Creative%2520Commons%2520Attribution%2520License%2520(CC%2520BY%25204.0),%2520which%2520means%2520that%2520the%2520manuscript,%2520images,%2520and%2520Supporting%2520Information%2520files%2520will%2520be%2520freely%2520available%2520online,%2520and%2520any%2520third%2520party%2520is%2520permitted%2520to%2520access,%2520download,%2520copy,%2520distribute,%2520and%2520use%2520these%2520materials%2520in%2520any%2520way,%2520even%2520commercially,%2520with%2520proper%2520attribution.%2520For%2520these%2520reasons,%2520we%2520cannot%2520publish%2520previously%2520copyrighted%2520maps%2520or%2520satellite%2520images%2520created%2520using%2520proprietary%2520data,%2520such%2520as%2520Google%2520software%2520(Google%2520Maps,%2520Street%2520View,%2520and%2520Earth).%2520For%2520more%2520information,%2520see%2520our%2520copyright%2520guidelines:%2520http:%2Fjournals.plos.org%2Fplosone%2Fs%2Flicenses-and-copyright.%250b%250bWe%2520require%2520you%2520to%2520either%2520(1)%2520present%2520written%2520permission%2520from%2520the%2520copyright%2520holder%2520to%2520publish%2520these%2520figures%2520specifically%2520under%2520the%2520CC%2520BY%25204.0%2520license,%2520or%2520(2)%2520remove%2520the%2520figures%2520from%2520your%2520submission:%250b%250ba.%2520You%2520may%2520seek%2520permission%2520from%2520the%2520original%2520copyright%2520holder%2520of%2520Figure%25201%2520to%2520publish%2520the%2520content%2520specifically%2520under%2520the%2520CC%2520BY%25204.0%2520license.%2520%250b%250bWe%2520recommend%2520that%2520you%2520contact%2520the%2520original%2520copyright%2520holder%2520with%2520the%2520Content%2520Permission%2520Form%2520(http:%2Fjournals.plos.org%2Fplosone%2Fs%2Ffile%3Fid=7c09%2Fcontent-permission-form.pdf)%2520and%2520the%2520following%2520text:%250b%E2%80%9CI%2520request%2520permission%2520for%2520the%2520open-access%2520journal%2520PLOS%2520ONE%2520to%2520publish%2520XXX%2520under%2520the%2520Creative%2520Commons%2520Attribution%2520License%2520(CCAL)%2520CC%2520BY%25204.0%2520(http:%2F%2Fcreativecommons.org%2Flicenses%2Fby%2F4.0%2F).%2520Please%2520be%2520aware%2520that%2520this%2520license%2520allows%2520unrestricted%2520use%2520and%2520distribution,%2520even%2520commercially,%2520by%2520third%2520parties.%2520Please%2520reply%2520and%2520provide%2520explicit%2520written%2520permission%2520to%2520publish%2520XXX%2520under%2520a%2520CC%2520BY%2520license%2520and%2520complete%2520the%2520attached%2520form.%E2%80%9D%250b%250bPlease%2520upload%2520the%2520completed%2520Content%2520Permission%2520Form%2520or%2520other%2520proof%2520of%2520granted%2520permissions%2520as%2520an%2520%2522Other%2522%2520file%2520with%2520your%2520submission.%250b%250bIn%2520the%2520figure%2520caption%2520of%2520the%2520copyrighted%2520figure,%2520please%2520include%2520the%2520following%2520text:%2520%E2%80%9CReprinted%2520from%2520%255bref%255d%2520under%2520a%2520CC%2520BY%2520license,%2520with%2520permission%2520from%2520%255bname%2520of%2520publisher%255d,%2520original%2520copyright%2520%255boriginal%2520copyright%2520year%255d.%E2%80%9D%250b%250bb.%2520If%2520you%2520are%2520unable%2520to%2520obtain%2520permiss/1/010f0195d4f70bf9-6543d57e-4a22-449b-8de4-f487457c29e1-000000/KQjRjZVLS913SYbFlnS7sIRF7FE8EpSESWorl1I1cbY=204)
[Please upload the completed Content Permission Form or other proof of granted permissions as an "Other" file with your submission.](http://track.editorialmanager.com/CL0/We%2520note%2520that%2520Figure%25201%2520in%2520your%2520submission%2520contain%2520%255bmap%2Fsatellite%255d%2520images%2520which%2520may%2520be%2520copyrighted.%2520All%2520PLOS%2520content%2520is%2520published%2520under%2520the%2520Creative%2520Commons%2520Attribution%2520License%2520(CC%2520BY%25204.0),%2520which%2520means%2520that%2520the%2520manuscript,%2520images,%2520and%2520Supporting%2520Information%2520files%2520will%2520be%2520freely%2520available%2520online,%2520and%2520any%2520third%2520party%2520is%2520permitted%2520to%2520access,%2520download,%2520copy,%2520distribute,%2520and%2520use%2520these%2520materials%2520in%2520any%2520way,%2520even%2520commercially,%2520with%2520proper%2520attribution.%2520For%2520these%2520reasons,%2520we%2520cannot%2520publish%2520previously%2520copyrighted%2520maps%2520or%2520satellite%2520images%2520created%2520using%2520proprietary%2520data,%2520such%2520as%2520Google%2520software%2520(Google%2520Maps,%2520Street%2520View,%2520and%2520Earth).%2520For%2520more%2520information,%2520see%2520our%2520copyright%2520guidelines:%2520http:%2Fjournals.plos.org%2Fplosone%2Fs%2Flicenses-and-copyright.%250b%250bWe%2520require%2520you%2520to%2520either%2520(1)%2520present%2520written%2520permission%2520from%2520the%2520copyright%2520holder%2520to%2520publish%2520these%2520figures%2520specifically%2520under%2520the%2520CC%2520BY%25204.0%2520license,%2520or%2520(2)%2520remove%2520the%2520figures%2520from%2520your%2520submission:%250b%250ba.%2520You%2520may%2520seek%2520permission%2520from%2520the%2520original%2520copyright%2520holder%2520of%2520Figure%25201%2520to%2520publish%2520the%2520content%2520specifically%2520under%2520the%2520CC%2520BY%25204.0%2520license.%2520%250b%250bWe%2520recommend%2520that%2520you%2520contact%2520the%2520original%2520copyright%2520holder%2520with%2520the%2520Content%2520Permission%2520Form%2520(http:%2Fjournals.plos.org%2Fplosone%2Fs%2Ffile%3Fid=7c09%2Fcontent-permission-form.pdf)%2520and%2520the%2520following%2520text:%250b%E2%80%9CI%2520request%2520permission%2520for%2520the%2520open-access%2520journal%2520PLOS%2520ONE%2520to%2520publish%2520XXX%2520under%2520the%2520Creative%2520Commons%2520Attribution%2520License%2520(CCAL)%2520CC%2520BY%25204.0%2520(http:%2F%2Fcreativecommons.org%2Flicenses%2Fby%2F4.0%2F).%2520Please%2520be%2520aware%2520that%2520this%2520license%2520allows%2520unrestricted%2520use%2520and%2520distribution,%2520even%2520commercially,%2520by%2520third%2520parties.%2520Please%2520reply%2520and%2520provide%2520explicit%2520written%2520permission%2520to%2520publish%2520XXX%2520under%2520a%2520CC%2520BY%2520license%2520and%2520complete%2520the%2520attached%2520form.%E2%80%9D%250b%250bPlease%2520upload%2520the%2520completed%2520Content%2520Permission%2520Form%2520or%2520other%2520proof%2520of%2520granted%2520permissions%2520as%2520an%2520%2522Other%2522%2520file%2520with%2520your%2520submission.%250b%250bIn%2520the%2520figure%2520caption%2520of%2520the%2520copyrighted%2520figure,%2520please%2520include%2520the%2520following%2520text:%2520%E2%80%9CReprinted%2520from%2520%255bref%255d%2520under%2520a%2520CC%2520BY%2520license,%2520with%2520permission%2520from%2520%255bname%2520of%2520publisher%255d,%2520original%2520copyright%2520%255boriginal%2520copyright%2520year%255d.%E2%80%9D%250b%250bb.%2520If%2520you%2520are%2520unable%2520to%2520obtain%2520permiss/1/010f0195d4f70bf9-6543d57e-4a22-449b-8de4-f487457c29e1-000000/KQjRjZVLS913SYbFlnS7sIRF7FE8EpSESWorl1I1cbY=204)
[In the figure caption of the copyrighted figure, please include the following text: “Reprinted from [ref] under a CC BY license, with permission from [name of publisher], original copyright [original copyright year].”](http://track.editorialmanager.com/CL0/We%2520note%2520that%2520Figure%25201%2520in%2520your%2520submission%2520contain%2520%255bmap%2Fsatellite%255d%2520images%2520which%2520may%2520be%2520copyrighted.%2520All%2520PLOS%2520content%2520is%2520published%2520under%2520the%2520Creative%2520Commons%2520Attribution%2520License%2520(CC%2520BY%25204.0),%2520which%2520means%2520that%2520the%2520manuscript,%2520images,%2520and%2520Supporting%2520Information%2520files%2520will%2520be%2520freely%2520available%2520online,%2520and%2520any%2520third%2520party%2520is%2520permitted%2520to%2520access,%2520download,%2520copy,%2520distribute,%2520and%2520use%2520these%2520materials%2520in%2520any%2520way,%2520even%2520commercially,%2520with%2520proper%2520attribution.%2520For%2520these%2520reasons,%2520we%2520cannot%2520publish%2520previously%2520copyrighted%2520maps%2520or%2520satellite%2520images%2520created%2520using%2520proprietary%2520data,%2520such%2520as%2520Google%2520software%2520(Google%2520Maps,%2520Street%2520View,%2520and%2520Earth).%2520For%2520more%2520information,%2520see%2520our%2520copyright%2520guidelines:%2520http:%2Fjournals.plos.org%2Fplosone%2Fs%2Flicenses-and-copyright.%250b%250bWe%2520require%2520you%2520to%2520either%2520(1)%2520present%2520written%2520permission%2520from%2520the%2520copyright%2520holder%2520to%2520publish%2520these%2520figures%2520specifically%2520under%2520the%2520CC%2520BY%25204.0%2520license,%2520or%2520(2)%2520remove%2520the%2520figures%2520from%2520your%2520submission:%250b%250ba.%2520You%2520may%2520seek%2520permission%2520from%2520the%2520original%2520copyright%2520holder%2520of%2520Figure%25201%2520to%2520publish%2520the%2520content%2520specifically%2520under%2520the%2520CC%2520BY%25204.0%2520license.%2520%250b%250bWe%2520recommend%2520that%2520you%2520contact%2520the%2520original%2520copyright%2520holder%2520with%2520the%2520Content%2520Permission%2520Form%2520(http:%2Fjournals.plos.org%2Fplosone%2Fs%2Ffile%3Fid=7c09%2Fcontent-permission-form.pdf)%2520and%2520the%2520following%2520text:%250b%E2%80%9CI%2520request%2520permission%2520for%2520the%2520open-access%2520journal%2520PLOS%2520ONE%2520to%2520publish%2520XXX%2520under%2520the%2520Creative%2520Commons%2520Attribution%2520License%2520(CCAL)%2520CC%2520BY%25204.0%2520(http:%2F%2Fcreativecommons.org%2Flicenses%2Fby%2F4.0%2F).%2520Please%2520be%2520aware%2520that%2520this%2520license%2520allows%2520unrestricted%2520use%2520and%2520distribution,%2520even%2520commercially,%2520by%2520third%2520parties.%2520Please%2520reply%2520and%2520provide%2520explicit%2520written%2520permission%2520to%2520publish%2520XXX%2520under%2520a%2520CC%2520BY%2520license%2520and%2520complete%2520the%2520attached%2520form.%E2%80%9D%250b%250bPlease%2520upload%2520the%2520completed%2520Content%2520Permission%2520Form%2520or%2520other%2520proof%2520of%2520granted%2520permissions%2520as%2520an%2520%2522Other%2522%2520file%2520with%2520your%2520submission.%250b%250bIn%2520the%2520figure%2520caption%2520of%2520the%2520copyrighted%2520figure,%2520please%2520include%2520the%2520following%2520text:%2520%E2%80%9CReprinted%2520from%2520%255bref%255d%2520under%2520a%2520CC%2520BY%2520license,%2520with%2520permission%2520from%2520%255bname%2520of%2520publisher%255d,%2520original%2520copyright%2520%255boriginal%2520copyright%2520year%255d.%E2%80%9D%250b%250bb.%2520If%2520you%2520are%2520unable%2520to%2520obtain%2520permiss/1/010f0195d4f70bf9-6543d57e-4a22-449b-8de4-f487457c29e1-000000/KQjRjZVLS913SYbFlnS7sIRF7FE8EpSESWorl1I1cbY=204)

[b. If you are unable to obtain permission from the original copyright holder to publish these figures under the CC BY 4.0 license or if the copyright holder’s requirements are incompatible with the CC BY 4.0 license, please either i) remove the figure or ii) supply a replacement figure that complies with the CC BY 4.0 license. Please check copyright information on all replacement figures and update the figure caption with source information. If applicable, please specify in the figure caption text when a figure is similar but not identical to the original image and is therefore for illustrative purposes only.](http://track.editorialmanager.com/CL0/We%2520note%2520that%2520Figure%25201%2520in%2520your%2520submission%2520contain%2520%255bmap%2Fsatellite%255d%2520images%2520which%2520may%2520be%2520copyrighted.%2520All%2520PLOS%2520content%2520is%2520published%2520under%2520the%2520Creative%2520Commons%2520Attribution%2520License%2520(CC%2520BY%25204.0),%2520which%2520means%2520that%2520the%2520manuscript,%2520images,%2520and%2520Supporting%2520Information%2520files%2520will%2520be%2520freely%2520available%2520online,%2520and%2520any%2520third%2520party%2520is%2520permitted%2520to%2520access,%2520download,%2520copy,%2520distribute,%2520and%2520use%2520these%2520materials%2520in%2520any%2520way,%2520even%2520commercially,%2520with%2520proper%2520attribution.%2520For%2520these%2520reasons,%2520we%2520cannot%2520publish%2520previously%2520copyrighted%2520maps%2520or%2520satellite%2520images%2520created%2520using%2520proprietary%2520data,%2520such%2520as%2520Google%2520software%2520(Google%2520Maps,%2520Street%2520View,%2520and%2520Earth).%2520For%2520more%2520information,%2520see%2520our%2520copyright%2520guidelines:%2520http:%2Fjournals.plos.org%2Fplosone%2Fs%2Flicenses-and-copyright.%250b%250bWe%2520require%2520you%2520to%2520either%2520(1)%2520present%2520written%2520permission%2520from%2520the%2520copyright%2520holder%2520to%2520publish%2520these%2520figures%2520specifically%2520under%2520the%2520CC%2520BY%25204.0%2520license,%2520or%2520(2)%2520remove%2520the%2520figures%2520from%2520your%2520submission:%250b%250ba.%2520You%2520may%2520seek%2520permission%2520from%2520the%2520original%2520copyright%2520holder%2520of%2520Figure%25201%2520to%2520publish%2520the%2520content%2520specifically%2520under%2520the%2520CC%2520BY%25204.0%2520license.%2520%250b%250bWe%2520recommend%2520that%2520you%2520contact%2520the%2520original%2520copyright%2520holder%2520with%2520the%2520Content%2520Permission%2520Form%2520(http:%2Fjournals.plos.org%2Fplosone%2Fs%2Ffile%3Fid=7c09%2Fcontent-permission-form.pdf)%2520and%2520the%2520following%2520text:%250b%E2%80%9CI%2520request%2520permission%2520for%2520the%2520open-access%2520journal%2520PLOS%2520ONE%2520to%2520publish%2520XXX%2520under%2520the%2520Creative%2520Commons%2520Attribution%2520License%2520(CCAL)%2520CC%2520BY%25204.0%2520(http:%2F%2Fcreativecommons.org%2Flicenses%2Fby%2F4.0%2F).%2520Please%2520be%2520aware%2520that%2520this%2520license%2520allows%2520unrestricted%2520use%2520and%2520distribution,%2520even%2520commercially,%2520by%2520third%2520parties.%2520Please%2520reply%2520and%2520provide%2520explicit%2520written%2520permission%2520to%2520publish%2520XXX%2520under%2520a%2520CC%2520BY%2520license%2520and%2520complete%2520the%2520attached%2520form.%E2%80%9D%250b%250bPlease%2520upload%2520the%2520completed%2520Content%2520Permission%2520Form%2520or%2520other%2520proof%2520of%2520granted%2520permissions%2520as%2520an%2520%2522Other%2522%2520file%2520with%2520your%2520submission.%250b%250bIn%2520the%2520figure%2520caption%2520of%2520the%2520copyrighted%2520figure,%2520please%2520include%2520the%2520following%2520text:%2520%E2%80%9CReprinted%2520from%2520%255bref%255d%2520under%2520a%2520CC%2520BY%2520license,%2520with%2520permission%2520from%2520%255bname%2520of%2520publisher%255d,%2520original%2520copyright%2520%255boriginal%2520copyright%2520year%255d.%E2%80%9D%250b%250bb.%2520If%2520you%2520are%2520unable%2520to%2520obtain%2520permiss/1/010f0195d4f70bf9-6543d57e-4a22-449b-8de4-f487457c29e1-000000/KQjRjZVLS913SYbFlnS7sIRF7FE8EpSESWorl1I1cbY=204)
[The following resources for replacing copyrighted map figures may be helpful:](http://track.editorialmanager.com/CL0/We%2520note%2520that%2520Figure%25201%2520in%2520your%2520submission%2520contain%2520%255bmap%2Fsatellite%255d%2520images%2520which%2520may%2520be%2520copyrighted.%2520All%2520PLOS%2520content%2520is%2520published%2520under%2520the%2520Creative%2520Commons%2520Attribution%2520License%2520(CC%2520BY%25204.0),%2520which%2520means%2520that%2520the%2520manuscript,%2520images,%2520and%2520Supporting%2520Information%2520files%2520will%2520be%2520freely%2520available%2520online,%2520and%2520any%2520third%2520party%2520is%2520permitted%2520to%2520access,%2520download,%2520copy,%2520distribute,%2520and%2520use%2520these%2520materials%2520in%2520any%2520way,%2520even%2520commercially,%2520with%2520proper%2520attribution.%2520For%2520these%2520reasons,%2520we%2520cannot%2520publish%2520previously%2520copyrighted%2520maps%2520or%2520satellite%2520images%2520created%2520using%2520proprietary%2520data,%2520such%2520as%2520Google%2520software%2520(Google%2520Maps,%2520Street%2520View,%2520and%2520Earth).%2520For%2520more%2520information,%2520see%2520our%2520copyright%2520guidelines:%2520http:%2Fjournals.plos.org%2Fplosone%2Fs%2Flicenses-and-copyright.%250b%250bWe%2520require%2520you%2520to%2520either%2520(1)%2520present%2520written%2520permission%2520from%2520the%2520copyright%2520holder%2520to%2520publish%2520these%2520figures%2520specifically%2520under%2520the%2520CC%2520BY%25204.0%2520license,%2520or%2520(2)%2520remove%2520the%2520figures%2520from%2520your%2520submission:%250b%250ba.%2520You%2520may%2520seek%2520permission%2520from%2520the%2520original%2520copyright%2520holder%2520of%2520Figure%25201%2520to%2520publish%2520the%2520content%2520specifically%2520under%2520the%2520CC%2520BY%25204.0%2520license.%2520%250b%250bWe%2520recommend%2520that%2520you%2520contact%2520the%2520original%2520copyright%2520holder%2520with%2520the%2520Content%2520Permission%2520Form%2520(http:%2Fjournals.plos.org%2Fplosone%2Fs%2Ffile%3Fid=7c09%2Fcontent-permission-form.pdf)%2520and%2520the%2520following%2520text:%250b%E2%80%9CI%2520request%2520permission%2520for%2520the%2520open-access%2520journal%2520PLOS%2520ONE%2520to%2520publish%2520XXX%2520under%2520the%2520Creative%2520Commons%2520Attribution%2520License%2520(CCAL)%2520CC%2520BY%25204.0%2520(http:%2F%2Fcreativecommons.org%2Flicenses%2Fby%2F4.0%2F).%2520Please%2520be%2520aware%2520that%2520this%2520license%2520allows%2520unrestricted%2520use%2520and%2520distribution,%2520even%2520commercially,%2520by%2520third%2520parties.%2520Please%2520reply%2520and%2520provide%2520explicit%2520written%2520permission%2520to%2520publish%2520XXX%2520under%2520a%2520CC%2520BY%2520license%2520and%2520complete%2520the%2520attached%2520form.%E2%80%9D%250b%250bPlease%2520upload%2520the%2520completed%2520Content%2520Permission%2520Form%2520or%2520other%2520proof%2520of%2520granted%2520permissions%2520as%2520an%2520%2522Other%2522%2520file%2520with%2520your%2520submission.%250b%250bIn%2520the%2520figure%2520caption%2520of%2520the%2520copyrighted%2520figure,%2520please%2520include%2520the%2520following%2520text:%2520%E2%80%9CReprinted%2520from%2520%255bref%255d%2520under%2520a%2520CC%2520BY%2520license,%2520with%2520permission%2520from%2520%255bname%2520of%2520publisher%255d,%2520original%2520copyright%2520%255boriginal%2520copyright%2520year%255d.%E2%80%9D%250b%250bb.%2520If%2520you%2520are%2520unable%2520to%2520obtain%2520permiss/1/010f0195d4f70bf9-6543d57e-4a22-449b-8de4-f487457c29e1-000000/KQjRjZVLS913SYbFlnS7sIRF7FE8EpSESWorl1I1cbY=204)

[USGS National Map Viewer (public domain): http://viewer.nationalmap.gov/viewer/](http://track.editorialmanager.com/CL0/We%2520note%2520that%2520Figure%25201%2520in%2520your%2520submission%2520contain%2520%255bmap%2Fsatellite%255d%2520images%2520which%2520may%2520be%2520copyrighted.%2520All%2520PLOS%2520content%2520is%2520published%2520under%2520the%2520Creative%2520Commons%2520Attribution%2520License%2520(CC%2520BY%25204.0),%2520which%2520means%2520that%2520the%2520manuscript,%2520images,%2520and%2520Supporting%2520Information%2520files%2520will%2520be%2520freely%2520available%2520online,%2520and%2520any%2520third%2520party%2520is%2520permitted%2520to%2520access,%2520download,%2520copy,%2520distribute,%2520and%2520use%2520these%2520materials%2520in%2520any%2520way,%2520even%2520commercially,%2520with%2520proper%2520attribution.%2520For%2520these%2520reasons,%2520we%2520cannot%2520publish%2520previously%2520copyrighted%2520maps%2520or%2520satellite%2520images%2520created%2520using%2520proprietary%2520data,%2520such%2520as%2520Google%2520software%2520(Google%2520Maps,%2520Street%2520View,%2520and%2520Earth).%2520For%2520more%2520information,%2520see%2520our%2520copyright%2520guidelines:%2520http:%2Fjournals.plos.org%2Fplosone%2Fs%2Flicenses-and-copyright.%250b%250bWe%2520require%2520you%2520to%2520either%2520(1)%2520present%2520written%2520permission%2520from%2520the%2520copyright%2520holder%2520to%2520publish%2520these%2520figures%2520specifically%2520under%2520the%2520CC%2520BY%25204.0%2520license,%2520or%2520(2)%2520remove%2520the%2520figures%2520from%2520your%2520submission:%250b%250ba.%2520You%2520may%2520seek%2520permission%2520from%2520the%2520original%2520copyright%2520holder%2520of%2520Figure%25201%2520to%2520publish%2520the%2520content%2520specifically%2520under%2520the%2520CC%2520BY%25204.0%2520license.%2520%250b%250bWe%2520recommend%2520that%2520you%2520contact%2520the%2520original%2520copyright%2520holder%2520with%2520the%2520Content%2520Permission%2520Form%2520(http:%2Fjournals.plos.org%2Fplosone%2Fs%2Ffile%3Fid=7c09%2Fcontent-permission-form.pdf)%2520and%2520the%2520following%2520text:%250b%E2%80%9CI%2520request%2520permission%2520for%2520the%2520open-access%2520journal%2520PLOS%2520ONE%2520to%2520publish%2520XXX%2520under%2520the%2520Creative%2520Commons%2520Attribution%2520License%2520(CCAL)%2520CC%2520BY%25204.0%2520(http:%2F%2Fcreativecommons.org%2Flicenses%2Fby%2F4.0%2F).%2520Please%2520be%2520aware%2520that%2520this%2520license%2520allows%2520unrestricted%2520use%2520and%2520distribution,%2520even%2520commercially,%2520by%2520third%2520parties.%2520Please%2520reply%2520and%2520provide%2520explicit%2520written%2520permission%2520to%2520publish%2520XXX%2520under%2520a%2520CC%2520BY%2520license%2520and%2520complete%2520the%2520attached%2520form.%E2%80%9D%250b%250bPlease%2520upload%2520the%2520completed%2520Content%2520Permission%2520Form%2520or%2520other%2520proof%2520of%2520granted%2520permissions%2520as%2520an%2520%2522Other%2522%2520file%2520with%2520your%2520submission.%250b%250bIn%2520the%2520figure%2520caption%2520of%2520the%2520copyrighted%2520figure,%2520please%2520include%2520the%2520following%2520text:%2520%E2%80%9CReprinted%2520from%2520%255bref%255d%2520under%2520a%2520CC%2520BY%2520license,%2520with%2520permission%2520from%2520%255bname%2520of%2520publisher%255d,%2520original%2520copyright%2520%255boriginal%2520copyright%2520year%255d.%E2%80%9D%250b%250bb.%2520If%2520you%2520are%2520unable%2520to%2520obtain%2520permiss/1/010f0195d4f70bf9-6543d57e-4a22-449b-8de4-f487457c29e1-000000/KQjRjZVLS913SYbFlnS7sIRF7FE8EpSESWorl1I1cbY=204)
[The Gateway to Astronaut Photography of Earth (public domain): http://eol.jsc.nasa.gov/sseop/clickmap/](http://track.editorialmanager.com/CL0/We%2520note%2520that%2520Figure%25201%2520in%2520your%2520submission%2520contain%2520%255bmap%2Fsatellite%255d%2520images%2520which%2520may%2520be%2520copyrighted.%2520All%2520PLOS%2520content%2520is%2520published%2520under%2520the%2520Creative%2520Commons%2520Attribution%2520License%2520(CC%2520BY%25204.0),%2520which%2520means%2520that%2520the%2520manuscript,%2520images,%2520and%2520Supporting%2520Information%2520files%2520will%2520be%2520freely%2520available%2520online,%2520and%2520any%2520third%2520party%2520is%2520permitted%2520to%2520access,%2520download,%2520copy,%2520distribute,%2520and%2520use%2520these%2520materials%2520in%2520any%2520way,%2520even%2520commercially,%2520with%2520proper%2520attribution.%2520For%2520these%2520reasons,%2520we%2520cannot%2520publish%2520previously%2520copyrighted%2520maps%2520or%2520satellite%2520images%2520created%2520using%2520proprietary%2520data,%2520such%2520as%2520Google%2520software%2520(Google%2520Maps,%2520Street%2520View,%2520and%2520Earth).%2520For%2520more%2520information,%2520see%2520our%2520copyright%2520guidelines:%2520http:%2Fjournals.plos.org%2Fplosone%2Fs%2Flicenses-and-copyright.%250b%250bWe%2520require%2520you%2520to%2520either%2520(1)%2520present%2520written%2520permission%2520from%2520the%2520copyright%2520holder%2520to%2520publish%2520these%2520figures%2520specifically%2520under%2520the%2520CC%2520BY%25204.0%2520license,%2520or%2520(2)%2520remove%2520the%2520figures%2520from%2520your%2520submission:%250b%250ba.%2520You%2520may%2520seek%2520permission%2520from%2520the%2520original%2520copyright%2520holder%2520of%2520Figure%25201%2520to%2520publish%2520the%2520content%2520specifically%2520under%2520the%2520CC%2520BY%25204.0%2520license.%2520%250b%250bWe%2520recommend%2520that%2520you%2520contact%2520the%2520original%2520copyright%2520holder%2520with%2520the%2520Content%2520Permission%2520Form%2520(http:%2Fjournals.plos.org%2Fplosone%2Fs%2Ffile%3Fid=7c09%2Fcontent-permission-form.pdf)%2520and%2520the%2520following%2520text:%250b%E2%80%9CI%2520request%2520permission%2520for%2520the%2520open-access%2520journal%2520PLOS%2520ONE%2520to%2520publish%2520XXX%2520under%2520the%2520Creative%2520Commons%2520Attribution%2520License%2520(CCAL)%2520CC%2520BY%25204.0%2520(http:%2F%2Fcreativecommons.org%2Flicenses%2Fby%2F4.0%2F).%2520Please%2520be%2520aware%2520that%2520this%2520license%2520allows%2520unrestricted%2520use%2520and%2520distribution,%2520even%2520commercially,%2520by%2520third%2520parties.%2520Please%2520reply%2520and%2520provide%2520explicit%2520written%2520permission%2520to%2520publish%2520XXX%2520under%2520a%2520CC%2520BY%2520license%2520and%2520complete%2520the%2520attached%2520form.%E2%80%9D%250b%250bPlease%2520upload%2520the%2520completed%2520Content%2520Permission%2520Form%2520or%2520other%2520proof%2520of%2520granted%2520permissions%2520as%2520an%2520%2522Other%2522%2520file%2520with%2520your%2520submission.%250b%250bIn%2520the%2520figure%2520caption%2520of%2520the%2520copyrighted%2520figure,%2520please%2520include%2520the%2520following%2520text:%2520%E2%80%9CReprinted%2520from%2520%255bref%255d%2520under%2520a%2520CC%2520BY%2520license,%2520with%2520permission%2520from%2520%255bname%2520of%2520publisher%255d,%2520original%2520copyright%2520%255boriginal%2520copyright%2520year%255d.%E2%80%9D%250b%250bb.%2520If%2520you%2520are%2520unable%2520to%2520obtain%2520permiss/1/010f0195d4f70bf9-6543d57e-4a22-449b-8de4-f487457c29e1-000000/KQjRjZVLS913SYbFlnS7sIRF7FE8EpSESWorl1I1cbY=204)
[Maps at the CIA (public domain): https://www.cia.gov/library/publications/the-world-factbook/index.html and https://www.cia.gov/library/publications/cia-maps-publications/index.html](http://track.editorialmanager.com/CL0/We%2520note%2520that%2520Figure%25201%2520in%2520your%2520submission%2520contain%2520%255bmap%2Fsatellite%255d%2520images%2520which%2520may%2520be%2520copyrighted.%2520All%2520PLOS%2520content%2520is%2520published%2520under%2520the%2520Creative%2520Commons%2520Attribution%2520License%2520(CC%2520BY%25204.0),%2520which%2520means%2520that%2520the%2520manuscript,%2520images,%2520and%2520Supporting%2520Information%2520files%2520will%2520be%2520freely%2520available%2520online,%2520and%2520any%2520third%2520party%2520is%2520permitted%2520to%2520access,%2520download,%2520copy,%2520distribute,%2520and%2520use%2520these%2520materials%2520in%2520any%2520way,%2520even%2520commercially,%2520with%2520proper%2520attribution.%2520For%2520these%2520reasons,%2520we%2520cannot%2520publish%2520previously%2520copyrighted%2520maps%2520or%2520satellite%2520images%2520created%2520using%2520proprietary%2520data,%2520such%2520as%2520Google%2520software%2520(Google%2520Maps,%2520Street%2520View,%2520and%2520Earth).%2520For%2520more%2520information,%2520see%2520our%2520copyright%2520guidelines:%2520http:%2Fjournals.plos.org%2Fplosone%2Fs%2Flicenses-and-copyright.%250b%250bWe%2520require%2520you%2520to%2520either%2520(1)%2520present%2520written%2520permission%2520from%2520the%2520copyright%2520holder%2520to%2520publish%2520these%2520figures%2520specifically%2520under%2520the%2520CC%2520BY%25204.0%2520license,%2520or%2520(2)%2520remove%2520the%2520figures%2520from%2520your%2520submission:%250b%250ba.%2520You%2520may%2520seek%2520permission%2520from%2520the%2520original%2520copyright%2520holder%2520of%2520Figure%25201%2520to%2520publish%2520the%2520content%2520specifically%2520under%2520the%2520CC%2520BY%25204.0%2520license.%2520%250b%250bWe%2520recommend%2520that%2520you%2520contact%2520the%2520original%2520copyright%2520holder%2520with%2520the%2520Content%2520Permission%2520Form%2520(http:%2Fjournals.plos.org%2Fplosone%2Fs%2Ffile%3Fid=7c09%2Fcontent-permission-form.pdf)%2520and%2520the%2520following%2520text:%250b%E2%80%9CI%2520request%2520permission%2520for%2520the%2520open-access%2520journal%2520PLOS%2520ONE%2520to%2520publish%2520XXX%2520under%2520the%2520Creative%2520Commons%2520Attribution%2520License%2520(CCAL)%2520CC%2520BY%25204.0%2520(http:%2F%2Fcreativecommons.org%2Flicenses%2Fby%2F4.0%2F).%2520Please%2520be%2520aware%2520that%2520this%2520license%2520allows%2520unrestricted%2520use%2520and%2520distribution,%2520even%2520commercially,%2520by%2520third%2520parties.%2520Please%2520reply%2520and%2520provide%2520explicit%2520written%2520permission%2520to%2520publish%2520XXX%2520under%2520a%2520CC%2520BY%2520license%2520and%2520complete%2520the%2520attached%2520form.%E2%80%9D%250b%250bPlease%2520upload%2520the%2520completed%2520Content%2520Permission%2520Form%2520or%2520other%2520proof%2520of%2520granted%2520permissions%2520as%2520an%2520%2522Other%2522%2520file%2520with%2520your%2520submission.%250b%250bIn%2520the%2520figure%2520caption%2520of%2520the%2520copyrighted%2520figure,%2520please%2520include%2520the%2520following%2520text:%2520%E2%80%9CReprinted%2520from%2520%255bref%255d%2520under%2520a%2520CC%2520BY%2520license,%2520with%2520permission%2520from%2520%255bname%2520of%2520publisher%255d,%2520original%2520copyright%2520%255boriginal%2520copyright%2520year%255d.%E2%80%9D%250b%250bb.%2520If%2520you%2520are%2520unable%2520to%2520obtain%2520permiss/1/010f0195d4f70bf9-6543d57e-4a22-449b-8de4-f487457c29e1-000000/KQjRjZVLS913SYbFlnS7sIRF7FE8EpSESWorl1I1cbY=204)
[NASA Earth Observatory (public domain): http://earthobservatory.nasa.gov/](http://track.editorialmanager.com/CL0/We%2520note%2520that%2520Figure%25201%2520in%2520your%2520submission%2520contain%2520%255bmap%2Fsatellite%255d%2520images%2520which%2520may%2520be%2520copyrighted.%2520All%2520PLOS%2520content%2520is%2520published%2520under%2520the%2520Creative%2520Commons%2520Attribution%2520License%2520(CC%2520BY%25204.0),%2520which%2520means%2520that%2520the%2520manuscript,%2520images,%2520and%2520Supporting%2520Information%2520files%2520will%2520be%2520freely%2520available%2520online,%2520and%2520any%2520third%2520party%2520is%2520permitted%2520to%2520access,%2520download,%2520copy,%2520distribute,%2520and%2520use%2520these%2520materials%2520in%2520any%2520way,%2520even%2520commercially,%2520with%2520proper%2520attribution.%2520For%2520these%2520reasons,%2520we%2520cannot%2520publish%2520previously%2520copyrighted%2520maps%2520or%2520satellite%2520images%2520created%2520using%2520proprietary%2520data,%2520such%2520as%2520Google%2520software%2520(Google%2520Maps,%2520Street%2520View,%2520and%2520Earth).%2520For%2520more%2520information,%2520see%2520our%2520copyright%2520guidelines:%2520http:%2Fjournals.plos.org%2Fplosone%2Fs%2Flicenses-and-copyright.%250b%250bWe%2520require%2520you%2520to%2520either%2520(1)%2520present%2520written%2520permission%2520from%2520the%2520copyright%2520holder%2520to%2520publish%2520these%2520figures%2520specifically%2520under%2520the%2520CC%2520BY%25204.0%2520license,%2520or%2520(2)%2520remove%2520the%2520figures%2520from%2520your%2520submission:%250b%250ba.%2520You%2520may%2520seek%2520permission%2520from%2520the%2520original%2520copyright%2520holder%2520of%2520Figure%25201%2520to%2520publish%2520the%2520content%2520specifically%2520under%2520the%2520CC%2520BY%25204.0%2520license.%2520%250b%250bWe%2520recommend%2520that%2520you%2520contact%2520the%2520original%2520copyright%2520holder%2520with%2520the%2520Content%2520Permission%2520Form%2520(http:%2Fjournals.plos.org%2Fplosone%2Fs%2Ffile%3Fid=7c09%2Fcontent-permission-form.pdf)%2520and%2520the%2520following%2520text:%250b%E2%80%9CI%2520request%2520permission%2520for%2520the%2520open-access%2520journal%2520PLOS%2520ONE%2520to%2520publish%2520XXX%2520under%2520the%2520Creative%2520Commons%2520Attribution%2520License%2520(CCAL)%2520CC%2520BY%25204.0%2520(http:%2F%2Fcreativecommons.org%2Flicenses%2Fby%2F4.0%2F).%2520Please%2520be%2520aware%2520that%2520this%2520license%2520allows%2520unrestricted%2520use%2520and%2520distribution,%2520even%2520commercially,%2520by%2520third%2520parties.%2520Please%2520reply%2520and%2520provide%2520explicit%2520written%2520permission%2520to%2520publish%2520XXX%2520under%2520a%2520CC%2520BY%2520license%2520and%2520complete%2520the%2520attached%2520form.%E2%80%9D%250b%250bPlease%2520upload%2520the%2520completed%2520Content%2520Permission%2520Form%2520or%2520other%2520proof%2520of%2520granted%2520permissions%2520as%2520an%2520%2522Other%2522%2520file%2520with%2520your%2520submission.%250b%250bIn%2520the%2520figure%2520caption%2520of%2520the%2520copyrighted%2520figure,%2520please%2520include%2520the%2520following%2520text:%2520%E2%80%9CReprinted%2520from%2520%255bref%255d%2520under%2520a%2520CC%2520BY%2520license,%2520with%2520permission%2520from%2520%255bname%2520of%2520publisher%255d,%2520original%2520copyright%2520%255boriginal%2520copyright%2520year%255d.%E2%80%9D%250b%250bb.%2520If%2520you%2520are%2520unable%2520to%2520obtain%2520permiss/1/010f0195d4f70bf9-6543d57e-4a22-449b-8de4-f487457c29e1-000000/KQjRjZVLS913SYbFlnS7sIRF7FE8EpSESWorl1I1cbY=204)
[Landsat: http://landsat.visibleearth.nasa.gov/](http://track.editorialmanager.com/CL0/We%2520note%2520that%2520Figure%25201%2520in%2520your%2520submission%2520contain%2520%255bmap%2Fsatellite%255d%2520images%2520which%2520may%2520be%2520copyrighted.%2520All%2520PLOS%2520content%2520is%2520published%2520under%2520the%2520Creative%2520Commons%2520Attribution%2520License%2520(CC%2520BY%25204.0),%2520which%2520means%2520that%2520the%2520manuscript,%2520images,%2520and%2520Supporting%2520Information%2520files%2520will%2520be%2520freely%2520available%2520online,%2520and%2520any%2520third%2520party%2520is%2520permitted%2520to%2520access,%2520download,%2520copy,%2520distribute,%2520and%2520use%2520these%2520materials%2520in%2520any%2520way,%2520even%2520commercially,%2520with%2520proper%2520attribution.%2520For%2520these%2520reasons,%2520we%2520cannot%2520publish%2520previously%2520copyrighted%2520maps%2520or%2520satellite%2520images%2520created%2520using%2520proprietary%2520data,%2520such%2520as%2520Google%2520software%2520(Google%2520Maps,%2520Street%2520View,%2520and%2520Earth).%2520For%2520more%2520information,%2520see%2520our%2520copyright%2520guidelines:%2520http:%2Fjournals.plos.org%2Fplosone%2Fs%2Flicenses-and-copyright.%250b%250bWe%2520require%2520you%2520to%2520either%2520(1)%2520present%2520written%2520permission%2520from%2520the%2520copyright%2520holder%2520to%2520publish%2520these%2520figures%2520specifically%2520under%2520the%2520CC%2520BY%25204.0%2520license,%2520or%2520(2)%2520remove%2520the%2520figures%2520from%2520your%2520submission:%250b%250ba.%2520You%2520may%2520seek%2520permission%2520from%2520the%2520original%2520copyright%2520holder%2520of%2520Figure%25201%2520to%2520publish%2520the%2520content%2520specifically%2520under%2520the%2520CC%2520BY%25204.0%2520license.%2520%250b%250bWe%2520recommend%2520that%2520you%2520contact%2520the%2520original%2520copyright%2520holder%2520with%2520the%2520Content%2520Permission%2520Form%2520(http:%2Fjournals.plos.org%2Fplosone%2Fs%2Ffile%3Fid=7c09%2Fcontent-permission-form.pdf)%2520and%2520the%2520following%2520text:%250b%E2%80%9CI%2520request%2520permission%2520for%2520the%2520open-access%2520journal%2520PLOS%2520ONE%2520to%2520publish%2520XXX%2520under%2520the%2520Creative%2520Commons%2520Attribution%2520License%2520(CCAL)%2520CC%2520BY%25204.0%2520(http:%2F%2Fcreativecommons.org%2Flicenses%2Fby%2F4.0%2F).%2520Please%2520be%2520aware%2520that%2520this%2520license%2520allows%2520unrestricted%2520use%2520and%2520distribution,%2520even%2520commercially,%2520by%2520third%2520parties.%2520Please%2520reply%2520and%2520provide%2520explicit%2520written%2520permission%2520to%2520publish%2520XXX%2520under%2520a%2520CC%2520BY%2520license%2520and%2520complete%2520the%2520attached%2520form.%E2%80%9D%250b%250bPlease%2520upload%2520the%2520completed%2520Content%2520Permission%2520Form%2520or%2520other%2520proof%2520of%2520granted%2520permissions%2520as%2520an%2520%2522Other%2522%2520file%2520with%2520your%2520submission.%250b%250bIn%2520the%2520figure%2520caption%2520of%2520the%2520copyrighted%2520figure,%2520please%2520include%2520the%2520following%2520text:%2520%E2%80%9CReprinted%2520from%2520%255bref%255d%2520under%2520a%2520CC%2520BY%2520license,%2520with%2520permission%2520from%2520%255bname%2520of%2520publisher%255d,%2520original%2520copyright%2520%255boriginal%2520copyright%2520year%255d.%E2%80%9D%250b%250bb.%2520If%2520you%2520are%2520unable%2520to%2520obtain%2520permiss/1/010f0195d4f70bf9-6543d57e-4a22-449b-8de4-f487457c29e1-000000/KQjRjZVLS913SYbFlnS7sIRF7FE8EpSESWorl1I1cbY=204)
[USGS EROS (Earth Resources Observatory and Science (EROS) Center) (public domain): http://eros.usgs.gov/#](http://track.editorialmanager.com/CL0/We%2520note%2520that%2520Figure%25201%2520in%2520your%2520submission%2520contain%2520%255bmap%2Fsatellite%255d%2520images%2520which%2520may%2520be%2520copyrighted.%2520All%2520PLOS%2520content%2520is%2520published%2520under%2520the%2520Creative%2520Commons%2520Attribution%2520License%2520(CC%2520BY%25204.0),%2520which%2520means%2520that%2520the%2520manuscript,%2520images,%2520and%2520Supporting%2520Information%2520files%2520will%2520be%2520freely%2520available%2520online,%2520and%2520any%2520third%2520party%2520is%2520permitted%2520to%2520access,%2520download,%2520copy,%2520distribute,%2520and%2520use%2520these%2520materials%2520in%2520any%2520way,%2520even%2520commercially,%2520with%2520proper%2520attribution.%2520For%2520these%2520reasons,%2520we%2520cannot%2520publish%2520previously%2520copyrighted%2520maps%2520or%2520satellite%2520images%2520created%2520using%2520proprietary%2520data,%2520such%2520as%2520Google%2520software%2520(Google%2520Maps,%2520Street%2520View,%2520and%2520Earth).%2520For%2520more%2520information,%2520see%2520our%2520copyright%2520guidelines:%2520http:%2Fjournals.plos.org%2Fplosone%2Fs%2Flicenses-and-copyright.%250b%250bWe%2520require%2520you%2520to%2520either%2520(1)%2520present%2520written%2520permission%2520from%2520the%2520copyright%2520holder%2520to%2520publish%2520these%2520figures%2520specifically%2520under%2520the%2520CC%2520BY%25204.0%2520license,%2520or%2520(2)%2520remove%2520the%2520figures%2520from%2520your%2520submission:%250b%250ba.%2520You%2520may%2520seek%2520permission%2520from%2520the%2520original%2520copyright%2520holder%2520of%2520Figure%25201%2520to%2520publish%2520the%2520content%2520specifically%2520under%2520the%2520CC%2520BY%25204.0%2520license.%2520%250b%250bWe%2520recommend%2520that%2520you%2520contact%2520the%2520original%2520copyright%2520holder%2520with%2520the%2520Content%2520Permission%2520Form%2520(http:%2Fjournals.plos.org%2Fplosone%2Fs%2Ffile%3Fid=7c09%2Fcontent-permission-form.pdf)%2520and%2520the%2520following%2520text:%250b%E2%80%9CI%2520request%2520permission%2520for%2520the%2520open-access%2520journal%2520PLOS%2520ONE%2520to%2520publish%2520XXX%2520under%2520the%2520Creative%2520Commons%2520Attribution%2520License%2520(CCAL)%2520CC%2520BY%25204.0%2520(http:%2F%2Fcreativecommons.org%2Flicenses%2Fby%2F4.0%2F).%2520Please%2520be%2520aware%2520that%2520this%2520license%2520allows%2520unrestricted%2520use%2520and%2520distribution,%2520even%2520commercially,%2520by%2520third%2520parties.%2520Please%2520reply%2520and%2520provide%2520explicit%2520written%2520permission%2520to%2520publish%2520XXX%2520under%2520a%2520CC%2520BY%2520license%2520and%2520complete%2520the%2520attached%2520form.%E2%80%9D%250b%250bPlease%2520upload%2520the%2520completed%2520Content%2520Permission%2520Form%2520or%2520other%2520proof%2520of%2520granted%2520permissions%2520as%2520an%2520%2522Other%2522%2520file%2520with%2520your%2520submission.%250b%250bIn%2520the%2520figure%2520caption%2520of%2520the%2520copyrighted%2520figure,%2520please%2520include%2520the%2520following%2520text:%2520%E2%80%9CReprinted%2520from%2520%255bref%255d%2520under%2520a%2520CC%2520BY%2520license,%2520with%2520permission%2520from%2520%255bname%2520of%2520publisher%255d,%2520original%2520copyright%2520%255boriginal%2520copyright%2520year%255d.%E2%80%9D%250b%250bb.%2520If%2520you%2520are%2520unable%2520to%2520obtain%2520permiss/1/010f0195d4f70bf9-6543d57e-4a22-449b-8de4-f487457c29e1-000000/KQjRjZVLS913SYbFlnS7sIRF7FE8EpSESWorl1I1cbY=204)
[Natural Earth (public domain): http://www.naturalearthdata.com/](http://track.editorialmanager.com/CL0/We%2520note%2520that%2520Figure%25201%2520in%2520your%2520submission%2520contain%2520%255bmap%2Fsatellite%255d%2520images%2520which%2520may%2520be%2520copyrighted.%2520All%2520PLOS%2520content%2520is%2520published%2520under%2520the%2520Creative%2520Commons%2520Attribution%2520License%2520(CC%2520BY%25204.0),%2520which%2520means%2520that%2520the%2520manuscript,%2520images,%2520and%2520Supporting%2520Information%2520files%2520will%2520be%2520freely%2520available%2520online,%2520and%2520any%2520third%2520party%2520is%2520permitted%2520to%2520access,%2520download,%2520copy,%2520distribute,%2520and%2520use%2520these%2520materials%2520in%2520any%2520way,%2520even%2520commercially,%2520with%2520proper%2520attribution.%2520For%2520these%2520reasons,%2520we%2520cannot%2520publish%2520previously%2520copyrighted%2520maps%2520or%2520satellite%2520images%2520created%2520using%2520proprietary%2520data,%2520such%2520as%2520Google%2520software%2520(Google%2520Maps,%2520Street%2520View,%2520and%2520Earth).%2520For%2520more%2520information,%2520see%2520our%2520copyright%2520guidelines:%2520http:%2Fjournals.plos.org%2Fplosone%2Fs%2Flicenses-and-copyright.%250b%250bWe%2520require%2520you%2520to%2520either%2520(1)%2520present%2520written%2520permission%2520from%2520the%2520copyright%2520holder%2520to%2520publish%2520these%2520figures%2520specifically%2520under%2520the%2520CC%2520BY%25204.0%2520license,%2520or%2520(2)%2520remove%2520the%2520figures%2520from%2520your%2520submission:%250b%250ba.%2520You%2520may%2520seek%2520permission%2520from%2520the%2520original%2520copyright%2520holder%2520of%2520Figure%25201%2520to%2520publish%2520the%2520content%2520specifically%2520under%2520the%2520CC%2520BY%25204.0%2520license.%2520%250b%250bWe%2520recommend%2520that%2520you%2520contact%2520the%2520original%2520copyright%2520holder%2520with%2520the%2520Content%2520Permission%2520Form%2520(http:%2Fjournals.plos.org%2Fplosone%2Fs%2Ffile%3Fid=7c09%2Fcontent-permission-form.pdf)%2520and%2520the%2520following%2520text:%250b%E2%80%9CI%2520request%2520permission%2520for%2520the%2520open-access%2520journal%2520PLOS%2520ONE%2520to%2520publish%2520XXX%2520under%2520the%2520Creative%2520Commons%2520Attribution%2520License%2520(CCAL)%2520CC%2520BY%25204.0%2520(http:%2F%2Fcreativecommons.org%2Flicenses%2Fby%2F4.0%2F).%2520Please%2520be%2520aware%2520that%2520this%2520license%2520allows%2520unrestricted%2520use%2520and%2520distribution,%2520even%2520commercially,%2520by%2520third%2520parties.%2520Please%2520reply%2520and%2520provide%2520explicit%2520written%2520permission%2520to%2520publish%2520XXX%2520under%2520a%2520CC%2520BY%2520license%2520and%2520complete%2520the%2520attached%2520form.%E2%80%9D%250b%250bPlease%2520upload%2520the%2520completed%2520Content%2520Permission%2520Form%2520or%2520other%2520proof%2520of%2520granted%2520permissions%2520as%2520an%2520%2522Other%2522%2520file%2520with%2520your%2520submission.%250b%250bIn%2520the%2520figure%2520caption%2520of%2520the%2520copyrighted%2520figure,%2520please%2520include%2520the%2520following%2520text:%2520%E2%80%9CReprinted%2520from%2520%255bref%255d%2520under%2520a%2520CC%2520BY%2520license,%2520with%2520permission%2520from%2520%255bname%2520of%2520publisher%255d,%2520original%2520copyright%2520%255boriginal%2520copyright%2520year%255d.%E2%80%9D%250b%250bb.%2520If%2520you%2520are%2520unable%2520to%2520obtain%2520permiss/1/010f0195d4f70bf9-6543d57e-4a22-449b-8de4-f487457c29e1-000000/KQjRjZVLS913SYbFlnS7sIRF7FE8EpSESWorl1I1cbY=204)

Response: The maps in Fig 1 were created by “Browser GIS MANDARA JS” (<https://ktgis.net/mdrjs/index.html>), which provided free materials. The website mentions that users can use the materials without permission under the CC BY.

8. Please review your reference list to ensure that it is complete and correct. If you have cited papers that have been retracted, please include the rationale for doing so in the manuscript text, or remove these references and replace them with relevant current references. Any changes to the reference list should be mentioned in the rebuttal letter that accompanies your revised manuscript. If you need to cite a retracted article, indicate the article’s retracted status in the References list and also include a citation and full reference for the retraction notice.

Response: Thank you for your recommendations. We have carefully checked and confirmed that there are no retracted references in our manuscript. We also updated documents such as the annual report to the most recent versions whenever possible. We have highlighted the additional references in yellow in the reference list.

Additional Editor Comments:

In addition to addressing the reviewer’s comments, please ensure the following issue is resolved:

1. The reference list must be updated to include more recent studies published within the last five years. Currently, older references constitute 43% of the list, which exceeds the acceptable limit of 20%. This needs to be rectified to meet the required standards.

Response: Thank you for your valuable recommendation. We have carefully reviewed the references and revised the reference list to ensure that more than 80% of the cited studies were published within the last five years. We have updated 35 of the 42 references (83.3%) to publications in 2020 or later. We have highlighted the additional references in yellow in the reference list.

**Reviewers' comments:
Reviewer's Responses to Questions**

**Reviewer #1:**
1. The manuscript seeks to review the current surveillance reporting system for multi drug resistant Pseudomonas aeruginosa. The authors utilize both quantitative measures (reported case counts) and qualitative measures using questionnaires and interviews. The authors provide clear exclusion criteria for further analysis and provide a clear analysis of the results.
The interviewee size for the interview section is not mentioned, we do not know if this size is small, this limits the generalizability of the section. it is great that the authors clearly discuss the other limitations with their interviews, such as the fact that most interviewees were from one prefecture, I think the sample size is a key datapoint that should be mentioned. For example, if you interview only 50 individuals and the actual no of potentially appropriate interviewees is 5000, the sample size is too small to make any claims about what the true needs are.

Response: Thank you for your valuable suggestion. We interviewed 17 stakeholders, including three academic authorities and two public health officers working at one local infectious disease surveillance center, six physicians including infectious diseases specialists, two infection control nurses, two pharmacists, and two bacteriology technologists working at five hospitals. To clarify this point, we revised the descriptions in the Methods and Discussion sections as follows:

“On the basis of the information flow (**S1 Fig**), we conducted face-to-face, semi-structured key informant interviews with 17 stakeholders of the surveillance system between December 2, 2022, and August 24, 2023, to ask about its relevant attributes such as representativeness, acceptability, simplicity, and usefulness.” (Lines 192-195)

“Moreover, although the total number of participants was limited to 17, the interviewees included infectious diseases specialists and individuals from a variety of professional backgrounds.” (Lines 408-410)

We agree that some items of the interviews were difficult to generalize; however, we originally planned to collect the opinions regarding the challenges for the surveillance system. We actually obtained valuable insights into the areas for improvement of the surveillance system.

2. Also, some of the interview questions are binary, showing the actual count of respondents that favored one way or the other would provide quantitative data for government future efforts to improve. For example: "IX. Which do you think is better as a reportable disease: MDRP infections or carbapenem-resistant P. aeruginosa infections?" If 70% of respondents prefer CARP to MDRP, then it is clear the majority have this preferences vs say 51%. What is currently reported does not provide such pertinent details.

Response: Thank you for your insightful comments. We tried to make the interview results binary, but the majority of answers involved supplementary information. Many of the responses included valuable qualitative opinions that would be lost if simply reduced to binary choices. We wanted to avoid any potential misreading that could result from forcing such nuanced responses into a binary framework.

3. Finally, While the manuscript is fairly well written, the main text figures and table legends appear slapped in between the results and discussions section.

Response: We apologize for the time you took to understand the content. Submission guidelines of PLOS One mentioned that “Figure captions must be inserted in the text of the manuscript, immediately following the paragraph in which the figure is first cited (read order). Do not include captions as part of the figure files themselves or submit them in a separate document.” (<https://journals.plos.org/plosone/s/submission-guidelines>), and we followed these guidelines.

4. There are also a few small grammar errors that need to be addressed with the writing.
Response: Thank you for noting this. We have carefully rechecked the manuscript and corrected the grammatical errors accordingly.

**Reviewer #2:**

I must commend the authors for conducting this important evaluation on the surveillance of multidrug-resistant P. aeruginosa infections in Japan. The topic is highly relevant to public health and antimicrobial resistance (AMR) control efforts. The manuscript is well-structured, and the findings provide valuable insights into the strengths and weaknesses of the current surveillance system. While the study is strong overall, there are a few areas that could be clarified or expanded to further enhance the manuscript.
Response: We appreciate your thoughtful feedback. We have modified our article according to each of your valuable comments.

ABSTRACT
Line 38: Replace the word "the" to "this" to enhance the clarity
Response: Thank you for your comment. We have modified the sentence.

“We evaluated this surveillance system to verify the validity of this trend and explore future directions.” (Lines 37-38)

Line 40: It is important to highlight the key indicators assessed here in the methods.
Response: Thank you for your recommendation. We have described geographical representativeness as one of the key indicators as follows:

“A questionnaire was sent to identifiable DSSs (target DSSs) requesting data on accurate numbers of MDRP infections between 2018–2022 to assess attributes such as geographical representativeness.” (Lines 43-46)

Line 40: it is also unclear from this abstract what the geographical scope is. can you highlight this too?
Response: We have highlighted the geographical scope in the Methods and Results sections of the abstract as follows:

“A questionnaire was sent to identifiable DSSs (target DSSs) requesting data on accurate numbers of MDRP infections between 2018–2022 to assess attributes such as geographical representativeness.” (Lines 43-46)

“From 2013 to 2022, 1,666 cases of MDRP infections were reported by 463 target DSSs, which were scattered across the county.” (Lines 50-51)

Line 41: Its not clear what guidelines are been referred to here. Please be more specific. Is there a universal guideline for conducting cross-sectional studies?
Response: We stated that the US CDC developed the guidelines. While our quantitative analysis takes the form of a cross-sectional study, the guidelines themselves are intended for comprehensive surveillance evaluation, including qualitative assessments.

“We conducted a cross-sectional mixed method study mainly based on the guidelines published by the United States Centers for Disease Control and Prevention in 2001.” (Lines 40-41)

Line 42: It is essential to start by mentioning that this was a "mixed method study" before stating the quantitaive and qualitative components. This will aid better understanding of the methods
Response: Thank you for your comment. We have modified the sentence.

“We conducted a cross-sectional mixed-method study…” (Line 40)

Line 45: why was the quantitative aspect of this study done for different dates? 2013 and 2018? why not both arms of the study conducted from 2013 to 2022?

Response: Thank you for your insightful question. We initially considered a 10-year period for all survey phases, but based on feedback from several medical institutions, we limited the questionnaire survey to a 5-year period to improve the response rate.

Line 45: can you specify the kind of interviews that were conducted. Were they Key Informant interviews or Indepth interviews or....?
Response: Thank you for your comment. We have amended the sentences in the abstract to “semi-structured key informant interviews”.

“Additionally, we conducted as a qualitative method face-to-face, semi-structured key informant interviews with surveillance system stakeholders to assess its usefulness and challenges.” (Lines 46-48)

We have also modified sentences in the Methods section of the manuscript. We added a description stating that the six interviewees included infectious disease specialists, to further emphasize that these were key informant interviews as follows:

“On the basis of the information flow (**S1 Fig**), we conducted face-to-face, semi-structured key informant interviews with 17 stakeholders of the surveillance system between December 2, 2022, and August 24, 2023, to ask about its relevant attributes such as representativeness, acceptability, simplicity, and usefulness.” (Lines 192-195)

“We also selected six physicians including infectious diseases specialists, two infection control nurses, two pharmacists, and two bacteriology technologists working at five hospitals within the same prefecture (two TDSSs and three non-TDSSs), which were introduced by each local government.” (Lines 198-201)

Line 61: The word "may" should be replaced with "would" as this is a recommendation.
Response: Thank you for your comment. We have modified the word on Line 63.

INTRODUCTION
Lines 61 - 117: This introduction is great. However, it is important to include some information concerning the context of AMR in Japan generally and .
- can you provide the burden of AMR in Japan, particularly in healthcare facilities and community settings?

Response: Thank you for your valuable comments. We have added an explanation regarding the burden of AMR worldwide and in Japan at the beginning of the Introduction section. Although no studies have estimated the nationwide total number of AMR-related infections or deaths, a systematic review and meta-analysis reported that the burden was significantly higher in the AMR group compared to the non-AMR group in Japan. Furthermore, although antimicrobial use decreased after the release of the National Action Plan, the disease burden of bloodstream infections caused by major AMR pathogens did not change significantly. While AMR is particularly problematic in nosocomial infections—in which antimicrobial use and horizontal transmission play a significant role—we also cited a study that evaluated the burden of AMR in community-acquired infections. Thus, we have added the sentences and references in the beginning of the Introduction section as follows:

“Bacterial antimicrobial resistance (AMR) is a global concern and a significant public health threat. A comprehensive assessment of the global burden of AMR based on data from 204 countries and territories estimated 4.71 million deaths in 2021. Additionally, it is projected that by 2050, AMR could be associated with 8.22 million deaths globally, including 1.91 million directly attributable to AMR [1]. In Japan, although no studies have estimated the total number of AMR-related infections or deaths nationwide, the disease burden has been reported to be significantly higher in AMR infections including community-acquired infections compared to non-AMR infections [2,3]. After the release of the national action plan (NAP) on AMR, use of antimicrobials decreased between 2015 and 2021. Nevertheless, the disease burden of bloodstream infections caused by major AMR pathogens has not changed significantly [4].” (Lines 69-80)

1. GBD 2021 Antimicrobial Resistance Collaborators. Global burden of bacterial antimicrobial resistance 1990-2021: a systematic analysis with forecasts to 2050. Lancet. 2024;404:1199–1226.

2. Sakamoto Y, Yamauchi Y, Jo T, Michihata N, Hasegawa W, Takeshima H, et al. In-hospital mortality associated with community-acquired pneumonia due to methicillin-resistant Staphylococcus aureus: a matched-pair cohort study. BMC Pulm Med. 2021 Nov 3;21(1):345. doi: 10.1186/s12890-021-01713-1.

3. Matsumoto T, Yuasa A, Matsuda H, Ainiwaer D, Yonemoto N. Burden of Antimicrobial Resistance in Japan: A Systematic Literature Review and Meta-Analysis. Infect Dis Ther. 2024;13:1105–1125.

4. Tsuzuki S, Koizumi R, Matsunaga N, Ohmagari N. Decline in Antimicrobial Consumption and Stagnation in Reducing Disease Burden due to Antimicrobial Resistance in Japan. Infect Dis Ther. 2023;12:1823–1834.

-It may be also important to highlight in this introduction, any challenges to the existing surveillance efforts (e.g., underreporting, limited laboratory capacity, lack of standardized protocols e.t.c). this will help to paint a better picture of the problem. Because as it stands, your focus is on whether the downward trend accurately represents the actual trend in MDRP infections but little is said about existing challenges which could be linked to this trend. Are there previous studies or reports on MDR P. aeruginosa infections in Japan?

Response: Thank you for your valuable comments. We added a statement in the manuscript regarding a challenge in the surveillance of MDRP infections in Japan—namely, the potential for reporting errors due to discrepancies between the case definitions used for reporting and the CLSI resistance criteria commonly used in clinical practice. We believe that by assessing the accuracy of these reports, it will be possible to confirm whether the observed decreasing trend of MDRP infections is reliable. Thus, we have revised the sentences as follows:

“The susceptibility criteria used by NESID and JANIS for sentinel surveillances differ somewhat from the clinical breakpoints established by the Clinical and Laboratory Standards Institute (CLSI) and the European Committee on Antimicrobial Susceptibility Testing (EUCAST) (S1 Table) [25,26]. These discrepancies may often cause confusion among clinicians and lead to inaccurate reporting.” (Lines 123-128)

Lines 117 - 119: Are there existing documented gaps in the current surveillance system for MDR P. aeruginosa to justify this evaluation?(e.g., lack of real-time data, poor integration with hospital reporting systems e.t.c). This is important to justify why evaluating the surveillance system is essential for strengthening AMR monitoring and improving infection control strategies not just only because you want to know whether the trend accurately represents actual trend.

Response: We believe that there has been no documentation on the existing gaps, and this is the first report to document these gaps. As mentioned in the previous response, we considered the discrepancy between the case definitions of MDRP infections used in surveillance and the definitions of resistance used in clinical practice to be a challenge. Additionally, our study aimed to identify possible challenges for inaccuracy, if any. Actually, we also found that there were other challenges through the interviews.

Lines 122 - 124: Clearly state the main objective of this study here.
is the objective to “evaluate the effectiveness, validity, sensitivity, and limitations of the surveillance system for multidrug-resistant Pseudomonas aeruginosa infections in Japan"?
Optionally, break it down into specific objectives, such as:
- Assessing the completeness, timeliness, and accuracy of surveillance data.
- Identifying gaps in case detection and reporting.
e.t.c
the way it is written, its not clear what the objectives are.

Response: Thank you for your insightful recommendations. We agree that the objectives were unclear and included some duplication between the Introduction and Methods sections. Thus, we have amended the objectives as follows:

“Our study aimed to evaluate whether NESID accurately captures the nationwide trend in MDRP infections and determine possible challenges related to inaccuracy, if any, for better control of drug-resistant *P. aeruginosa* through improvement of the current surveillance system.” (Lines 136-139)

METHODS
Line 126: Should be "methods" not "method"
Response: Thank you for noting this. We have corrected the wording. (Line 141)

Line 129: more accurately put as "Mixed methods"

Response: Thank you for your comment. We have revised the sentence as follows:

“We conducted a cross-sectional study in 2023 using a mixed-methods approach that incorporated both quantitative and qualitative analyses.” (Lines 143-144)

Line 129 - 134: The Aim should not be in the methods but in the introduction or as a separate subsection before the methods. Please revise accordingly

Response: Thank you for your valuable comment. We deleted the Aim in the Methods section and added the sentences to the Introduction section as follows:

“Our study aimed to evaluate whether NESID accurately captures the nationwide trend in MDRP infections and determine possible challenges related to inaccuracy, if any, for better control of drug-resistant *P. aeruginosa* through improvement of the current surveillance system.” (Lines 136-139)

Lines 144 - 157: It is important to provide more specific context about the Study Setting and Population to allow better understanding of the results
- please describe the geographical scope (e.g., national, selected states, or specific healthcare facilities in Japan).
- Mention the type of healthcare facilities involved (e.g., tertiary hospitals, secondary health centers, surveillance units).

- Define the target population (e.g., patients with laboratory-confirmed MDR P. aeruginosa infections, healthcare providers, surveillance officers).

Response: Thank you for your comments. This sentinel surveillance is conducted at the national level, with at least one designated sentinel site (DSS) selected in each of the 47 prefectures across Japan. A DSS typically has the capacity to hospitalize at least 300 patients and includes departments of internal medicine and surgery; however, sites are selected regardless of the type of healthcare facility, such as tertiary hospitals or secondary health centers. DSSs report AMR bacterial infections, including MDRP, and the target population of this surveillance comprises patients who visit or are hospitalized at these DSSs. Surveillance data are reported by representatives of each hospital. Therefore, we also conducted interviews with these representatives as part of the evaluation. Thus, we have revised the sentences as follows:

“In Japan, which is divided into 47 prefectures, each prefectural government selects at least one designated sentinel site (DSS) per secondary medical area, which is a medical administrative area, regardless of the type of healthcare facility, such as tertiary hospitals or secondary health centers, under the Medical Care Law. The DSS generally is capable of hospitalizing at least 300 patients and has internal medicine and surgery departments, from which particular AMR bacterial infections including MDRP infections are reported [20]. Reporting of AMR infections from DSSs is done by the hospital administrators.” (Lines 160-167)

Line 163: can you describe where this data is stored? from where was it accessed?

Response: Thank you for your important question. NESID data are stored on a confidential, password-protected server in accordance with Japanese law, and we used it for secondary purposes. The following text is included:

“We accessed the NESID data for research purposes on January 11, 2023.” (Lines 181-182)

LIne 163: it is important to provide more details of this questionnaire
- was it adopted or adapted (or even developed by the researchers) - please include reference for the source

- how was the questionnaire validated?

- who were the respondents of the questionnaire

- what language was the questionnaire in?

- what form was the questionnaire (paper or electronic) - if electronic, was a certain platform or tool used e.g - Goodle forms, microsoft forms, Kobotoolbox e.t.c)

- who were the respondents??????
Response: Thank you for your valuable comments. We developed the questionnaire in consultation with expert surveillance officers, well-experienced clinicians, and clinical microbiology technologists. Although we have not validated the questionnaire, we piloted a questionnaire survey among selected surveillance officers and clinicians and revised the questionnaire accordingly. This questionnaire was paper-based and written in Japanese.

We sent the questionaries to the hospital administrators or infection control teams, and actual responses were primarily provided by clinical microbiology technologists (55.9%), followed by nurses (28.0%), doctors (5.9%), and pharmacists (2.1%). Thus, we have revised or added the sentences as follows:

“On February 1, 2023, we sent a questionnaire that we had developed (**S1 Appendix**) to the administrators or infection control teams in the TDSSs to request the accurate number of MDRP infections occurring at each site between January 2018 and December 2022. The questionnaire was paper based and written in Japanese.” (Lines 182-186)

“The respondents were primarily clinical microbiology technologists (55.9%), followed by nurses (28.0%), doctors (5.9%), and pharmacists (2.1%).” (Lines 251-253)

Line 180; A questionnaire is not typically used for interviews. Qualitative data is not usually collected using questionnaires. can you explain what type of interview was done and why you decided to use a questionnaire instead of an interview guide?
Response: Thank you for your valuable comment and question. We apologize for the misleading information caused by an error in our English. The interviews were conducted as face-to-face key informant interviews based on the interview guides (**S2 Appendix**). Additionally, these interviews were semi-structured. We have revised the title of S2 Appendix and the text as follows:

“Well-trained interviewers conducted each interview by using interview guides (**S2 Appendix**) that were reviewed by experienced physicians and clinical microbiology technologists.” (Lines 201-203)

Line 182:

- where were these physicians and technologists based?
Response: We asked the doctors and clinical microbiology technologists in non-TDSSs to review the interview guides.

- can you provide more details about the tool used for the interview and how the qualitative data was collected. was this audio recorded? what tools were used? Was the interview structured or unstructured. It is important to be thoruogh with your methodology to allow reproducibility and clarity

Response: Thank you for your valuable questions. We did not record the interview because we were unable to obtain permission from the interviewees; however, at least two well-trained interviewers conducted the interview to ensure that no details were missed. As shown in the attached **S2 appendix**, the interview was semi-structured.

“On the basis of the information flow (**S1 Fig**), we conducted face-to-face, semi-structured key informant interviews with 16 stakeholders of the surveillance system between December 2, 2022, and August 24, 2023, to ask about its relevant attributes such as representativeness, acceptability, simplicity, and usefulness.” (Lines 192-195)

“Well-trained interviewers conducted each interview by using interview guides (**S2 Appendix**) that were reviewed by experienced physicians and clinical microbiology technologists.” (Lines 201-203)

“Although we did not electronically record the interviews, at least two well-trained interviewers conducted each interview to ensure that no details were missed.” (Lines 204-205)

Line 184 - 197: There should be a separate subsection (not Analysis) to define the variables collected, such as the surveillance indicators (e.g., completeness, timeliness, sensitivity, specificity). This should not come under analysis
Under analysis section you should describe the statistical methods used to analyze quantitative data (e.g., descriptive statistics, proportions, trend analysis). It is also important to mention any qualitative analysis conducted (e.g., thematic analysis for key informant interviews). This section is completely silent on how you analysed the qualitative data
please specify the software used for data analysis (e.g., SPSS, Stata, R, NVivo e.t.c).
Response: Thank you for your important comments. We have defined each of the attributes/surveillance indicators as “perspectives” in Table 1, which is cited in the “Study design” section. We conducted descriptive statistics for quantitative analysis by using R version 4.4.3. Thus, we have added the following sentences:

“We calculated the mean age and standard deviation, and sex distribution, of the patients using the NESID data.” (Lines 209-210)

“R version 4.4.3 was used for all of the calculations [30].” (Lines 222-223)

We did not analyze our qualitative data by using applications that the reviewer listed. Thanks to your valuable comments, we learned the new methods of qualitative data analysis and would like to follow your suggestions when we have the chance to conduct the next qualitative survey including surveillance evaluation of other notifiable diseases.

RESULTS
Line 238: Table 2- please indicate the appropriate column header here.
Response: We have added the header “Types of TDSSs” to Table 2.

Line 256: Table 3- please indicate the appropriate column header here.
Response: We have added the header “Notifications by types of TDSSs” to Table 3.

Line 259: Qualitative findings are typically presented under thematic headings, supported by direct quotes from respondents. There is no description in the methods as to how the qualitative data was collected and analysed. Clearly state how qualitative data were analyzed (e.g., thematic analysis using NVivo or manual coding). Also, describe the process of identifying themes, including how transcripts were reviewed, coded, and categorized. please reconcile with the methods.
Response: Thank you for your valuable comments. As we mentioned in the response to your previous comments, because the guidance published by US CDC did not mention qualitative data analysis, we did not conduct the analysis by using the applications that you recommended.

DISCUSSION
Line 289: Can you compare your findings to similar studies from Japan, Asia, or global surveillance reports
Response: Thank you for your insightful recommendation. Regarding another surveillance dataset in Japan, as described in the Discussion section, the JANIS data (laboratory division) showed a similarly declining trend in the frequency of MDRP among collected specimens as of 2023 (References 23 and 24). EARS-Net, which conducts AMR surveillance in the EU/EEA, also reported a decrease in the detection frequency of MDRP in invasive infections between 2017 and 2023 (Reference 16). In contrast, in the U.S., data published in 2024 showed that MDRP infections increased in 2021–2022 compared to 2019, despite a previously observed decreasing trend (References 14 and 15).

However, as shown in the Discussion section (in Lines 368-370) and Table 4, we believe it is important to recognize a challenge in MDRP surveillance: trends cannot be reliably compared when the classes of antibiotics under surveillance differ. The case definitions for MDRP used in NESID and JANIS are based on resistance to three specific classes of antibiotics, including aminoglycosides, which are often still effective in other countries. This makes international comparisons difficult. Thus, although we did not compare the trends of MDRP infections with those in other countries or regions, we have added a statement in the Introduction section referring to the trend of MDRP infections in the U.S. as follows:

“Furthermore, data published in 2024 showed that MDRP infections increased in 2021–2022 compared to 2019, despite a previously observed decreasing trend [15].” (Lines 97-99).

Line 298: its not clear how you triangulated the results from the quantitative with your qualitative results. can you explain the areas of overlap?

Response: Thank you for your valuable question. As mentioned in Table 1, each attribute was evaluated quantitatively, qualitatively, or both ways. In particular, representativeness and PPV were evaluated by both methods. Although these evaluations seemed to be overlapping, we evaluated them from different angles.

Line 311 - 314: where did this data come from? did you collect this information from existing literature or you did a review? Please include the reference for the sources of these information. What part of your methods covered this area? please clarify

Response: Thank you for your questions. This is our original idea based on the results of our evaluation. There are no reports discussing this matter, which was a major reason for conducting our evaluation. Thanks to your comments we realized that we did not mention creating this table. Thus, we have added the following sentence in the Methods section.

“Additionally, on the basis of this evaluation, we listed the advantages and disadvantages of possible surveillance systems of drug-resistant *P. aeruginosa* infections.” (Lines 149-151)

Line 368: what are the strengths of your study? This would be a good place to include them.

Response: Thank you for your recommendation. We believed that the strength of our study is that this is the first report to evaluate the surveillance system for drug-resistant *P. aeruginosa* infections comprehensively and to discuss the advantages and disadvantages of potential candidate systems. Thus, we have added the following sentence.

“The strength of our study is that it is, to our knowledge, the first report to comprehensively evaluate surveillance systems for drug-resistant *P. aeruginosa* infections and to discuss the advantages and disadvantages of potential candidate systems.” (Lines 400-403)

Map: This is a good map. However, it is important to include the scale as well as cardinal points/North arrow. Clearly label the legend too.

Response: Thank you for your comment. We have modified figures and inserted the scale and cardinal points/North arrow. Additionally, we have added labels to the legends and inserted a sentence in the figure caption as follows:

“The blue color scale indicates the distribution across each percentage range.” (Lines 267-268)

**Reviewer #3:**

The paper is well written and should be published. The use of longitudinal quantitative data over a wide period of time is commendable. And combining this with questionnaire and interview data makes it robust.

Response: We appreciate your thoughtful feedback. We have modified our article according to each of your valuable comments.

#1 Line 83. The U.S. Centers for Disease Control and Prevention (CDC) has estimated 32,600 cases and 2,700 deaths due to MDRP infections annually and classified them as a serious public health threat, calling for the need for prompt and sustained action in 2019 [11]. Can the authors provide more Japan specific data vis a vis MDRP epidemiology?

Response: Thank you for your insightful comments. There are very limited data on MDRP infections in Japan. The only information about MDRP infections are in the NESID data (<https://id-info.jihs.go.jp/surveillance/idwr/annual/2022/index.html>, in Japanese), and we cited them on Line 131. Unfortunately, we lack the information regarding the number of deaths due to MDRP infections in Japan. Instead, we have added sentences to the Introduction section that provide an overview of the disease burden of AMR in Japan as follows:

“In Japan, although no studies have estimated the total number of AMR-related infections or deaths nationwide, the disease burden has been reported to be significantly higher in AMR infections including community-acquired infections compared to non-AMR infections [2,3]. After the release of the national action plan (NAP) on AMR, use of antimicrobials decreased between 2015 and 2021. Nevertheless, the disease burden of bloodstream infections caused by major AMR pathogens has not changed significantly [4].” (Lines 73-80)

#2 For the results and observations prior to this study, if possible, can the authors provide a demographic breakdown of how MDRP affects the population- age, sex, immune status, etc. How many of these MDRPs were community or nosocomially acquired?

Response: Thank you for your recommendation. We have added the sentences and supplementary figure regarding age and sex based on our data as follows:

“The mean age (± standard deviation) of the reported patients was 71.6 ± 17.6 years, and 1,168 patients (69.9%) were male. The number of cases reported as MDRP infections showed a decreasing trend for both sexes, especially among the elderly (**S3 Fig**).” (Lines 232-235)

“**S3 Fig. Trend in the number of cases reported to NESID as MDRP infections by age group between 2013 and 2022.** The number of reported cases of MDRP infection showed a decreasing trend in both males (A) and females (B), especially among the elderly. MDRP, multidrug-resistant *Pseudomonas aeruginosa*; NESID, National Epidemiological Surveillance of Infectious Diseases.” (Lines 661-665)

However, the NESID system does not collect data on patients’ immune status or whether MDRP infections are community-acquired or hospital-acquired, making these difficult to distinguish. As a reference, according to the JANIS data (laboratory division), the proportion of MDRP detected per number of patients who submitted specimens in 2023 was 0.004% (134/3,441,898 samples) for outpatient specimens (Reference 24), and 0.02% (651/3,089,112 samples) for inpatient specimens (Reference 23), which is approximately five times higher. Therefore, we inferred that hospital-acquired infections likely account for a greater proportion of MDRP infections as well.

#3 Can the authors provide data or explain how the decreasing trend they observed is true and not a product of some unaccounted for confounders? Is it not possible that give the period of time covered by the study, that there might have been a change in the commitment and efficiency of those in charge of the surveillance over the years? Depending on the answers, these new insights could be added to the limitations.

Response: As you rightly point out, there may be unaccounted confounders. Although there have been no changes in the national surveillance system itself, changes in the conduction of surveillance at individual medical institutions—such as those due to a change in personnel—may have occurred. Thus, according to your recommendations, we have added this point in the Discussion section as follows:

“Third, although there have been no changes in the national surveillance system for MDRP infection itself, changes in the commitment and efficiency of those in charge of surveillance at individual medical institutions may have occurred over the years.” (Lines 413-416)
