# Supplementary material for: What we can and cannot see from the surveillance for drug-resistant Pseudomonas aeruginosa—Findings from the evaluation of a surveillance system for multidrug-resistant P. aeruginosa infections in Japan
Source: PLoS One. 2025 Aug 4;20(8):e0329635. doi: 10.1371/journal.pone.0329635 (PMC12321080; doi:10.1371/journal.pone.0329635)
Supplement: Attachment — Submitted filename: MDRP_Response to editorial offices commens_20250708.docx [file pone.0329635.s014.docx]

**Response to the editorial comments**
 1. We note that Figure 1 in your submission contain [map/satellite] images which may be copyrighted. All PLOS content is published under the Creative Commons Attribution License (CC BY 4.0), which means that the manuscript, images, and Supporting Information files will be freely available online, and any third party is permitted to access, download, copy, distribute, and use these materials in any way, even commercially, with proper attribution. For these reasons, we cannot publish previously copyrighted maps or satellite images created using proprietary data, such as Google software (Google Maps, Street View, and Earth). For more information, see our copyright guidelines: <http://journals.plos.org/plosone/s/licenses-and-copyright>.

We require you to either (1) present written permission from the copyright holder to publish these figures specifically under the CC BY 4.0 license, or (2) remove the figures from your submission:

a. You may seek permission from the original copyright holder of Figure 1 to publish the content specifically under the CC BY 4.0 license.
We recommend that you contact the original copyright holder with the Content Permission Form (<http://journals.plos.org/plosone/s/file?id=7c09/content-permission-form.pdf>) and the following text:
“I request permission for the open-access journal PLOS ONE to publish XXX under the Creative Commons Attribution License (CCAL) CC BY 4.0 (<http://creativecommons.org/licenses/by/4.0/>). Please be aware that this license allows unrestricted use and distribution, even commercially, by third parties. Please reply and provide explicit written permission to publish XXX under a CC BY license and complete the attached form.”

Please upload the completed Content Permission Form or other proof of granted permissions as an "Other" file with your submission.

In the figure caption of the copyrighted figure, please include the following text: “Reprinted from [ref] under a CC BY license, with permission from [name of publisher], original copyright [original copyright year].”

b. If you are unable to obtain permission from the original copyright holder to publish these figures under the CC BY 4.0 license or if the copyright holder’s requirements are incompatible with the CC BY 4.0 license, please either i) remove the figure or ii) supply a replacement figure that complies with the CC BY 4.0 license. Please check copyright information on all replacement figures and update the figure caption with source information. If applicable, please specify in the figure caption text when a figure is similar but not identical to the original image and is therefore for illustrative purposes only.
The following resources for replacing copyrighted map figures may be helpful:

USGS National Map Viewer (public domain): <http://viewer.nationalmap.gov/viewer/>

The Gateway to Astronaut Photography of Earth (public domain): <http://eol.jsc.nasa.gov/sseop/clickmap/>

Maps at the CIA (public domain): <https://www.cia.gov/library/publications/the-world-factbook/index.html> and <https://www.cia.gov/library/publications/cia-maps-publications/index.html>
NASA Earth Observatory (public domain): <http://earthobservatory.nasa.gov/>
Landsat: <http://landsat.visibleearth.nasa.gov/>
USGS EROS (Earth Resources Observatory and Science (EROS) Center) (public domain): [http://eros.usgs.gov/#](http://eros.usgs.gov/)
Natural Earth (public domain): <http://www.naturalearthdata.com/>

Response: Thank you for your comments. As requested, we have re-created a figure that complies with the CC BY 4.0 license based on data ‘Global Map Japan’, Technical Report of the Geospatial Information Authority of Japan, D1-No.576 (https://www.gsi.go.jp/kankyochiri/gm_japan_e.html). This data is licensed under the Public Data License (Version 1.0), which is stated to be compatible with CC BY 4.0 (https://www.gsi.go.jp/kikakuchousei/kikakuchousei40182.html and https://www.digital.go.jp/resources/open_data/public_data_license_v1.0, in Japanese). Thus, in accordance with the Licenses and Copyright policy of PLOS One, we have added the following statement to the legend of Fig 1.

“We created the map based on data ‘Global Map Japan’, Technical Report of the Geospatial Information Authority of Japan, D1-No.576 (<https://www.gsi.go.jp/kankyochiri/gm_japan_e.html>)” (Lines 272-274)

**↓**

**We revised the description of this part again based on the editorial office’s comments on 7 July as below;**

Fig 1. Distribution of 463 TDSSs. A. The percentage of TDSSs among all hospitals in each prefecture. B. The percentage of hospitals with 300 beds or more among the TDSSs in each prefecture. The blue color scale indicates the distribution across each percentage range. We created the map by editing data ‘Global Map Japan’. TDSS, target designated sentinel site.

Source: ‘Global Map Japan (Technical Report of the Geospatial Information Authority of Japan, D1-No.576)’(Geospatial Information Authority of Japan) (https://www.gsi.go.jp/kankyochiri/gm_japan_e.html) PDL1.0(https://www.gsi.go.jp/kikakuchousei/kikakuchousei40182.html) (accessed on July 7, 2025)

We highlighted green for the change.

2. Please upload a Response to Reviewers letter which should include a point by point response to each of the points made by the Editor and / or Reviewers. (This should be uploaded as a 'Response to Reviewers' file type.) Please follow this link for more information: [http://blogs.PLOS.org/everyone/2011/05/10/how-to-submit-your-revised-manuscript/](http://blogs.plos.org/everyone/2011/05/10/how-to-submit-your-revised-manuscript/)

Response: Thank you for your comment. We have uploaded a Response to Reviewers letter as a `Response to Reviewers’ file type.

3. Before we proceed, we’ll require some additional clarification to ensure your submission adheres to the PLOS ONE Data Availability policy (<https://journals.plos.org/plosone/s/data-availability>).

Please note that the data we ask authors to share is not necessarily the full and/or raw data set collected in the course of their research, but rather the "minimal data set", which we define as consisting of the data set used to reach the conclusions drawn in the manuscript with related metadata and methods, and any additional data required to replicate the reported study findings in their entirety.

Depending on the nature of your study, this can include any or all of the following:
- The values behind the means, standard deviations and other measures reported;
- The values used to build graphs;
- The points extracted from images for analysis.

For studies involving third-party data (i.e., data not owned or collected by the authors), we encourage authors to share any data specific to their analyses that they can legally distribute. PLOS recognizes, however, that authors may be using third-party data they do not have the rights to share. When third-party data cannot be publicly shared, authors must provide all information necessary for interested researchers to apply to gain access to the data in the text of the Data Availability statement. This statement will be published online alongside your article should it be accepted for publication.

For any third-party data that the authors cannot legally distribute, please provide the following information in an updated and complete Data Availability statement:

a) Please kindly explain in detail how others can access the minimal datasets you used in this study from the third-party websites you accessed (e.g., <https://id-info.jihs.go.jp/surveillance/idwr/annual/2022/index.html> and <https://id-info.jihs.go.jp/surveillance/idwr/annual/2022/archive/index.html)>. Please provide any relevant information necessary to gain immediate access to the specific minimal datasets used in your work (e.g. contact information, request instructions, specific URLs, dataset titles, etc.).

Response: Thank you for your valuable comments. As requested, we have prepared the additional minimal datasets: for Supplementary Figure 3, see S2 Table, and for Figure 1, see S3 Table. Additionally, the datasets originally submitted in relation to the questionnaire survey have been included as S4 Table and S5 Table and inserted into the relevant sections of the manuscript.

Since we have submitted datasets equivalent to the information available on the previously introduced websites, we have removed the mention of third-party data from the "Availability of Data and Materials" section. As the readers directly obtain the third-party data, the following note has been included in S2 Table:

“The third-party data of NESID are on the public Japanese website at https://id-info.jihs.go.jp/surveillance/idwr/annual/2022/index.html and https://id-info.jihs.go.jp/surveillance/idwr/annual/2022/archive/index.html. Readers can access these websites to download Table 12-1 for each year. By selecting the sheet on multidrug-resistant *P. aeruginosa*, they can obtain information equivalent to that presented in this table, along with the most current data available. It should be noted, however, that these data have undergone partial revisions since we accessed the NESID dataset.”

Thus, we have revised the sentences in the "Availability of Data and Materials" section as follows.

“The minimum datasets used in the analysis are provided as supplementary materials: S2-S5 Table.” (Lines 471-472)

b) Please clarify in detail whether interested researchers can replicate your study findings in their entirety by directly obtaining the data from the third party sources in your Data Availability statement and following the information outlined in your Methods section.

Response: Thank you for your suggestion. In response to comment a), we have submitted additional minimal datasets as S3 and S4 Tables to facilitate readers’ replication. Additionally, the datasets originally submitted in relation to the questionnaire survey have been included as S4 and S5 Tables and inserted into the relevant sections of the manuscript. Moreover, to maximize reproducibility of the results, we added the following sentence to the “Analysis” section and included two additional references:

"We calculated the mean age with standard deviation and sex distribution of the patients using the NESID data and calculated the distribution of TDSSs as a percentage of all hospitals in each prefecture based on public data [30] and presented the distribution of TDSSs that are hospitals with 300 beds or more." (Lines 210-212)

30. e-Stat. 2021 Medical Facility (Status) Survey Nationwide. Number of hospitals by year and prefecture (in Japanese). 2023. Available from: https://www.e-stat.go.jp/dbview?sid=0002013722 [Accessed 24 Jun 2025]

32. e-Stat. 2021 Medical Facility (Status) Survey Nationwide. Number of hospitals, by type of beds and size of beds (in Japanese). 2023. Available from: https://www.e-stat.go.jp/dbview?sid=0002013428 [Accessed 24 Jun 2025]

While we made efforts to maximize reproducibility, the data used in our study includes unpublished national data; therefore, some information is not contained in the minimum datasets we submitted, making it difficult for readers to fully replicate our study results. This situation remains the same even if they obtain the information from the originally provided websites as third-party sources—the published NESID datasets.

If readers require more raw data containing more detailed information to replicate our study results for public health purposes, a request should be directed to the corresponding author. The corresponding author will contact the appropriate national authorities, including the Ministry of Health, Labour and Welfare, and the data will be provided upon approval.

Thus, we have added the following sentences in “Availability of data and materials” section.

“The minimum datasets used in the analysis are provided as supplementary materials: S2-S5 Table.

If access to raw data containing more detailed information is required for public health purposes, a request should be directed to the corresponding author. The corresponding author will contact the appropriate national authorities, including the Ministry of Health, Labour and Welfare, and the data will be provided upon approval.” (Lines 471-476)

c) Please also confirm whether the authors had any special access privileges that others would not have when attempting to access the minimal data you used from these third party sources.

Alternatively, if there are no restrictions, please upload the complete minimal anonymized data set necessary to replicate your study findings to a stable, public repository and provide us with the relevant URLs, DOIs, or accession numbers.

For a list of recommended repositories, please see: <https://journals.plos.org/plosone/s/recommended-repositories>

You also have the option of uploading the data as Supporting Information files, but we would recommend depositing data directly to a data repository if possible.

We will update your Data Availability statement on your behalf to reflect the information you provide.

Response: Thank you for your important suggestions. Response to comments a) and b), we uploaded the minimal data set as supplementary materials: S2-S5 Table. Additionally, the third-party sources are publicly available.

Because some of the national data is unpublished, we have noted in "Availability of data and materials" that if readers require the raw data, they should contact the corresponding author.
